# Regulation of *de novo* and maintenance DNA methylation by DNA methyltransferases in postimplantation embryos

**DOI:** 10.1016/j.jbc.2024.107990

**Published:** 2024-11-13

**Authors:** Zhen Xu, Jiajia Shi, Qian Chen, Shuting Yang, Zilin Wang, Biao Xiao, Zhijian Lai, Yumeng Jing, Yilin Li, Xiajun Li

**Affiliations:** School of Life Science and Technology, ShanghaiTech University, Shanghai, China

**Keywords:** DNA methylation, *de novo* methylation, maintenance methylation, DNMT3A, DNMT3B, DNMT1, genomic imprinting, imprinting control region (ICR), whole-genome bisulfite sequencing (WGBS), implantation

## Abstract

DNA methylation is mainly catalyzed by three DNA methyltransferase (DNMT) proteins in mammals. Usually DNMT1 is considered the primary DNMT for maintenance DNA methylation, whereas DNMT3A and DNMT3B function in *de novo* DNA methylation. Interestingly, we found DNMT3A and DNMT3B exerted maintenance and *de novo* DNA methylation in postimplantation mouse embryos. Together with DNMT1, they maintained DNA methylation at some pluripotent genes and lineage marker genes. Germline-derived DNA methylation at the imprinting control regions (ICRs) is stably maintained in embryos. DNMT1 maintained DNA methylation at most ICRs in postimplantation embryos. Surprisingly, DNA methylation was increased at five ICRs after implantation, and two DNMT3 proteins maintained the newly acquired DNA methylation at two of these five ICRs. Intriguingly, DNMT3A and DNMT3B maintained preexisting DNA methylation at four other ICRs, similar to what we found in embryonic stem cells before. These results suggest that DNA methylation is more dynamic than originally thought during embryogenesis including the ICRs of the imprinted regions. DNMT3A and DNMT3B exert both *de novo* and maintenance DNA methylation functions after implantation. They maintain large portions of newly acquired DNA methylation at variable degrees across the genome in mouse embryos, together with DNMT1. Furthermore, they contribute to maintenance of preexisting DNA methylation at a subset of ICRs as well as in the CpG islands and certain lineage marker gene. These findings may have some implications for the important roles of DNMT proteins in development and human diseases.

DNA methylation is the most common epigenetic modification on the genetic material DNA (1, 2, 3, 4, 5). In mammals, DNA methylation occurs mainly at the CpG sites in which a methyl group is added to the fifth position of the cytosine residue (6, 7, 8). CpG methylation is catalyzed by four DNMT proteins (DNMT1, DNMT3A, DNMT3B, and DNMT3C) in mouse (1, 9). DNMT3C is primarily expressed in the male germ cells of mice and therefore it does not appear to affect DNA methylation in mouse embryos (9, 10, 11). DNMT1 is considered to be the DNMT protein responsible for maintenance DNA methylation in mouse embryos and other somatic cells (3). On the other hand, DNMT3A and DNMT3B act in *de novo* DNA methylation in postimplantation embryos and other cell types (12). A related protein DNMT3L does not have catalytic activity on its own, but it can help DNMT3A and DNMT3B in *de novo* DNA methylation (13). DNA methylation may be lost through replication-dependent passive demethylation (14, 15). It can be also removed by oxidative demethylation mediated by TET proteins (16, 17).

DNMT1 may have some roles in *de novo* DNA methylation, although DNMT1 is primarily involved in maintenance DNA methylation during mouse embryonic development (5, 18, 19, 20). It has been reported that DNMT3A and DNMT3B may contribute to the maintenance DNA methylation in mouse embryonic stem (ES) cells (5, 21, 22, 23). Indeed, we found that DNMT3A and DNMT3B maintained DNA methylation, together with DNMT1, in the repeats and other genomic sequences in mouse ES cells (24). In this study, we have obtained similar findings for the maintenance DNA methylation function of DNMT3A and DNMT3B at the repeats, retroviral elements, genic, and intergenic regions in mouse embryos after we have examined the effects on DNA methylation upon loss of two DNMT3 proteins in the postimplantation embryos. As expected, they also play important roles in *de novo* DNA methylation during implantation.

To date, there are about 200 known imprinted genes in mice, with many of them conserved in humans (25, 26, 27). Most of the known imprinted genes are clustered in 24 known imprinted regions (28, 29, 30). The imprinted genes in a cluster are coregulated by a *cis*-acting imprinting control region (ICR) (31, 32). Differential DNA methylation at the ICR is required for parent-of-origin-dependent mono-allelic expression of the imprinted genes located in this imprinted region that may be lost in some human diseases (28, 33, 34). DNA methylation at the ICRs is established in the germ cells in which most ICRs acquire DNA methylation on the maternal chromosome during oogenesis, whereas DNA methylation is established at three ICRs (*H19*, IG-DMR, and *Rasgrf1*) on the paternal chromosome during spermatogenesis (33, 35). Differential DNA methylation at the ICRs is reconstituted upon fertilization and is thought to be stably maintained thereafter during embryogenesis, probably through recruitment of DNMTs to the ICRs by the complex of ZFP57 and KAP1 (36, 37, 38, 39). *Zfp57* is essential for maintaining DNA methylation in the most imprinted regions in mouse embryos (40, 41). It exhibits maternal effect and sexually dimorphic effects on DNA methylation at a small subset of imprinted regions as well as on expression of some corresponding imprinted genes (40). Loss of *Zfp57* causes loss of DNA methylation imprint at the target imprinted regions, which leads to allelic expression switch of the target imprinted genes in mouse embryos (41).

DNA methylation was maintained by DNMT1 at most ICRs in postimplantation embryos according to our current study. However, we found DNA methylation could be increased at a few ICRs after implantation. Furthermore, DNMT3A and DNMT3B contributed to the maintenance of DNA methylation at a subset of ICRs. Consistent with these findings, DNMT3A and DNMT3B were also found to maintain DNA methylation at a subset of ICRs in mouse ES cells in our previously published study (24). Similar findings were obtained when individual CpG sites were analyzed at these ICRs.

## Results

### Maintenance of whole-genome DNA methylation in postimplantation mouse embryos

**Figure 1:**
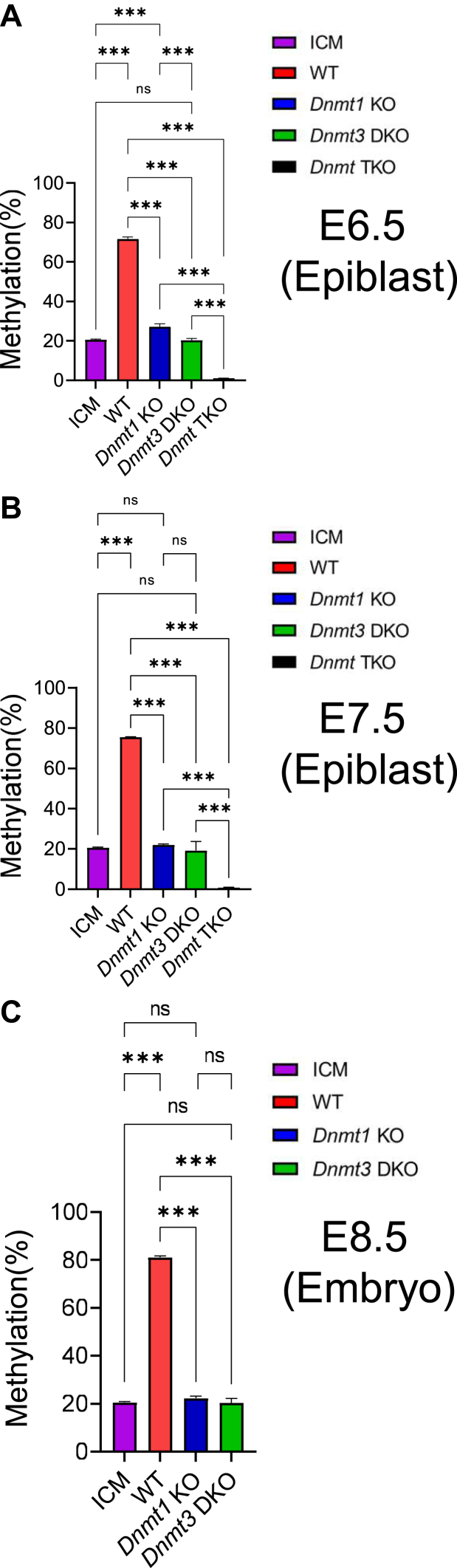
**Two DNMT3 proteins, together with DNMT1, maintained DNA methylation in the postimplantation embryos**. DNA methylation was quantified at the whole-genome level for the genomic DNA samples derived from the ICM of the WT E3.5 blastocysts, the epiblasts of WT, and *Dnmt*, 1KO mutant E6.5-E7.5 embryos, as well as WT and *Dnmt*, 1KO mutant E8.5 embryos that were shown on the horizontal axis. *Vertical* axis, % of the whole-genome DNA methylation. *A*, whole-genome DNA methylation was quantified for the ICM of WT blastocysts, the epiblasts of WT, *Dnmt1* mutant (*Dnmt1* KO), *Dnmt3* double mutant (*Dnmt3* DKO), and *Dnmt* triple mutant (*Dnmt* TKO) E6.5 embryos. *B*, whole-genome DNA methylation was quantified for the ICM of WT blastocysts, the epiblasts of WT, *Dnmt1* mutant (*Dnmt1* KO), *Dnmt3* double mutant (*Dnmt3* DKO), and *Dnmt* triple mutant (*Dnmt* TKO) E7.5 embryos. *C*, whole-genome DNA methylation was quantified for WT, *Dnmt1* mutant (*Dnmt1* KO), and *Dnmt3* double mutant (*Dnmt3* DKO) E8.5 embryos in comparison to the ICM of the WT blastocysts. Statistical analysis was carried out by using one-way ANOVA with Dunnett multiple comparison test. The values on the figures are shown as follows: ∗*p* < 0.05; ∗∗*p* < 0.01; ∗∗∗*p* < 0.001. ns, not statistically significant with *p*-value more than 0.1. DNMT, DNA methyltransferase; ICM, inner cell mass.

There were some published studies focused on DNA methylation in the preimplantation and postimplantation embryos (42, 43, 44, 45). DNMT proteins were examined for their functions in DNA methylation at E3.5 for the preimplantation stage, and at E6.5, E7.5, or E8.5 for the postimplantation stage. We analyzed the whole-genome bisulfite sequencing (WGBS) data in these published studies (42, 43, 44, 45). Interestingly, we found that about 20% of all CpG sites were methylated in the inner cell mass (ICM) of the E3.5 preimplantation embryos (Fig. 1) (44, 45). After implantation, DNA methylation increased to about 70% in the WT epiblast at E6.5 or E7.5 (Fig. 1, *A* and *B*). This increase in DNA methylation was abolished in the *Dnmt3* DKO mutant epiblasts at E6.5 or E7.5 lacking *Dnmt3a* and *Dnmt3b*, indicating that *de novo* DNA methylation accounted for the increased DNA methylation observed in the WT epiblasts (Fig. 1, *A* and *B*). The increased DNA methylation was largely lost in the *Dnmt1* KO mutant epiblasts at E6.5 or E7.5, consistent with the essential functions of DNMT1 in the maintenance DNA methylation in mouse embryos (Fig. 1, *A* and *B*). As expected, DNA methylation was almost completely lost in the *Dnmt* TKO mutant epiblasts at E6.5 or E7.5 lacking *Dnmt1*, *Dnmt3a*, and *Dnmt3b* (Fig. 1, *A* and *B*). However, there was still significant DNA methylation present in the epiblast of either *Dnmt1* KO or *Dnmt3* DKO mutant embryos at E6.5 and E7.5 (Fig. 1, *A* and *B*). Interestingly, there was significantly more DNA methylation in the *Dnmt1* KO mutant E6.5 epiblasts than in the WT ICM or *Dnmt3* DKO mutant E6.5 epiblasts, indicating that two DNMT3 proteins maintained newly acquired global DNA methylation in the epiblast of E6.5 embryos (Fig. 1*A*).

DNA methylation increased to over 80% in the WT E8.5 embryos (Fig. 1*C*). Nevertheless, the increased DNA methylation was also abolished in the *Dnmt3* DKO mutant E8.5 embryos and largely lost in the *Dnmt1* KO mutant E8.5 embryos (Fig. 1*C*).

In summary, it is *de novo* DNA methylation mediated by DNMT3A and DNMT3B that resulted in increase of DNA methylation after implantation. It is likely that DNMT1 maintained the preexisting DNA methylation at the global level, although we found DNMT3A and DNMT3B played a role in maintaining preexisting DNA methylation under some circumstances (see relevant heading). DNMT1 was involved in maintaining newly acquired DNA methylation, albeit not all, at the global level in the postimplantation embryos. DNMT3A and DNMT3B contributed to the maintenance of newly acquired DNA methylation across the genome as well as maintenance of preexisting DNA methylation at some genomic regions after implantation (see below).

### DNA methylation in repeats and retroviral elements in mouse embryos

Similar results were obtained when we analyzed the genomic regions containing the repeats and retroviral elements. Except for the low complexity regions that has low levels of DNA methylation (less than 5%), around 20% of the CpG sites were methylated in these repetitive sequences including DNA repeat, LINE, SINE, LTR, simple repeat and satellite repeat in the ICM of the WT E3.5 blastocysts (Fig. S1). After implantation, DNA methylation increased to about 80% or more in most repeats in the WT E6.5 or E7.5 epiblasts and WT E8.5 mouse embryos (Fig. S1). This increase in DNA methylation was also abolished in the E6.5 or E7.5 *Dnmt3* DKO mutant epiblasts or *Dnmt3* DKO mutant E8.5 embryos, and largely lost in the *Dnmt1* KO mutant epiblasts at E6.5 or E7.5 or *Dnmt1* KO mutant E8.5 embryos (Fig. S1). DNA methylation in the simple repeat regions was increased to about 60% in the WT E6.5 or E7.5 epiblasts, or more than 60% in the WT E8.5 mouse embryos (Fig. S1). This increase was also abolished in the *Dnmt3* DKO mutant E6.5-E7.5 epiblasts or mutant E8.5 embryos, and largely lost in the *Dnmt1* KO mutant epiblasts or embryos (Fig. S1). Similar observations were also obtained for the low complexity regions despite that there were low levels of DNA methylation (Fig. S1). Therefore, it is likely that *de novo* DNA methylation mediated by DNMT3A and DNMT3B account for the increased DNA methylation at the repeats after implantation, and DNMT1 is required for maintaining most of the newly acquired DNA methylation in the postimplantation embryos.

Generally, DNA methylation level present at the repeats in *Dnmt1* KO mutant E6.5-E7.5 epiblasts or E8.5 embryos was close to what was observed in the ICM of WT blastocysts (Fig. S1). By contrast, DNA methylation was completely missing in *Dnmt* TKO (Fig. S1). Interestingly, DNA methylation was significantly higher at the SINE repeats in the *Dnmt1* KO mutant E6.5-E7.5 epiblasts or *Dnmt1* KO mutant E8.5 embryos compared with the WT ICM samples, indicating that DNMT3A and DNMT3B maintain newly acquired DNA methylation at the SINE repeats after implantation (Fig. S1). It was also the case for DNA repeats in the *Dnmt1* KO mutant E6.5 epiblasts which displayed significantly higher DNA methylation than the WT ICM samples (Fig. S1). Thus, DNMT3A and DNMT3B, together with DNMT1, maintain DNA methylation at the repeats in the postimplantation embryos as lacking DNMT1 did not lead to complete loss of DNA methylation, and there is strong evidence that two DNMT3 proteins maintain newly acquired DNA at SINE and DNA repeats. The preexisting DNA methylation at the repeats is likely maintained by DNMT1 after implantation.

### DNA methylation in genic and intergenic regions in mouse embryos

**Figure 2:**
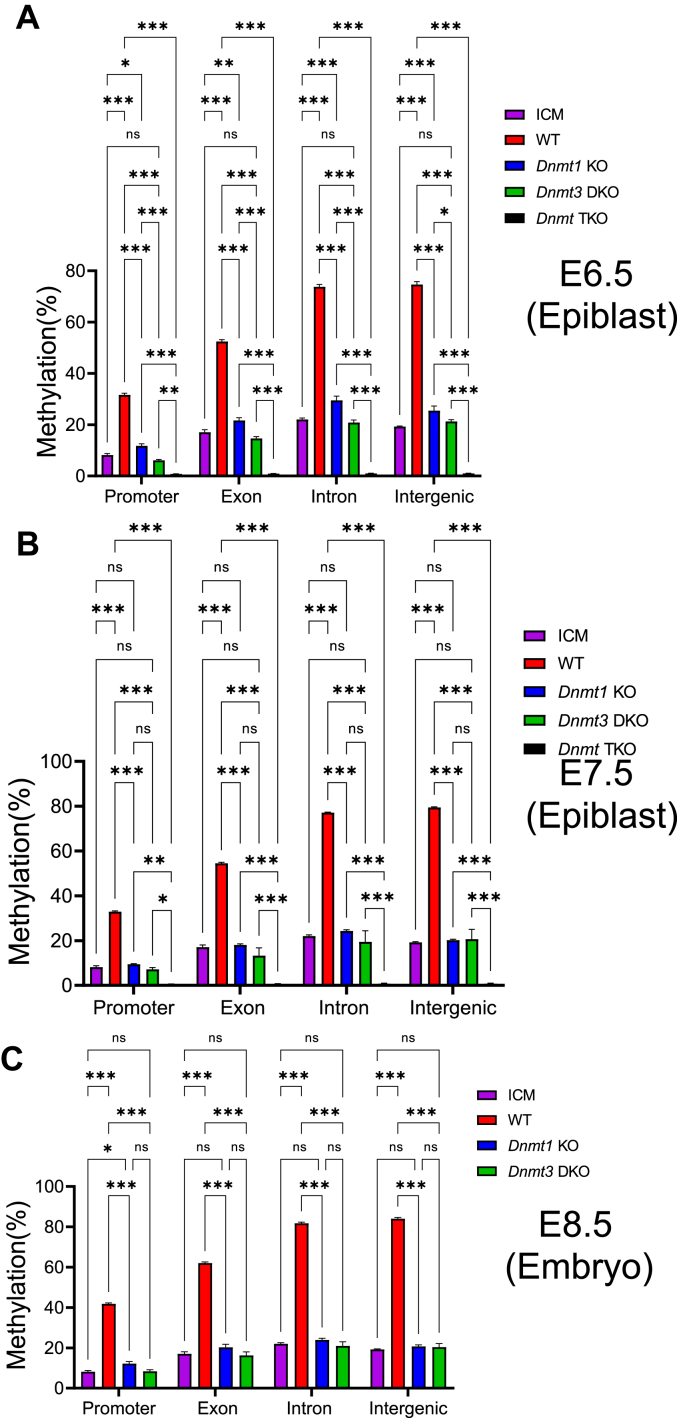
**Two DNMT3 proteins, together with DNMT1, maintained DNA methylation in the genic and intergenic regions after implantation**. DNA methylation was analyzed for the genic and intergenic regions in the ICM of the WT E3.5 blastocysts, the epiblasts of WT and *Dnmt* mutant E6.5-E7.5 embryos, as well as WT and *Dnmt* mutant E8.5 embryos. Horizontal axis, the genic and intergenic regions in the ICM at E3.5 or embryos at E6.5-E8.5. Vertical axis, % of DNA methylation. *A*, DNA methylation was analyzed for the WT ICM, the epiblasts of WT, *Dnmt1* mutant (*Dnmt1* KO), *Dnmt3* double mutant (*Dnmt3* DKO), and *Dnmt* triple mutant (*Dnmt* TKO) E6.5 embryos. *B*, DNA methylation was analyzed for the WT ICM, the epiblasts of WT, *Dnmt1* mutant (*Dnmt1* KO), *Dnmt3* double mutant (*Dnmt3* DKO) and *Dnmt* triple mutant (*Dnmt* TKO) E7.5 embryos. *C*, DNA methylation was examined at the genic and intergenic regions for WT, *Dnmt1* mutant (*Dnmt1* KO), and *Dnmt3* double mutant (*Dnmt3* DKO) E8.5 embryos in comparison to the WT ICM. Statistical analysis was carried out by using one-way ANOVA with Dunnett multiple comparison test. The values on the figures are shown as follows: ∗*p* < 0.05; ∗∗*p* < 0.01; ∗∗∗*p* < 0.001. ns, not statistically significant with *p*-value more than 0.1. DNMT, DNA methyltransferase; ICM, inner cell mass.

We also analyzed DNA methylation in the genic and intergenic regions in the preimplantation and postimplantation embryos. About 20% of the CpG sites were methylated in the genic regions containing the exon and introns as well as in the intergenic regions in the ICM of the WT E3.5 blastocysts (Fig. 2).

Less than 5% of the CpG sites were methylated in the promoter regions in the ICM of the WT E3.5 blastocysts (Fig. 2). DNA methylation increased to about 30% in the WT E6.5 or E7.5 epiblasts (Fig. 2, *A* and *B*). It increased to over 40% in the WT E8.5 embryos (Fig. 2*C*). This increase in DNA methylation was also abolished in the *Dnmt3* DKO mutant E6.5-E7.5 epiblasts or E8.5 embryos, and largely lost in the *Dnmt1* KO mutant epiblasts or mutant embryos (Fig. 2). DNA methylation increased to 50% or more in the exons of the WT E6.5 or E7.5 epiblasts (Fig. 2, *A* and *B*). It increased to over 60% in the WT E8.5 embryos (Fig. 2*C*). This increase was similarly abolished in the *Dnmt3* DKO mutant epiblasts or embryos, and largely lost in the *Dnmt1* KO mutant epiblasts or embryos (Fig. 2). DNA methylation increased to about 80% in the introns or intergenic regions of the WT E6.5 or E7.5 epiblasts (Fig. 2, *A* and *B*). It increased to more than 80% in the WT E8.5 embryos (Fig. 2*C*). The increased DNA methylation in the introns or intergenic regions was also absent in the *Dnmt3* DKO mutant epiblasts or embryos, and largely lost in the *Dnmt1* KO mutant epiblasts or embryos (Fig. 2). Interestingly, there was significantly more DNA methylation in these genic and intergenic regions in the *Dnmt1* KO mutant E6.5 epiblasts than in the WT ICM or *Dnmt3* DKO mutant E6.5 epiblasts, supporting the maintenance of newly acquired DNA methylation by two DNMT3 proteins in the genic and intergenic regions in the E6.5 epiblasts (Fig. 2*A*). This is consistent with their roles in maintaining newly acquired global DNA methylation in the epiblast of E6.5 embryos (Fig. 1*A*).

To examine DNA methylation at the intergenic regions in more detail, three separate intergenic regions analyzed in a previous study were subjected to DNA methylation analysis (Table S1) (46). Less than 20% of the CpG sites on two intergenic regions (intergenic 1 and intergenic 2) were methylated in the ICM of WT E3.5 blastocysts, whereas over 70% of the CpG sites on these two intergenic regions were methylated in the WT E6.5 or E7.5 epiblasts and more than 80% of these CpG sites were methylated in WT E8.5 embryos (Fig. S2). About 40% of the CpG sites on intergenic 3 were methylated in the ICM of WT E3.5 blastocysts, whereas over 80% of these CpG sites were methylated in the WT E6.5-E7.5 epiblasts and E8.5 embryos (Fig. S2). Interestingly, this increase in DNA methylation present in postimplantation embryos was abolished in the *Dnmt3* DKO mutant E6.5-E7.5 epiblasts and mutant E8.5 embryos because there was no significant difference in DNA methylation in these *Dnmt3* DKO mutant epiblasts and embryos compared with the ICM of WT E3.5 blastocysts (Fig. S2). As expected, DNA methylation was completely absent in the *Dnmt* TKO mutant E6.5-E7.5 epiblasts (Fig. S2, *A* and *B*). DNA methylation was significantly reduced on three intergenic regions in either *Dnmt1* KO or *Dnmt3* DKO mutant epiblasts and embryos in comparison to the ICM of WT E3.5 blastocysts (Fig. S2). However, significant levels of DNA methylation remained present on three intergenic regions in the *Dnmt1* KO and *Dnmt3* DKO mutant epiblasts compared with those in the *Dnmt* TKO mutant E6.5-E7.5 epiblasts, indicating that DNMT3A and DNMT3B, together with DNMT1, were necessary for maintaining DNA methylation on three intergenic regions (Fig. S2, *A* and *B*). Consistent with these, DNA methylation was partially lost on three intergenic regions in the *Dnmt1* KO or *Dnmt3* DKO mutant E8.5 embryos in comparison to the WT E8.5 embryos (Fig. S2*C*).

These observations were also confirmed when DNA methylation of the individual CpG sites is visualized at these intergenic regions (Figs. S3–S5). DNA methylation at the individual CpG sites of three intergenic regions was partially lost in the *Dnmt1* KO or *Dnmt3* DKO E6.5-E7.5 epiblasts, but completely lost in the *Dnmt* TKO mutant E6.5-E7.5 epiblasts (Figs. S3, *A* and *B*, S4, *A* and *B*, and S5, *A* and *B*). It was also partially lost in the *Dnmt1* KO or *Dnmt3* DKO mutant E8.5 embryos (Figs. S3*C*, S4*C*, and S5*C*).

Taken together, it is likely that *de novo* DNA methylation mediated by DNMT3A and DNMT3B accounts for the increased DNA methylation at the intergenic regions after implantation. DNMT1 is involved in maintaining most of the newly acquired DNA methylation in the postimplantation embryos, although DNMT3A and DNMT3B also function in maintaining a significant portion of newly acquired DNA methylation at the intergenic regions. Maintenance of the preexisting DNA methylation on the intergenic regions present in the ICM of blastocysts seems to be dependent on DNMT1 in the postimplantation embryos.

### DNA methylation in the CpG island regions

DNA methylation only occurred to about 3% of the CpG sites in the CpG island (CGI) regions in the ICM of WT blastocysts (Fig. S6). DNA methylation at the CGIs increased to about 4.5% in the epiblasts of WT E6.5-E7.5 embryos and increased to about 9% in WT E8.5 embryos, indicating continued gain of CGI methylation after implantation (Fig. S6, *A*–*C*). Partial loss of DNA methylation was observed in the *Dnmt1* KO or *Dnmt3* DKO mutant E6.5-E7.5 epiblasts in comparison to the WT E6.5-E7.5 epiblasts, whereas further loss (almost complete loss) of DNA methylation was observed in the *Dnmt* TKO mutant E6.5-E7.5 epiblasts (Fig. S6, *A* and *B*). DNA methylation was also partially lost at the CGIs in *Dnmt1* KO or *Dnmt3* DKO mutant E8.5 embryos compared with WT E8.5 embryos (Fig. S6*C*). Interestingly, DNA methylation at the CGIs was significantly lower in the *Dnmt1* KO or *Dnmt3* DKO mutant epiblasts compared with the ICM of WT E3.5 blastocysts, indicating maintenance of preexisting DNA methylation at the CGIs by two DNMT3 proteins as well as DNMT1 in the E6.5-E7.5 epiblasts. In contrast, it was significantly higher in the *Dnmt1* KO mutant E8.5 embryos and close to being significantly higher in the *Dnmt3* DKO mutant E8.5 embryos in comparison to the ICM of WT E3.5 blastocysts, indicating that DNMT3A and DNMT3B, together with DNMT1, maintained newly acquired DNA methylation at the CGIs in E8.5 embryos. Taken together, it is likely that *de novo* DNA methylation mediated by DNMT3A and DNMT3B accounts for small but noticeable increase of DNA methylation at the CGIs in the postimplantation embryos, which continues to increase after implantation. Two DNMT3 proteins, together with DNMT1, seem to be involved in maintaining preexisting as well as newly acquired DNA methylation at the CGIs after implantation.

### DNA methylation in the imprinted regions in mouse embryos

**Figure 3:**
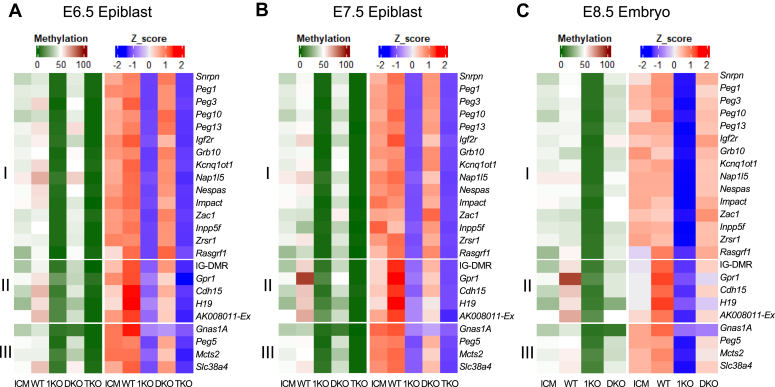
**DNA methylation was partially lost at a subset of imprinted regions in the postimplantation mutant embryos lacking DNMT3 proteins.** DNA methylation was analyzed for the imprinting control region (ICR) of 24 known imprinted regions in the ICM of WT E3.5 blastocysts, the epiblasts of WT and *Dnmt* mutant E6.5-E7.5 embryos, as well as WT and *Dnmt* mutant E8.5 embryos. DNA methylation was significantly reduced in 15 ICRs of group I in the *Dnmt1* KO, but not in *Dnmt3* DKO, mutant postimplantation embryos. There was significant increase in DNA methylation at 5 ICRs of group II after implantation comparing the epiblasts of WT E6.5-E7.5 embryos and WT E8.5 embryos with the ICM of WT E3.5 blastocysts. Group II includes the *AK008011-Ex* with the expanded ICR region found in this study (Fig. S9). Partial loss of DNA methylation was observed in four ICRs of group III in the *Dnmt3* DKO as well as in the *Dnmt1* KO mutant postimplantation embryos. *A*, DNA methylation was analyzed for the ICRs in the WT ICM, the epiblasts of WT, *Dnmt1* mutant (*Dnmt1* KO), *Dnmt3* double mutant (*Dnmt3* DKO), and *Dnmt* triple mutant (*Dnmt* TKO) E6.5 embryos. *B*, DNA methylation was analyzed for the ICRs in the WT ICM, the epiblasts of WT, *Dnmt1* mutant (*Dnmt1* KO), *Dnmt3* double mutant (*Dnmt3* DKO), and *Dnmt* triple mutant (*Dnmt* TKO) E7.5 embryos. *C*, DNA methylation was analyzed for the ICRs in the WT, *Dnmt1* mutant (*Dnmt1* KO), and *Dnmt3* double mutant (*Dnmt3* DKO) E8.5 embryos in comparison to the WT ICM. See the Methods details for DNA methylation and statistical analyses in this figure. DNMT, DNA methyltransferase; ICM, inner cell mass.

Currently, there are 24 known imprinted regions in mouse (Table S2) (40, 47). DNA methylation was analyzed for these ICRs in the ICM of the WT blastocysts with the data combined from two previously independently published studies (Table S3). We also examined DNA methylation at the ICRs of 24 imprinted regions in the *Dnmt1* KO mutant E6.5-E7.5 epiblasts or mutant E8.5 embryos in comparison to the WT E6.5-E7.5 epiblasts or WT E8.5 mouse embryos (Tables S4–S6) (Fig. 3). DNA methylation was drastically reduced in all tested ICRs in the *Dnmt1* KO mutant epiblasts or mutant E8.5 embryos (Fig. 3). As expected, DNA methylation was almost completely absent in all tested ICRs in the epiblasts of *Dnmt* TKO mutant E6.5-E7.5 embryos when DNMT1, DNMT3A, and DNMT3B were absent (Tables S4 and S5) (Fig. 3). Compared with the WT E6.5-E7.5 epiblasts or E8.5 embryos, there was negligible loss of DNA methylation at 15 ICRs in the *Dnmt3* DKO mutant E6.5-E7.5 epiblasts or mutant E8.5 embryos lacking DNMT3A and DNMT3B according to the Z score analysis (Tables S4–S6) (see 15 ICRs of group I in Fig. 3, *A*–*C*). Surprisingly, there was significant increase in DNA methylation at 5 ICRs of group II after implantation comparing the WT E6.5-E7.5 epiblasts and WT E8.5 embryos with the ICM of WT E3.5 blastocysts (Figs. 3, *A*–*C*, S8 and S9 below for *AK008011-Ex*). Furthermore, there was significant reduction in DNA methylation at these ICRs of group II in the *Dnmt3* DKO mutant E6.5-E7.5 epiblasts or mutant E8.5 embryos compared with the WT E6.5-E7.5 epiblasts or E8.5 embryos, although DNA methylation was more dramatically reduced in the *Dnmt1* KO mutant E6.5-E7.5 epiblasts or mutant E8.5 embryos (Fig. 3, *A*–*C*). Intriguingly, partial loss of DNA methylation was observed in 4 ICRs of group III in the *Dnmt3* DKO mutant E6.5-E7.5 epiblasts or mutant E8.5 embryos in comparison to the corresponding WT samples at the same stages according to the Z score analysis results (Fig. 3, *A*–*C*).

Taken together, these results suggest that DNMT1 is the major DNMT necessary for the maintenance of DNA methylation of all ICRs in mouse embryos whereas DNMT3A and DNMT3B may contribute to the maintenance of DNA methylation at a large subset of ICRs in postimplantation embryos. These will be discussed further in the following sections according to their distinctive functions at the ICRs.

### Maintenance of DNA methylation by DNMT1 at 15 imprinted regions in mouse embryos

**Figure 4:**
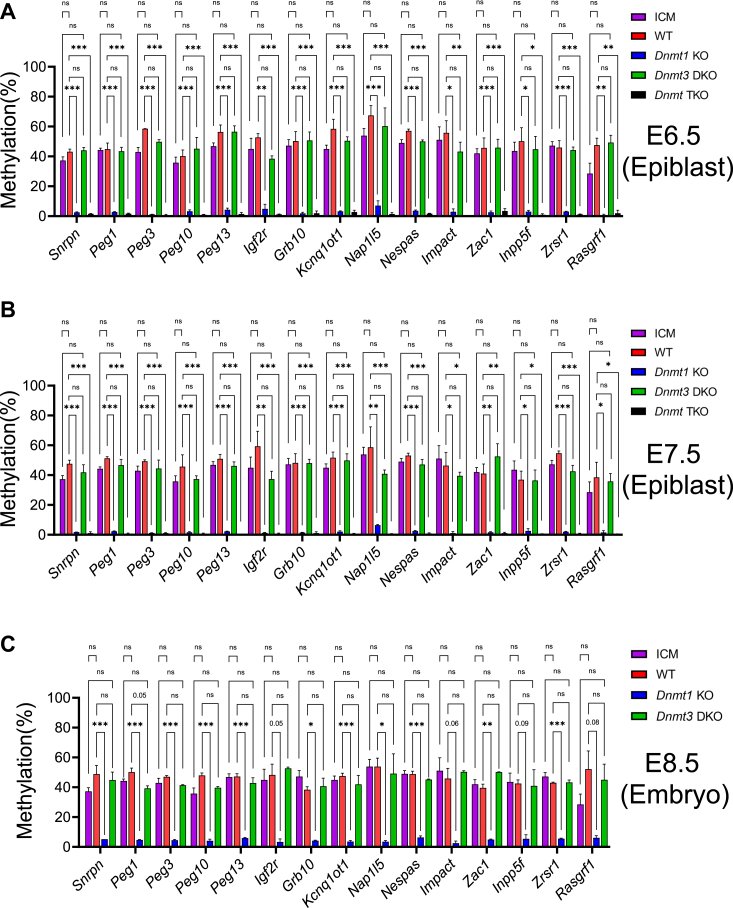
**DNMT1 maintained DNA methylation at most imprinted regions in the postimplantation embryos**. DNA methylation was analyzed for the imprinting control region (ICR) of the known imprinted regions in the ICM of the WT E3.5 blastocysts, the epiblasts of WT and *Dnmt* mutant E6.5-E7.5 embryos, as well as WT and *Dnmt* mutant E8.5 embryos. DNA methylation was significantly reduced in these ICRs in the *Dnmt1* KO, but not in *Dnmt3* DKO, mutant postimplantation embryos. *A*, DNA methylation was analyzed for these ICRs in the WT ICM, the epiblasts of WT, *Dnmt1* mutant (*Dnmt1* KO), *Dnmt3* double mutant (*Dnmt3* DKO), and *Dnmt* triple mutant (*Dnmt* TKO) E6.5 embryos. *B*, DNA methylation was analyzed for these ICRs in the WT ICM, the epiblasts of WT, *Dnmt1* mutant (*Dnmt1* KO), *Dnmt3* double mutant (*Dnmt3* DKO), and *Dnmt* triple mutant (*Dnmt* TKO) E7.5 embryos. *C*, DNA methylation was analyzed for these ICRs in the WT, *Dnmt1* mutant (*Dnmt1* KO), and *Dnmt3* double mutant (*Dnmt3* DKO) E8.5 embryos in comparison to the WT ICM. Statistical analysis was carried out by using one-way ANOVA with Dunnett multiple comparison test. Besides a few numbers for the real values, these symbols represent the following values in statistical analysis on the figure: ∗*p* < 0.05; ∗∗*p* < 0.01; ∗∗∗*p* < 0.001. ns, not statistically significant with *p*-value more than 0.1. DNMT, DNA methyltransferase; ICM, inner cell mass.

We examined DNA methylation in the imprinted regions in the preimplantation and postimplantation embryos. There was no significant difference in DNA methylation level at 15 ICRs in the WT E6.5-E7.5 epiblasts or WT E8.5 embryos compared with the ICM of WT E3.5 blastocysts (Fig. 4, *A*–*C*). DNA methylation was almost absent at these 15 ICRs that are the same ICRs of group I in Figure 3, in either *Dnmt1* KO mutant E6.5-E7.5 epiblasts and mutant E8.5 embryos, or in the *Dnmt* TKO mutant E6.5-E7.5 epiblasts (Figs. 3 and 4, *A*–*C*). Except for the *Peg1* ICR that exhibited close to significantly lower DNA methylation in the *Dnmt3* DKO mutant E8.5 embryos in comparison to the WT E8.5 embryos, similar levels of DNA methylation were observed at these 15 ICRs in the *Dnmt3* DKO mutant E6.5-E7.5 epiblasts or mutant E8.5 embryos in comparison to the WT epiblasts or embryos at the same stages (Fig. 4, *A*–*C*).

Based on these results, we can make a few assumptions. It appears that germline-derived differential DNA methylation is relatively stable at 15 ICRs in the preimplantation and postimplantation embryos. Preexisting DNA methylation at these 15 ICRs is almost exclusively maintained by DNMT1 in mouse embryos. DNMT3A and DNMT3B do not seem to play any noticeable role in the maintenance of DNA methylation at these 15 ICRs.

### DNMT3A and DNMT3B function in *de novo* and maintenance DNA methylation in a subset of imprinted regions in postimplantation embryos

**Figure 5:**
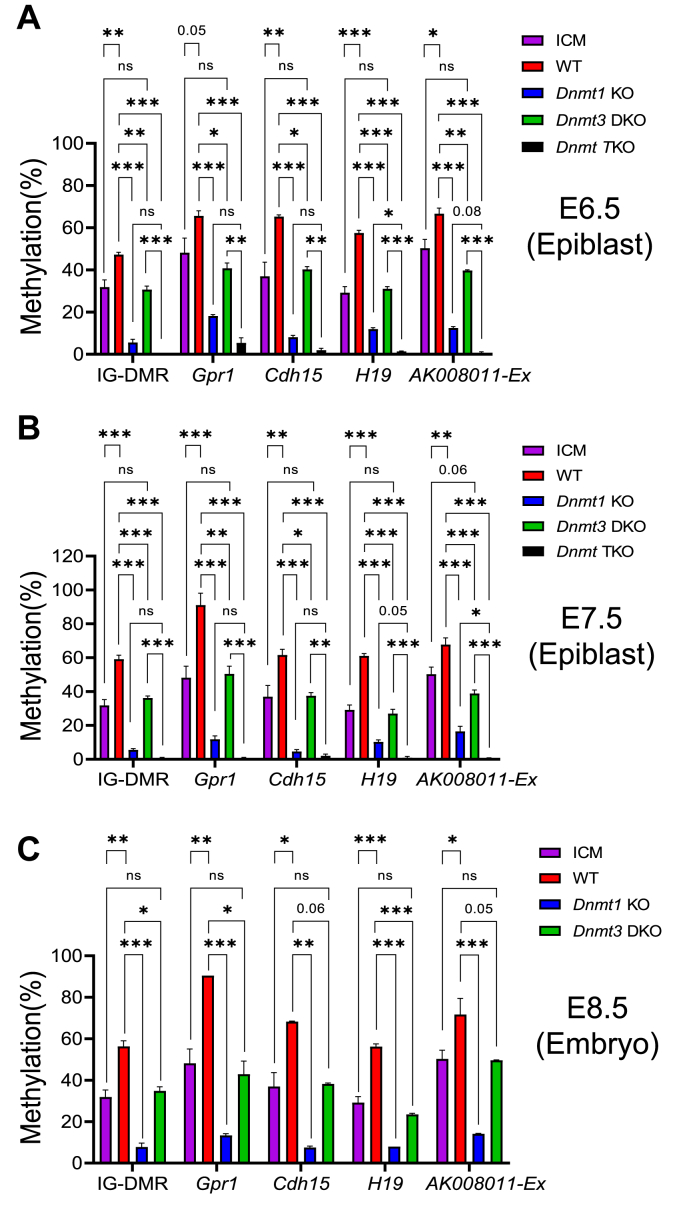
**DNMT3A and DNMT3B were required for increased DNA methylation as well as maintenance DNA methylation at five imprinted regions after implantation**. DNA methylation was analyzed for the imprinting control region (ICR) of the imprinted regions in the ICM of the WT E3.5 blastocysts, the epiblasts of WT and *Dnmt* mutant E6.5-E7.5 embryos, as well as WT and *Dnmt* mutant E8.5 embryos. DNA methylation was significantly reduced in the IG-DMR of the *Dlk1-Dio3*, *Gpr1*, *Cdh15*, *H19*, and *AK008011-Ex* ICRs of the *Dnmt3* DKO mutant postimplantation embryos (Fig. S9 for *AK008011-Ex*). *A*, DNA methylation was analyzed for these five ICRs in the WT ICM, the epiblasts of WT, *Dnmt1* mutant (*Dnmt1* KO), *Dnmt3* double mutant (*Dnmt3* DKO), and *Dnmt* triple mutant (*Dnmt* TKO) E6.5 embryos. *B*, DNA methylation was analyzed for these five ICRs in the WT ICM, the epiblasts of WT, *Dnmt1* mutant (*Dnmt1* KO), *Dnmt3* double mutant (*Dnmt3* DKO), and *Dnmt* triple mutant (*Dnmt* TKO) E7.5 embryos. *C*, DNA methylation was analyzed for these five ICRs in the WT, *Dnmt1* mutant (*Dnmt1* KO), and *Dnmt3* double mutant (*Dnmt3* DKO) E8.5 embryos in comparison to the WT ICM. Statistical analysis was performed by using one-way ANOVA with Dunnett multiple comparison test. In addition to several numbers for the *p*-value, the significance in statistical analysis is shown with the symbols representing the following values: ∗*p* < 0.05; ∗∗*p* < 0.01; ∗∗∗*p* < 0.001. ns, not statistically significant with *p*-value more than 0.1. DNMT, DNA methyltransferase; ICM, inner cell mass.

Compared with the ICM of E3.5 blastocysts, there was an increase in DNA methylation at the ICRs (IG-DMR, *Gpr1*, *Cdh15*, and *H19*) of four imprinted regions in the WT E6.5-E7.5 epiblasts or WT E8.5 embryos (Fig. 5). The increased DNA methylation at these four ICRs was completely abolished in the *Dnmt3* DKO mutant E6.5-E7.5 epiblasts or *Dnmt3* DKO mutant E8.5 embryos as there was no significant difference in DNA methylation level at four ICRs comparing these *Dnmt3* DKO mutant samples with the WT ICM samples, indicating that this increase was due to *de novo* DNA methylation mediated by DNMT3A and DNMT3B (Fig. 5). By contrast, DNA methylation was significantly reduced at these four ICRs in the *Dnmt3* DKO mutant E6.5-E7.5 epiblasts or mutant E8.5 embryos in comparison with the corresponding WT E6.5-E7.5 epiblasts or E8.5 embryos (Fig. 5). As expected, DNA methylation was almost completely eliminated at four ICRs in the epiblasts of *Dnmt* TKO mutant E6.5-E7.5 embryos (Fig. 5, *A* and *B*). There appeared to be some residual DNA methylation at these ICRs in the *Dnmt1* KO mutant E6.5-E7.5 epiblasts and mutant E8.5 embryos (Fig. 5).

These observations were confirmed by the DNA methylation IGV plots at these four ICRs in the germ cells, preimplantation blastocysts and postimplantation embryos (Fig. S7) (https://igv.org/doc/desktop/#DownloadPage/). As expected, higher levels of DNA methylation were present at the IG-DMR and *H19* ICRs in the WT sperm samples compared with the WT oocyte samples, whereas methylation was only observed at the *Gpr1* and *Cdh15* ICRs in the oocytes but not in the sperm (Fig. S7). According to the IGV plots, DNA methylation was almost completely missing in all CpG sites at these four ICRs in the *Dnmt* TKO mutant E6.5-E7.5 epiblasts (Fig. S7, *A* and *B*). DNA methylation was partially lost in almost all CpG sites at these four ICRs in either *Dnmt1* KO mutant epiblasts or *Dnmt3* DKO mutant epiblasts, in comparison to the WT epiblasts (Fig. S7, *A* and *B*). Similar findings were also observed in the *Dnmt1* KO or *Dnmt3* DKO mutant E8.5 embryos, compared with the WT E8.5 embryos (Fig. S7*C*).

As shown in the IGV plots, DNA methylation at the *AK008011* ICR was only present in the oocytes but not in the sperm as it is known to be established during oogenesis (Fig. S8). However, differential DNA methylation in this imprinted region appeared to be in a much broader window than what was defined in the original studies (compare Fig. S8, *A*’–*C*’ with Fig. S8, *A*–*C*). There was no significant difference in DNA methylation at the previously identified *AK008011* ICR in the WT E6.5-E7.5 epiblasts or WT E8.5 embryos, compared with the ICM of blastocysts when the original *AK008011* ICR window was utilized in DNA methylation analysis (Figs. S8, *A*–*C* and S9, *A*–*C*). By contrast, DNA methylation levels were significantly increased at the newly defined *AK008011-Ex* ICR in the WT E6.5-E7.5 epiblasts and WT E8.5 embryos in comparison to the WT ICM when DNA methylation was measured for the broader *AK008011-Ex* ICR window identified in this study (Figs. S8, *A*’–*C*’ and S9, *A*’–*C*’). This increase in DNA methylation at the *AK008011-Ex* ICR appeared to be dependent on DNMT3A and DNMT3B since it was abolished in the *Dnmt3* DKO mutant E6.5-E7.5 epiblasts or *Dnmt3* DKO mutant E8.5 embryos (Fig. S9, *A*’–*C*’). Partial loss of DNA methylation was also observed in the *Dnmt3* DKO mutant E6.5-E7.5 epiblasts or *Dnmt3* DKO mutant E8.5 embryos, compared with the WT E6.5-E7.5 epiblasts and WT E8.5 embryos at the same stage when this broad window of *AK008011-Ex* ICR was used in DNA methylation analysis (Fig. S9, *A*’–*C*’). However, there was no significant loss of DNA methylation in the *Dnmt3* DKO mutant E6.5-E7.5 epiblasts or the *Dnmt3* DKO mutant E8.5 embryos compared with the WT ones at the same stage when DNA methylation was measured for the originally defined *AK008011* ICR (Fig. S9, *A*–*C*). Accordingly, we believe that the *AK008011* ICR may encompass a broader region than originally defined, which was named as *AK008011-Ex* that showed increased DNA methylation after implantation. This increase in DNA methylation required DNMT3A and DNMT3B.

There was some residual DNA methylation at the IG-DMR, *Gpr1*, *Cdh15, H19*, and *AK008011-Ex* ICRs in the *Dnmt1* KO mutant E6.5-E7.5 epiblasts and mutant E8.5 embryos (Fig. 5). Interestingly, DNA methylation was significantly higher (or close to being) at the *H19* and *AK008011-Ex* ICRs in the *Dnmt1* KO mutant E6.5-E7.5 epiblasts in comparison to the *Dnmt* TKO mutant E6.5-E7.5 epiblasts (Fig. 5, *A* and *B*). Accordingly, these results indicate that DNMT3A and DNMT3B maintain newly acquired DNA methylation at these two ICRs after implantation.

Taken together, it is likely that DNMT1 maintains the preexisting DNA methylation at five ICRs (IG-DMR, *Gpr1*, *Cdh15, H19*, and *AK008011-Ex*) after implantation. DNMT3A and DNMT3B exhibit *de novo* DNA methylation at these five ICRs after implantation so that there is increased DNA methylation in the postimplantation embryos in comparison with the ICM of the blastocysts. Together with DNMT1, they maintain newly acquired DNA methylation at two of these ICRs in the postimplantation embryos.

### DNMT3A and DNMT3B, together with DNMT1, maintain DNA methylation in four imprinted regions in mouse embryos

**Figure 6:**
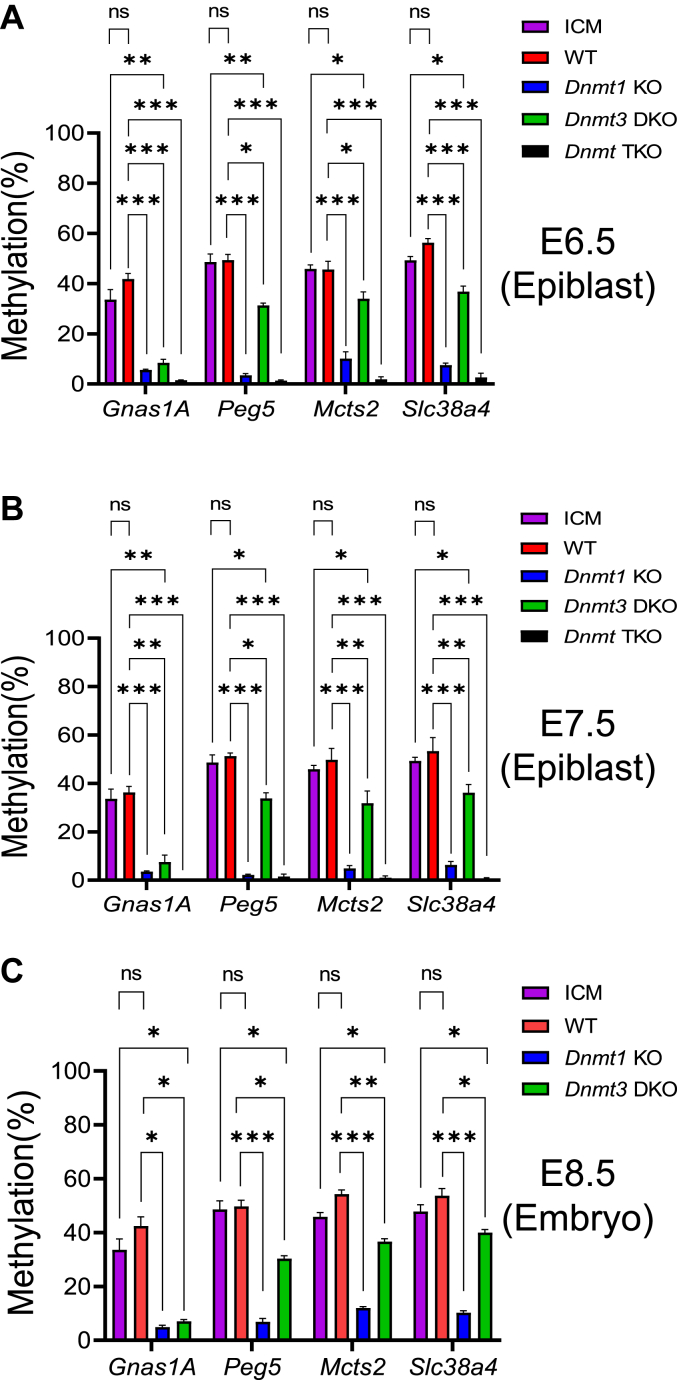
**DNMT3 proteins maintained preexisting germline-derived DNA methylation at a subset of imprinted regions in the postimplantation embryos**. DNA methylation was analyzed for the imprinting control region (ICR) of the imprinted regions in the ICM of the WT E3.5 blastocysts, the epiblasts of WT and *Dnmt* mutant E6.5-E7.5 embryos, as well as WT and *Dnmt* mutant E8.5 embryos. DNA methylation was significantly reduced in the *Gnas1A*, *Peg5*, *Mcts2*, and *Slc38a4* ICRs of the *Dnmt3* DKO mutant postimplantation embryos in comparison to the WT ICM or WT embryos at E6.5-E8.5. *A*, DNA methylation was analyzed for these four ICRs in the WT ICM, the epiblasts of WT, *Dnmt1* mutant (*Dnmt1* KO), *Dnmt3* double mutant (*Dnmt3* DKO), and *Dnmt* triple mutant (*Dnmt* TKO) E6.5 embryos. *B*, DNA methylation was analyzed for these four ICRs in the WT ICM, the epiblasts of WT, *Dnmt1* mutant (*Dnmt1* KO), *Dnmt3* double mutant (*Dnmt3* DKO), and *Dnmt* triple mutant (*Dnmt* TKO) E7.5 embryos. *C*, DNA methylation was analyzed for these four ICRs in the WT, *Dnmt1* mutant (*Dnmt1* KO), and *Dnmt3* double mutant (*Dnmt3* DKO) E8.5 embryos in comparison to the WT ICM. One-way ANOVA with Dunnett multiple comparison test was used for statistical analyses, with the values shown on the figure as follows: ∗*p* < 0.05; ∗∗*p* < 0.01; ∗∗∗*p* < 0.001. ns, not statistically significant with *p*-value more than 0.1. DNMT, DNA methyltransferase; ICM, inner cell mass.

There was no significant difference in DNA methylation levels at the *Gnas1A*, *Peg5*, *Mcts2*, and *Slc38a4* ICRs comparing the ICM of WT E3.5 blastocysts with the WT E6.5-E7.5 epiblasts or WT E8.5 embryos, suggesting *de novo* DNA methylation did not occur at these ICRs after implantation (Fig. 6). As expected, DNA methylation was completely absent at four ICRs in the *Dnmt* TKO mutant epiblasts (Fig. 6, *A* and *B*). Interestingly, DNA methylation was significantly reduced at these four ICRs in the *Dnmt3* DKO mutant E6.5-E7.5 epiblasts and *Dnmt3* DKO mutant E8.5 embryos, in comparison to the WT E6.5-E7.5 epiblasts and WT E8.5 embryos (Fig. 6). Consistent with these results, residual DNA methylation was present at these four ICRs in the *Dnmt1* KO mutant epiblasts and *Dnmt1* KO mutant E8.5 embryos (Fig. 6). These data indicate that DNMT3A and DNMT3B, together with DNMT1, maintain DNA methylation at the *Gnas1A*, *Peg5*, *Mcts2*, and *Slc38a4* ICRs in the postimplantation embryos.

Loss of DNA methylation at the ICRs can also be visualized by the DNA methylation IGV plots at these four ICRs in the germ cells, preimplantation blastocysts and postimplantation embryos (Fig. S10). Since DNA methylation at these four ICRs is known to be established on the maternal chromosome during oogenesis, DNA methylation was only observed at four ICRs in the oocytes but not in the sperm (Fig. S10). As shown on the IGV plots, DNA methylation was almost absent in all CpG sites at four ICRs in the epiblasts of *Dnmt* TKO mutant E6.5-E7.5 embryos (Fig. S10, *A* and *B*). By contrast, DNA methylation was partially lost in almost all CpG sites at these four ICRs in the *Dnmt1* KO or *Dnmt3* DKO mutant E6.5-E7.5 epiblasts, compared with the WT E6.5-E7.5 epiblasts (Fig. S10, *A* and *B*). These were also observed in the *Dnmt1* KO or *Dnmt3* DKO mutant E8.5 embryos, in comparison with the WT E8.5 embryos (Fig. S10*C*).

In summary, DNMT3A and DNMT3B, together with DNMT1, appeared to maintain preexisting DNA methylation at four ICRs (*Gnas1A*, *Peg5*, *Mcts2*, and *Slc38a4* ICRs) in the postimplantation embryos.

### DNA methylation at the pluripotent genes

Some pluripotent genes including *Pou5f1* (*Oct4*) and *Nanog* are highly expressed in the ICM of blastocysts because DNA methylation is almost absent at these genes in the ICM (Fig. S11). As expected, *de novo* DNA methylation occurred at *Pou5f1* and *Nanog* after implantation (Table S7 and Fig. S11). More than 40% CpG sites were found to be methylated in the *Pou5f1* gene in the epiblast of WT E6.5 or E7.5 embryos, whereas over 60% CpG sites at the *Nanog* gene were methylated in the epiblast of WT E6.5 or E7.5 embryos (Fig. S11, *A* and *B*). Approximately 40% of the CpG sites were methylated at the *Pou5f1* gene in the WT E8.5 embryos, whereas over 60% CpG sites at the *Nanog* gene were methylated in the WT E8.5 embryos (Fig. S11*C*). The increased DNA methylation was abolished at *Pou5f1* and *Nanog* in the *Dnmt3* DKO mutant E6.5-E7.5 epiblasts and mutant E8.5 embryos (Fig. S11), indicating *de novo* methylation mediated by two DNMT3 proteins was responsible for the increase in DNA methylation at *Pou5f1* and *Nanog* in the postimplantation embryos.

DNA methylation was completely absent at *Pou5f1* and *Nanog* in the *Dnmt* TKO mutant E6.5-E7.5 epiblasts (Fig. S11, *A* and *B*). However, residual DNA methylation was still observed in either *Dnmt1* KO or *Dnmt3* DKO mutant E6.5-E7.5 epiblasts. In particular, over 20% CpG sites were methylated at the *Nanog* gene in the *Dnmt1* KO mutant E6.5-E7.5 epiblasts, which was significantly higher than what was observed in the *Dnmt* TKO mutant E6.5-E7.5 epiblasts or *Dnmt3* DKO mutant E6.5-E7.5 epiblasts or WT ICM (Fig. S11, *A* and *B*). Similarly, DNA methylation at both *Pou5f1* and *Nanog* was also significantly higher in the *Dnmt1* KO mutant E8.5 embryos than in the *Dnmt3* DKO mutant E8.5 embryos (Fig. S11*C*). These results suggest that two DNMT3 proteins, together with DNMT1, maintain newly acquired DNA methylation at both *Pou5f1* and *Nanog* in the postimplantation embryos, which is more conspicuous at *Nanog*.

Similar results were also observed at *Pou5f1* and *Nanog* when DNA methylation at the individual CpG sites was visualized on the IGV plots of these two genes (Figs. S12 and S13).

### DNA methylation at the genes of three germ layers in mouse development

It is known that expression of some genes critical for mouse embryonic development may be regulated by DNA methylation upon specification of three germ layers after implantation. Therefore, we examined DNA methylation on two representative marker genes in each germ layer, with *Brachyury* (*T*) and *Troponin T2* (*Tnnt2*) for mesoderm, *Nestin* and *Tyrosine hydroxylase* (*Th*) for ectoderm, and *Gata6* and *Alpha fetoprotein* (*Afp*) for endoderm.

To find out how they may be regulated during mesoderm development, we analyzed DNA methylation at the *Brachyury* (*T*) and *Tnnt2* genes in the ICM of blastocyst and the epiblast of E6.5-E7.5 embryos (Table S7 and Fig. S14, *A* and *B*). There was little DNA methylation at *T* and *Tnnt2* in the ICM of WT blastocyst (Fig. S14, *A* and *B*). Approximately 40% and 70% of the CpG sites were methylated at *T* and *Tnnt2*, respectively, in the epiblast of WT E6.5-E7.5 embryos (Fig. S14, *A* and *B*). More than 40% and 70% of the CpG sites were methylated at *T* and *Tnnt2*, respectively, in the WT E8.5 embryos (Fig. S14*C*). This increase of DNA methylation was abolished at *T* and *Tnnt2* in the E6.5-E7.5 *Dnmt3* DKO mutant epiblasts or *Dnmt3* DKO mutant E8.5 embryos (Fig. S14). It is likely that *de novo* methylation mediated by DNMT3A and DNMT3B is responsible for the increase in DNA methylation at *T* and *Tnnt2* after implantation.

DNA methylation was completely absent at *T* and *Tnnt2* in the *Dnmt* TKO mutant E6.5-E7.5 epiblasts, whereas residual DNA methylation was still observed in either *Dnmt1* KO or *Dnmt3* DKO mutant E6.5-E7.5 epiblasts (Fig. S14, *A* and *B*). Interestingly, DNA methylation was significantly higher at *T* and *Tnnt2* in the *Dnmt1* KO mutant E6.5-E7.5 epiblasts than in the *Dnmt3* DKO mutant E6.5-E7.5 epiblasts or WT ICM. Similarly, DNA methylation was significantly higher at *T* and *Tnnt2* in the *Dnmt1* KO mutant E8.5 embryos than in the *Dnmt3* DKO mutant E8.5 embryos or WT ICM (Fig. S14*C*). These results prove that two DNMT3 proteins, together with DNMT1, maintain newly acquired DNA methylation at two mesodermal marker genes that is gained after implantation.

Similar results are obtained at *T* and *Tnnt2* when DNA methylation at the individual CpG sites are examined on the IGV plots of these two genes (Figs. S15 and S16). Taken together, these results suggest that two DNMT3 proteins, together with DNMT1, are involved in the maintenance of newly acquired DNA methylation at *T* and *Tnnt2* in the postimplantation embryos.

To analyze the functions of DNA methylation in ectoderm development, we examined DNA methylation at the *Nestin* and *Th* genes in the ICM of WT blastocysts as well as in the E6.5-E7.5 epiblasts and E8.5 embryos (Table S7 and Fig. S17, *A* and *B*). There was little DNA methylation at *Nestin* in the ICM of WT blastocysts, whereas around 30% of the CpG sites were methylated at *Th* in the ICM of WT blastocysts (Fig. S17). Approximately 20% and 70% of the CpG sites were methylated at *Nestin* and *Th*, respectively, in the WT E6.5-E7.5 epiblasts (Fig. S14, *A* and *B*). More than 30% and 70% of the CpG sites were methylated at *Nestin* and *Th*, respectively, in the WT E8.5 embryos (Fig. S17*C*). This increase of DNA methylation was abolished at *Nestin* and *Th* in the *Dnmt3* DKO mutant E6.5-E7.5 epiblasts or *Dnmt3* DKO mutant E8.5 embryos (Fig. S17). Therefore, it is likely that *de novo* methylation mediated by DNMT3A and DNMT3B results in increase of DNA methylation at *Nestin* and *Th* after implantation.

DNA methylation was completely absent at *Nestin* and *Th* in the *Dnmt* TKO mutant E6.5-E7.5 epiblasts (Fig. S17, *A* and *B*). In contrast, residual DNA methylation was still observed at *Nestin* in the *Dnmt1* KO mutant epiblasts, albeit not in the *Dnmt3 DKO* mutant E6.5-E7.5 epiblasts, and at *Th* in either *Dnmt1* KO or *Dnmt3* DKO mutant E6.5-E7.5 epiblasts (Fig. S17, *A* and *B*). These results suggest that DNMT3A and DNMT3B, together with DNMT1, are required for maintaining DNA methylation at these two ectodermal marker genes. Interestingly, DNA methylation was significantly higher at *Nestin* in the *Dnmt1* KO mutant E6.5-E7.5 epiblasts or *Dnmt1* KO mutant E8.5 embryos than in that of the corresponding *Dnmt3* DKO mutant samples (Fig. S17). Thus, this is the further proof that two DNMT3 proteins are necessary for maintaining newly acquired DNA methylation at *Nestin* after implantation. Surprisingly, DNA methylation was close to be significantly reduced at *Th* in the *Dnmt3* DKO mutant E7.5 epiblasts and *Dnmt3* DKO mutant E8.5 embryos compared with the ICM of WT blastocysts (Fig. S17, *B* and *C*). Therefore, two DNMT3 proteins probably play a role in maintaining the preexisting DNA methylation at *Th* in the postimplantation embryos.

Similar results are obtained at *Nestin* and *Th* when DNA methylation at the individual CpG sites are examined on the IGV plots of these two genes (Figs. S18 and S19). In summary, two DNMT3 proteins, together with DNMT1, maintain DNA methylation at two ectodermal marker genes *Nestin* and *Th* in the postimplantation embryos. Indeed, two DNMT3 proteins are required for maintaining newly acquired DNA methylation at *Nestin* that is gained after implantation, as is the likely to be the case at *Th* as well. The preexisting DNA methylation at *Th* that is present in the ICM is probably maintained by two DNMT3 proteins as well as DNMT1 in the postimplantation embryos.

We also examined DNA methylation at the *Gata6* and *Afp* genes, two genes involved in endoderm development (Table S7 and Fig. S20). There was little DNA methylation at *Gata6* in the ICM of WT blastocyst, whereas approximately 40% of the CpG sites were methylated at *Afp* in the ICM of blastocysts (Fig. S20). Over 30% of the CpG sites were methylated at *Gata6* in the epiblasts of WT E6.5-E7.5 embryos while close to 80% of the CpG sites at *Afp* were methylated in the epiblasts of WT E6.5-E7.5 embryos (Fig. S20, *A* and *B*). Over 40% and 80% of the CpG sites were methylated at *Gata6* and *Afp*, respectively, in the WT E8.5 embryos (Fig. S20*C*). This increase of DNA methylation at *Gata6* and *Afp* was abolished in the *Dnmt3* DKO mutant E6.5-E7.5 epiblasts and *Dnmt3* DKO mutant E8.5 (Fig. S20).

DNA methylation was completely absent at *Gata6* and *Afp* in the *Dnmt* TKO mutant E6.5-E7.5 epiblasts (Fig. S20, *A* and *B*). By contrast, noticeable amount (about 5–10%) of DNA methylation was still observed at *Gata6* in the *Dnmt1* KO mutant E6.5-E7.5 epiblasts or mutant E8.5 embryos (Fig. S20, *A*–*C*). And there were still over 20% of DNA methylation at *Afp* in either *Dnmt1* KO and *Dnmt3* DKO mutant E6.5-E7.5 epiblasts or *Dnmt1* KO and *Dnmt3* DKO mutant E8.5 embryos (Fig. S20, *A*–*C*). Interestingly, DNA methylation was significantly higher at *Gata6* in the *Dnmt1* KO mutant E6.5-E7.5 epiblasts or *Dnmt1* KO mutant E8.5 embryos than in the corresponding *Dnmt3* DKO mutant or WT ICM samples (Fig. S20). These results prove that two DNMT3 proteins contribute to the maintenance of newly acquired DNA methylation at *Gata6* after implantation. It is also likely true that two DNMT3 proteins maintain newly acquired DNA methylation at *Afp* after implantation since DNMT1 seems to be solely responsible for the maintenance of the preexisting DNA methylation at *Afp* in the postimplantation embryos.

Similar results are obtained at *Gata6* and *Afp* when DNA methylation at the individual CpG sites are examined on the IGV plots of these two genes (Figs. S21 and S22). To summarize, two DNMT3 proteins, together with DNMT1, contribute to the maintenance of newly acquired DNA methylation at *Gata6* and likely at *Afp* as well after implantation in endodermal development.

Taken together, we conclude that two DNMT3 proteins maintain newly acquired DNA methylation after implantation at some representative marker genes in three germ layers that we have examined in this study. Furthermore, two DNMT3 proteins, together with DNMT1, may be also required for maintaining the preexisting DNA methylation present on certain marker genes (*e.g. Th*) in the postimplantation embryos.

## Discussion

**Figure 7:**
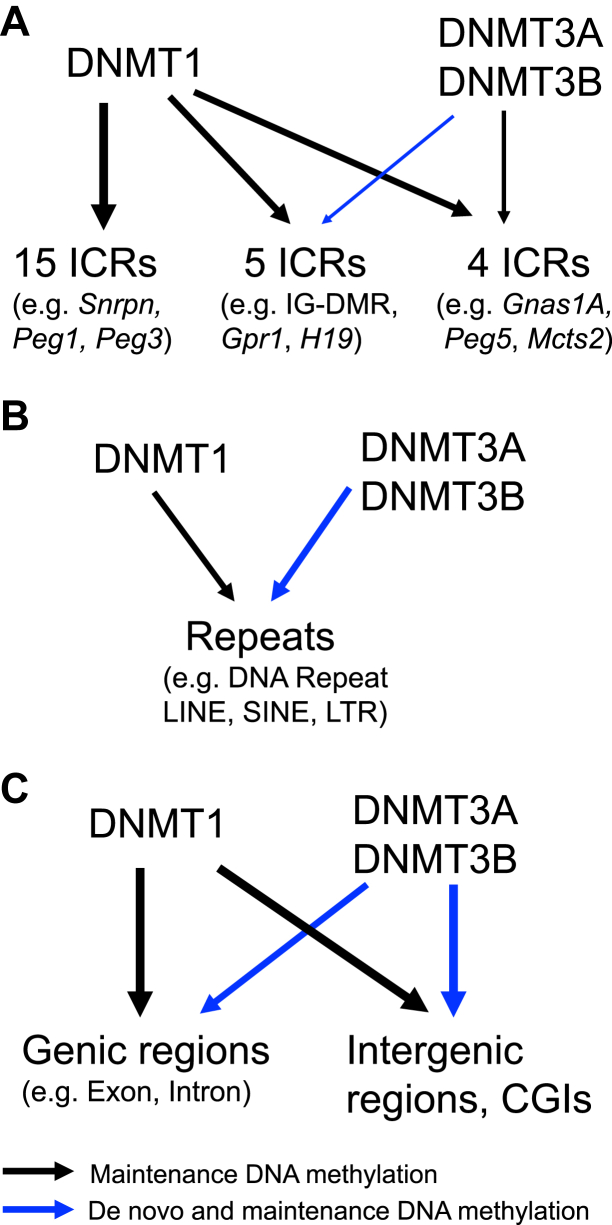
**The schematic diagrams show the functions of three DNMT proteins in DNA methylation in the embryos after implantation.** Two DNMT3 proteins, together with DNMT1, may maintain DNA methylation in the ICRs, repeats, genic, intergenic or CpG island (CGI) regions after implantation. Besides maintenance DNA methylation, DNMT3A and DNMT3B may also function in *de novo* DNA methylation of these genomic regions after implantation. The line thickness of the arrows are in proportion to their hypothetical roles in DNA methylation. *A*, DNA methylation at the ICRs in the postimplantation embryos. DNMT1 maintains preexisting DNA methylation at 15 ICRs such as *Snrpn*, *Peg1*, and *Peg3*. Interestingly, DNMT3A and DNMT3B display *de novo* DNA methylation at five other ICRs including IG-DMR, *Gpr1*, *Cdh15*, *H19*, and *AK008011-Ex* that was shown to probably harbor a broader genomic region in this study. There is strong evidence that DNMT3A and DNMT3B, together with DNMT1, maintain newly acquired DNA methylation at the *H19* and *AK008011-Ex* ICRs, although DNMT1 likely maintains the preexisting DNA methylation at these five ICRs. Despite that DNMT1 is the major DNMT for maintaining preexisting DNA methylation at four other ICRs that includes *Gnas1A*, *Peg5*, *Mcts2*, and *Slc38a4*, DNMT3A and DNMT3B contribute to the maintenance of preexisting DNA methylation at these four ICRs in the postimplantation embryos. However, we could not rule out the possibility, with the current available data, that they might play a role, albeit unlikely, in *de novo* DNA methylation at these four ICRs in the postimplantation embryos. *B*, DNA methylation at repeats in the postimplantation embryos. DNMT1 maintains DNA methylation at the repeats including DNA repeat, LINE, SINE, and LTR. Interestingly, DNMT3A and DNMT3B function in the maintenance DNA methylation as well as *de novo* DNA methylation at these repeats after implantation. *C*, DNA methylation in the genic and intergenic regions in the postimplantation embryos. DNMT1 maintains DNA methylation in the genic (*e.g.* exon and intron), intergenic and CpG island (CGI) regions in the postimplantation embryos. Besides *de novo* DNA methylation, DNMT3A and DNMT3B are also important for the maintenance of DNA methylation in the genic and intergenic regions after implantation. DNMT, DNA methyltransferase; ICR, imprinting control region.

As is documented before, *de novo* DNA methylation occurs during implantation, which is mediated by DNMT3A and DNMT3B (3). This results in dramatic increase in DNA methylation across the genome including repeats, genic and intergenic regions, as was found in our current study (Figs. 1 and 2, 7, and S1). On the other hand, DNA methylation at the ICRs in the imprinted regions are established in the male or female germ cells, and then stably maintained in the embryos after formation of differential DNA methylation at the ICRs in the zygote after fertilization (25, 26, 30, 33). Indeed, there was no significant difference in DNA methylation at 15 ICRs in the WT postimplantation embryos compared with the ICM of WT blastocysts (Figs. 4 and 7). As expected, DNMT1 was the primary DNMT for the maintenance of preexisting DNA methylation at these ICRs after implantation (Fig. 4). This was similarly observed in the ES cell system we had used in a previous study except for the *Zrsr1* and *Peg10* ICRs that exhibited very low levels of DNA methylation in ES cells that were known to have relatively unstable DNA methylation including some imprinted regions, which had prevented us from examining maintenance DNA methylation at these two ICRs (24). Surprisingly, DNA methylation was significantly increased at the ICRs (IG-DMR, *Gpr1*, *Cdh15, H19*, and *AK008011-Ex*) of five imprinted regions after implantation (Figs. 5 and 7). This increase in DNA methylation at these ICRs resulted from *de novo* DNA methylation mediated by DNMT3A and DNMT3B as DNA methylation at these ICRs returned to the level observed in the ICM of WT blastocysts when DNMT3A and DNMT3B were absent in the *Dnmt3* DKO mutant E6.5-E7.5 epiblasts or mutant E8.5 embryos (Fig. 5). This interesting finding was not observed in the ES cell system in a previous study because *de novo* DNA methylation occurred at these ICRs after implantation, which was not replicated in the ES cells. Nevertheless, it is intriguing to observe increased DNA methylation at a subset of ICRs after implantation. It remains to be determined whether this increase in DNA methylation at these five ICRs may affect allelic expression of the corresponding imprinted genes in these imprinted regions. A possible way to look into this interesting question is to apply WGBS to the hybrid mutant embryos derived from the parents on two different genetic backgrounds so that single nucleotide polymorphisms may be used to determine the allelic expression of the imprinted genes that have been used in some previous studies (40, 41, 48, 49). Alternatively, allele-specific expression may be assessed for the imprinted genes based on expression of the reporters inserted into the loci of the target imprinted genes (50).

*De novo* DNA methylation mediated by DNMT3A and DNMT3B accounts for the increase of DNA methylation in the postimplantation embryos as global DNA methylation level in the *Dnmt3* DKO postimplantation embryos at E6.5-E8.5 was reverted back to that observed in the ICM of WT blastocysts (Fig. 1). We also found DNMT3A and DNMT3B, together with DNMT1, maintained global DNA methylation as well as newly acquired DNA methylation at the repeats, genic and intergenic regions in the postimplantation embryos (Figs. 1, 2, 7, and S1). Interestingly, there are strong evidences in this study indicating that they were involved in the maintenance of preexisting DNA methylation at the CGIs, a subset of ICRs, and certain lineage marker gene (*e.g. Th*) (Figs. 6 and 7, S6, and S17). These results at the CGIs and ICRs in this study are largely consistent with what we found in ES cells in a previous study (24). DNMT3A and DNMT3B, together with DNMT1, also contributed to the maintenance of newly acquired DNA methylation at a subset of known ICRs after implantation (Figs. 5 and 7). Despite the notion that DNMT1 maintains germline-derived DNA methylation at the ICRs in mouse embryos, these seemingly unexpected roles of two DNMT3 proteins in maintaining ICR DNA methylation in postimplantation embryos are consistent with what has been discovered in our previous study in mouse ES cells as well as in other published studies in ES cells (21, 22, 24). Unlike other established cell lines, ES cells usually are not homogenous, with both undifferentiated and differentiated cells in cell culture. Indeed, global DNA methylation levels as well as those in the genic and intergenic regions were higher in the WT ES cells compared with the ICM of WT blastocysts despite the fact that ES cells were originally derived from the ICM (Figs. 1 and 2) (24). Accordingly, there were some variations of DNA methylation levels at the ICRs comparing the embryos with the ES cells in DNA methylation analysis for the WT and mutant samples. Nevertheless, similar results were obtained at these ICRs that showed maintenance functions of DNMT3A and DNMT3B in the postimplantation embryos as well as in ES cells (Figs. 5 and 6) (24). In addition, DNA methylation was increased at five ICRs after implantation. These findings imply that DNA methylation at some ICRs may be more dynamic than what we thought before. The equilibrium in DNA methylation at these ICRs may be reached when DNMT3A and DNMT3B exert their *de novo* and maintenance DNA methylation activities, together with the maintenance DNA methylation by DNMT1, in the postimplantation embryos (Fig. 7).

DNMT3B was involved in DNA methylation in the genic regions as reported before (51). In this study, we found two DNMT3 proteins, together with DNMT1, were necessary for maintaining newly acquired DNA methylation in the genic and intergenic regions in the postimplantation embryos while *de novo* DNA methylation mediated by DNMT3A and DNMT3B resulted in dramatic increase in DNA methylation after implantation (Fig. 2). These results suggest that DNA methylation in the genic and intergenic regions is dynamic and may reach the equilibrium state when *de novo* and maintenance DNA methylation activities of DNMT3A and DNMT3B exert their functions at these regions in the postimplantation embryos, together with the maintenance DNA methylation function of DNMT1 (Fig. 7).

DNA methylation plays important roles in regulating gene expression and cell differentiation. DNA methylation was increased in two pluripotent genes, *Oct4* and *Nanog*, after implantation as their expression is silenced in the somatic cells of postimplantation embryos (Fig. S11). This increased DNA methylation resulted from *de novo* DNA methylation by DNMT3A and DNMT3B. Interestingly, they also contributed to the maintenance of newly acquired DNA methylation at *Oct4* and *Nanog* in the postimplantation embryos, together with DNMT1 (Fig. S11). Similarly, DNMT3A and DNMT3B were required for the increased DNA methylation observed at the lineage-specific genes after implantation. They maintained newly acquired DNA methylation at these examined lineage-specific genes in the postimplantation embryos, together with DNMT1 (Figs. S14, S17, and S20). Furthermore, DNMT1, DNMT3A, and DNMT3B seemed to play complementary roles in maintaining the preexisting DNA methylation at the ectodermal marker gene *Th* after implantation (Fig. S17). These results suggest that DNMT3A and DNMT3B play broad and important functions in maintaining DNA methylation and regulating gene expression in cell differentiation. It is particularly important to take this into account when DNA methyltransferases are examined for their functions in DNA methylation in development and human diseases. For example, DNMT3 proteins may exhibit essential functions in maintenance as well as *de novo* DNA methylation when their functions are examined in certain cell lineages in combination with tissue-specific *Cre* transgenes. Indeed, two DNMT3 proteins, together with DNMT1, are important for maintaining both preexisting and newly acquired DNA methylation in the postimplantation embryos. Besides their known functions in *de novo* DNA methylation, special attention may need to be paid to maintenance functions of two DNMT3 proteins in DNA methylation that may be critical for many human diseases (52, 53, 54, 55).

It is interesting that overall global DNA methylation appeared to be a bit higher in the WT E8.5 embryos compared with the epiblasts of WT E6.5-E7.5 embryos, although the WGBS data for E8.5 and the WGBS data for E6.5-E7.5 embryos were derived from two different studies (Fig. 1) (42, 43). However, the remaining DNA methylation levels were similar in the *Dnmt3* DKO mutant E6.5-E7.5 epiblasts or mutant E8.5 embryos because there was no significant difference in DNA methylation levels comparing *Dnmt3* DKO mutant E6.5-E7.5 epiblasts or mutant E8.5 embryos with the ICM of the WT blastocysts that was the same in all comparisons (Fig. 1). This was the same for the genic and intergenic regions as well (Fig. 2). Based on these results, we hypothesize that global DNA methylation including the genic and intergenic regions increases as the embryos develop into more advanced stages from E6.5-E7.5 to E8.5. Indeed, organogenesis such as the heart formation occurs during this transition, which may require DNA methylation to regulate expression of many genes essential for organogenesis. Nevertheless, DNA methylation levels at most repeats were similarly high (around 80–85%) in the WT E6.5-E7.5 epiblasts or E8.5 embryos (Fig. S1). This suggests that DNA methylation may have reached plateau at 80 to 85% at these repeat regions in the postimplantation embryos, which may be sufficient to keep the repeats in the transcriptionally silenced state.

Another interesting observation in this study is regarding *de novo* and maintenance DNA methylation at the CGIs in the postimplantation embryos. It appeared to be increased gradually from the epiblasts of WT E6.5 embryos to the epiblasts of WT E7.5 embryos, then continued to go up a lot more in the WT E8.5 embryos (Fig. S6). These results suggest that *de novo* DNA methylation occurs at the CGIs after implantation, which appears to continue in the postimplantation embryos as the embryos grow. Intriguingly, there is strong evidence that two DNMT3 proteins, together with DNMT1, are required for maintaining the preexisting DNA methylation at the CGIs in the epiblasts of E6.5-E7.5 embryos, while they maintain the newly acquired DNA methylation at the CGIs in the postimplantation embryos at E8.5 (Fig. S6). Therefore, we hypothesize that neither DNMT1 nor two DNMT3 proteins are sufficient to maintain the preexisting or newly acquired DNA methylation at the CGIs by themselves. Two DNMT3 proteins, together with DNMT1, are required for the maintenance of DNA methylation at the CGIs after implantation.

## Experimental procedures

### Methods

#### Whole-genome bisulfite sequencing analysis data

For the data used in the WGBS analyses of this study, they were deposited in the Gene Expression Omnibus (GEO), as were reported in the previously published studies (42, 43, 44, 45) (Table 1). Specifically, the WGBS data for the gametes and the ICM of the WT blastocysts were from the GEO accession number GSE56697 in the first published study (44). The second set of the WGBS data for the ICM of the WT blastocysts were derived from the GEO accession number GSE84236 in the second published study (45). These two sets of WGBS data for the ICM of WT blastocysts from two different studies were combined in our DNA methylation analysis as is indicated in the Table S3. The WGBS data for the epiblast of E6.5-E7.5 embryos were taken from the GEO accession number GSE162903 for the epiblast samples (n = 3) of the WT, *Dnmt1* mutant (*Dnmt1* KO), *Dnmt3* double mutant (*Dnmt3* DKO), and *Dnmt* triple mutant (*Dnmt* TKO) mouse embryos at E6.5 or E7.5 in this published study (43). And the WGBS data for E8.5 embryos were from the GEO accession number GSE130735 for the embryo samples (n = 2) of the WT, *Dnmt1* KO and *Dnmt3* DKO mutant mouse embryos at E8.5 in another published study (42).

**Table 1:** Key resource table

Deposited data		
WGBS data for gametes and the ICM of E3.5 WT mouse embryos	Wang, L., 2014 (44)	GEO: GSE56697
WGBS data for the ICM of E3.5 WT mouse embryos	Smith, Z.D., 2017 (45)	GEO: GSE84236
WGBS data for the epiblasts of E6.5 and E7.5 mouse embryos	Li, Q., 2023 (43)	GEO: GSE162903
WGBS data for E8.5 mouse embryo	Dahlet, T., 2020 (42)	GEO: GSE130735
Software and algorithms		
BitMapperBS (v1.0.2.1)	Cheng, H., 2018 (13)	https://github.com/chhylp123/BitMapperBS
BedTools (v2.31.0)	Quinlan and Hall, 2010 (56)	https://bedtools.readthedocs.io/en/latest/
Trim Galore (v0.6.10)	Babraham Institute	https://www.bioinformatics.babraham.ac.uk/projects/trim_galore/
Picard MarkDuplicates (v2.18.29)	Broad Institute	https://broadinstitute.github.io/picard/
MethylDackel (v0.3.0)		https://github.com/dpryan79/MethylDackel

#### Analyses of the published WGBS data in this study

The WGBS data for DNA methylation analysis in this study were from the previously published articles (42, 43, 44, 45). Two sets of WGBS data from two different studies were combined in our DNA methylation analysis for the ICM of WT blastocysts (Table S3) (44, 45). The sequence reads from the deposited original data were trimmed with the software Trim Galore v0.6.10 to remove the bases of low-quality sequencing (Table 1) (https://github.com/FelixKrueger/TrimGalore). The trimmed reads were aligned to the mouse genome (mm10) using the software named BitMapperBS (v1.0.2.1) with default parameters. Any duplicate sequences were cleaned with the software tool called Picard MarkDuplicates (v2.18.29). The methylation calls for the CpG sites were extracted by using the software MethylDackel (v0.3.0). Only those CpGs that were covered by a minimum of three sequence reads were retained for further analyses in each WGBS sample.

#### Genomic features

The annotations for LINE, LTR, and SINE were downloaded from the RepeatMasker tracks on the Browser (mm10) run by University of California, Santa Cruz browser (UCSC; https://genome.ucsc.edu/cgi-bin/hgTables), whereas the annotations for CGIs were downloaded from the CpG Islands track of the same browser. The gene annotations were from the RefSeq track and the Promoters were defined as the region within ± 2 kb of the transcription start site according to the RefSeq annotation on the same UCSC Browser. The sequences of the ICRs of 24 imprinted regions shown in Table S2 were taken from the previous studies (40, 47). In each case, the methylation level of an individual feature was calculated by average methylation of all CpGs within the feature that were covered more than three times of the unique reads by BedTools (v2.31.0).

#### DNA methylation levels

DNA methylation was quantified for each CpG site of the genomic regions such as repeats or ICRs by using the number of sequence reads with a methylated CpG site divided by the total number of good-quality sequence reads for this CpG site. Average DNA methylation levels were calculated based on all CpG sites in the genomic regions. Similarly, DNA methylation was measured for 24 known ICRs in the ICM of the WT blastocysts from two different studies, in the epiblasts of WT or *Dnmt* mutant embryos lacking one or two or three DNMT proteins and in the WT or *Dnmt1* mutant or *Dnmt3* DKO E8.5 embryos (Tables S2–S6). In Figure 3, the values indicating the methylation levels of all ICRs were quantified with the Z scores and their methylation levels were shown on the figures according to their mean values plus standard deviation. DNA methylation was also analyzed for three intergenic regions and some genes involved in pluripotency or differentiation of three germ layers (Tables S1 and S7).

### Statistical analyses

Statistical analysis was carried out by using one-way ANOVA with Dunnett multiple comparison test. The statistical analysis method for each figure is also provided in the figure legend of each figure. The numbers in the bar graphs for these figures are mean ± SEM. Statistical significance values are as follows: ∗*p* < 0.05; ∗∗*p* < 0.01; ∗∗∗*p* < 0.001. ns, not statistically significant with *p*-value more than 0.1 for this study as indicated on the figure legends.

## Data availability

The WGBS data analyzed in this study are from the Gene Expression Omnibus (GEO) with the accession number GSE56697, GSE84236, GSE162903, and GSE130735. There are no original WGBS data generated in this study that may need to be deposited in the public databases. Any information related to the analyses carried out in this study is available from the lead contact upon request, Dr Xiajun Li (lixj1@shanghaitech.edu.cn).

## Supporting information

This article contains supporting information that includes the Supplemental Figures and their Legends, Supplemental Tables.

## Conflict of interest

The authors declare that they have no conflicts of interest with the contents of this article.

## Supporting information

Dnmt SI figure-JBC-r1

Manu-jbc-sup-24-rev1
