# Supplementary material for: Regulation of de novo and maintenance DNA methylation by DNA methyltransferases in postimplantation embryos
Source: J Biol Chem. 2024 Nov 13;301(1):107990. doi: 10.1016/j.jbc.2024.107990 (PMC11742614; doi:10.1016/j.jbc.2024.107990)
Supplement: Dnmt SI figure-JBC-r1 [file mmc1.pdf]

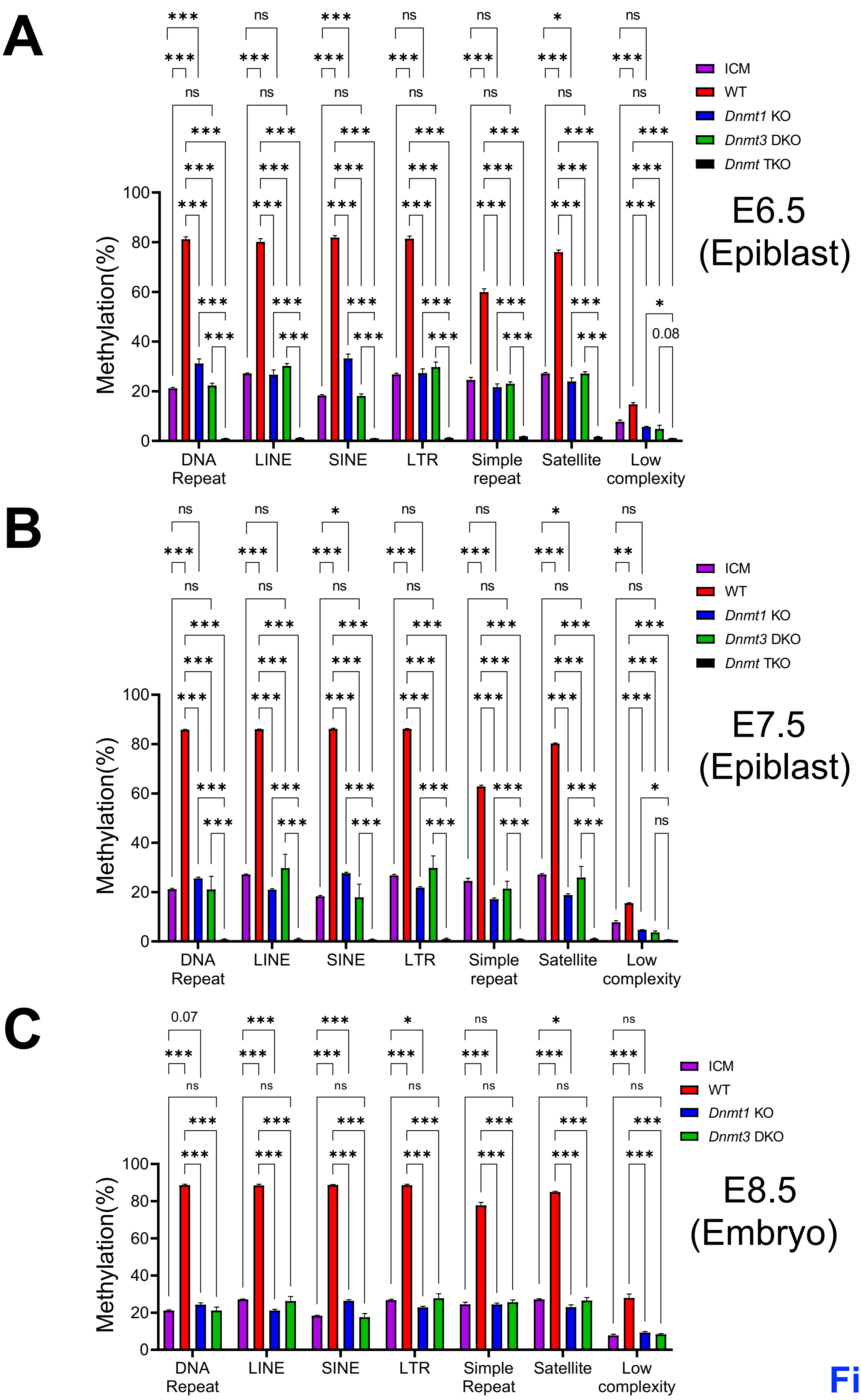
A
E6.5
(Epiblast)
B
E7.5
(Epiblast)
C
E8.5
(Embryo)
ICM
WT
Dnmt1 KO
Dnmt3 DKO
Dnmt TKO
Methylation(%)
DNA Repeat
LINE
SINE
LTR
Simple repeat
Satellite
Low complexity
0.08
0.07

Fig. S1

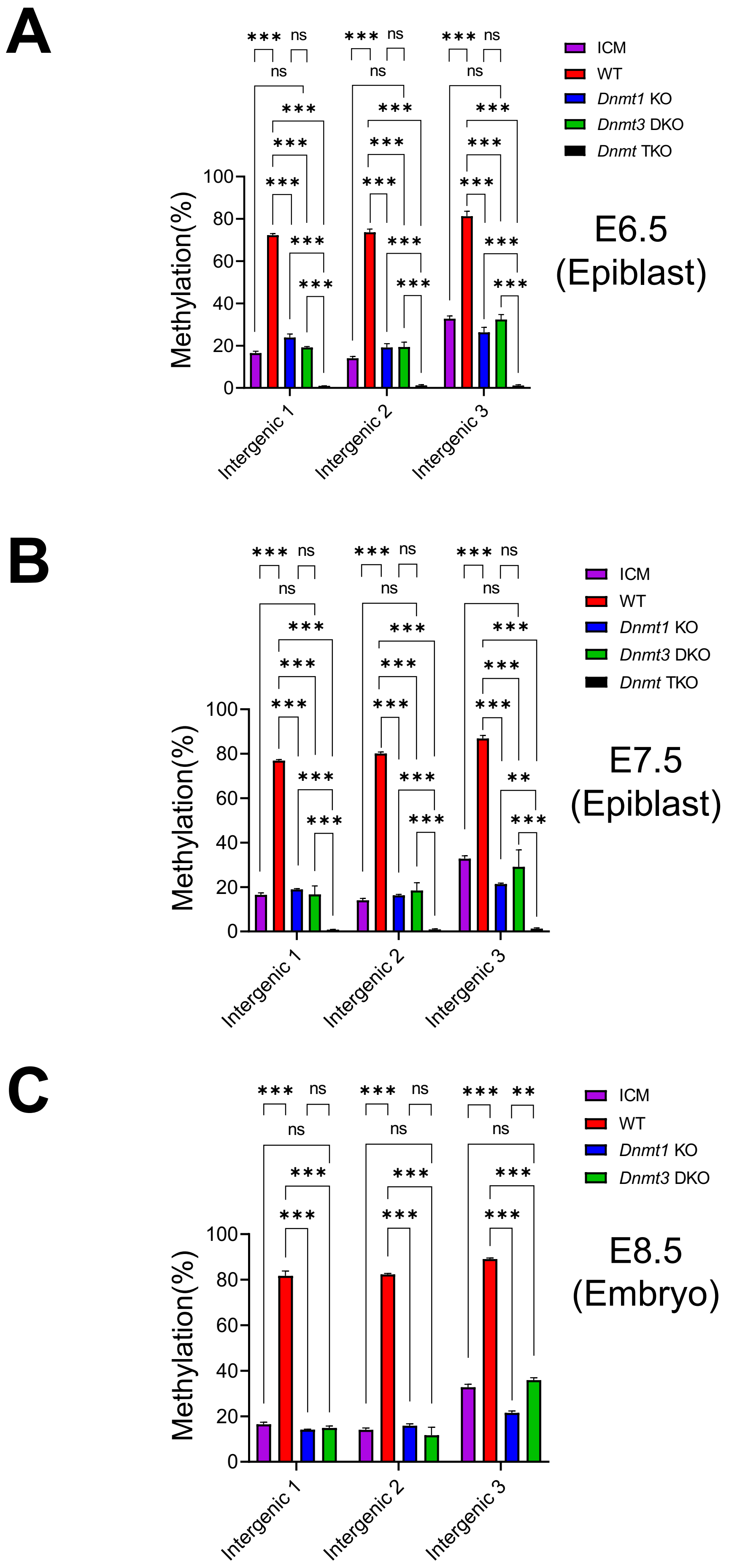
A
B
C
ICM
WT
Dnmt1 KO
Dnmt3 DKO
Dnmt TKO
Methylation(%)
Intergenic 1
Intergenic 2
Intergenic 3
E6.5
(Epiblast)
E7.5
(Epiblast)
E8.5
(Embryo)
***
**
ns

Fig. S2

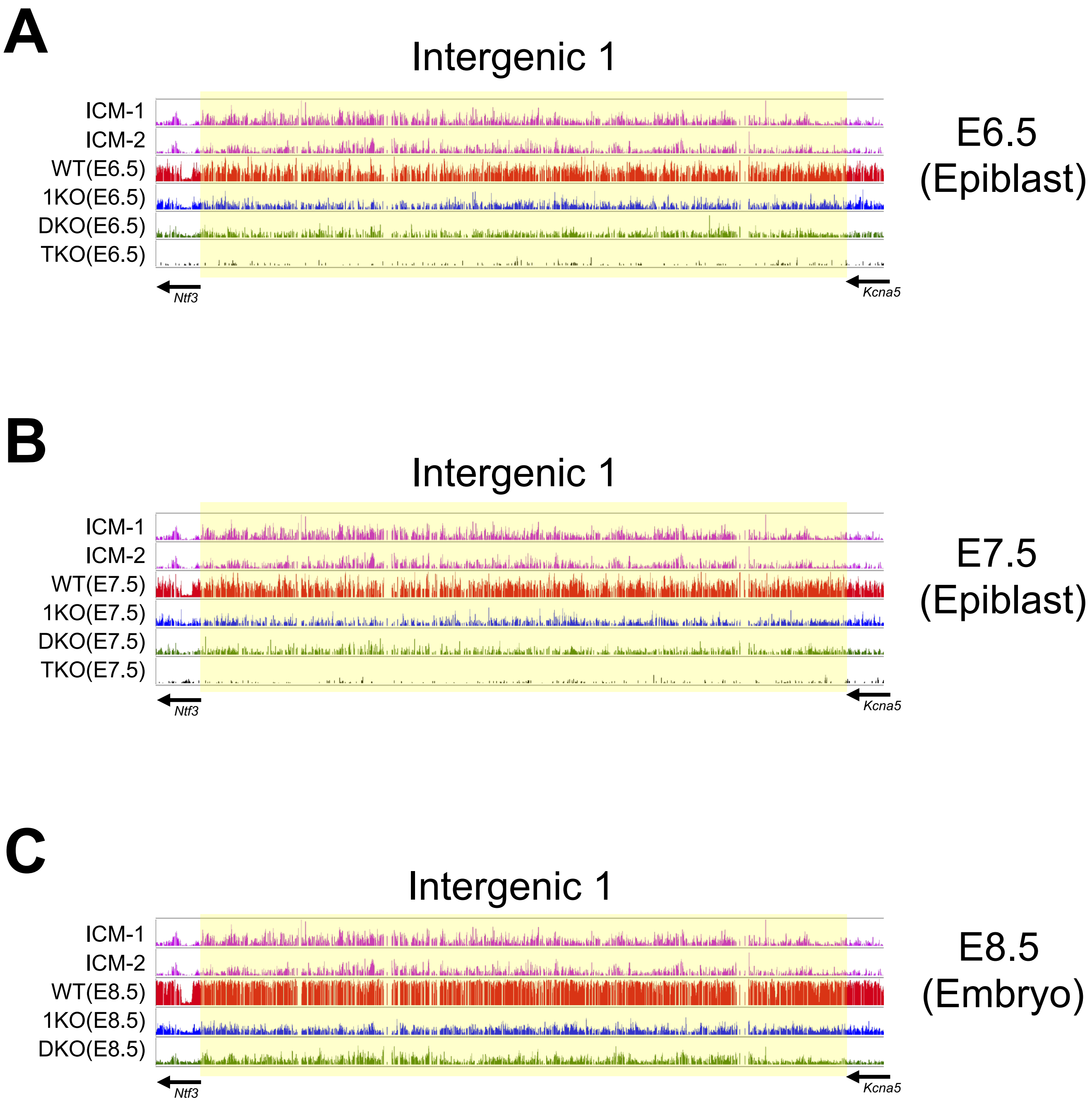
A
Intergenic 1
ICM-1
ICM-2
WT(E6.5)
1KO(E6.5)
DKO(E6.5)
TKO(E6.5)
Ntf3
Kcna5
E6.5
(Epiblast)
B
Intergenic 1
ICM-1
ICM-2
WT(E7.5)
1KO(E7.5)
DKO(E7.5)
TKO(E7.5)
Ntf3
Kcna5
E7.5
(Epiblast)
C
Intergenic 1
ICM-1
ICM-2
WT(E8.5)
1KO(E8.5)
DKO(E8.5)
Ntf3
Kcna5
E8.5
(Embryo)

Fig. S3

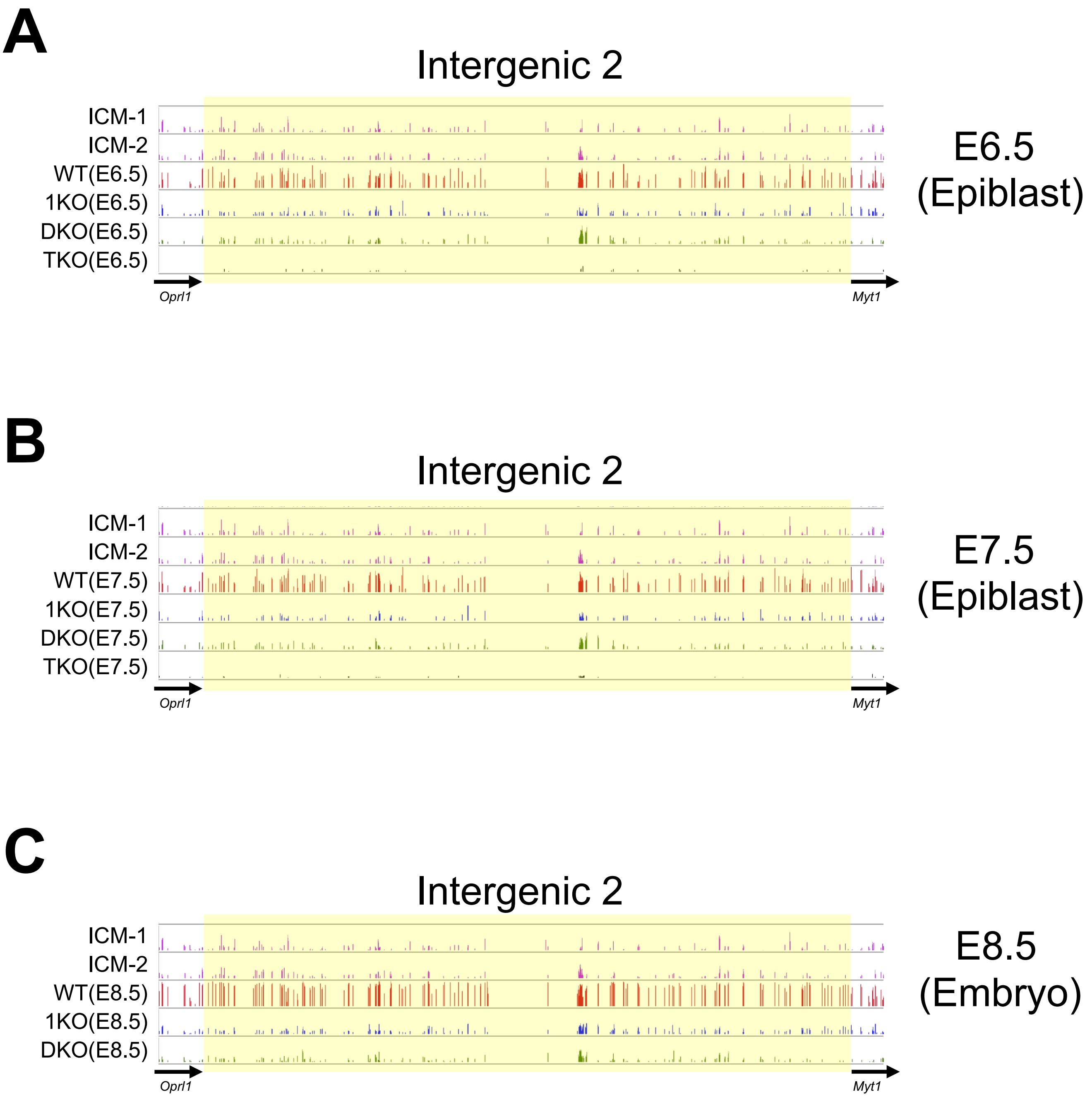
A
Intergenic 2
ICM-1
ICM-2
WT(E6.5)
1KO(E6.5)
DKO(E6.5)
TKO(E6.5)
Oprl1
Myt1
E6.5
(Epiblast)
B
Intergenic 2
ICM-1
ICM-2
WT(E7.5)
1KO(E7.5)
DKO(E7.5)
TKO(E7.5)
Oprl1
Myt1
E7.5
(Epiblast)
C
Intergenic 2
ICM-1
ICM-2
WT(E8.5)
1KO(E8.5)
DKO(E8.5)
Oprl1
Myt1
E8.5
(Embryo)

Fig. S4

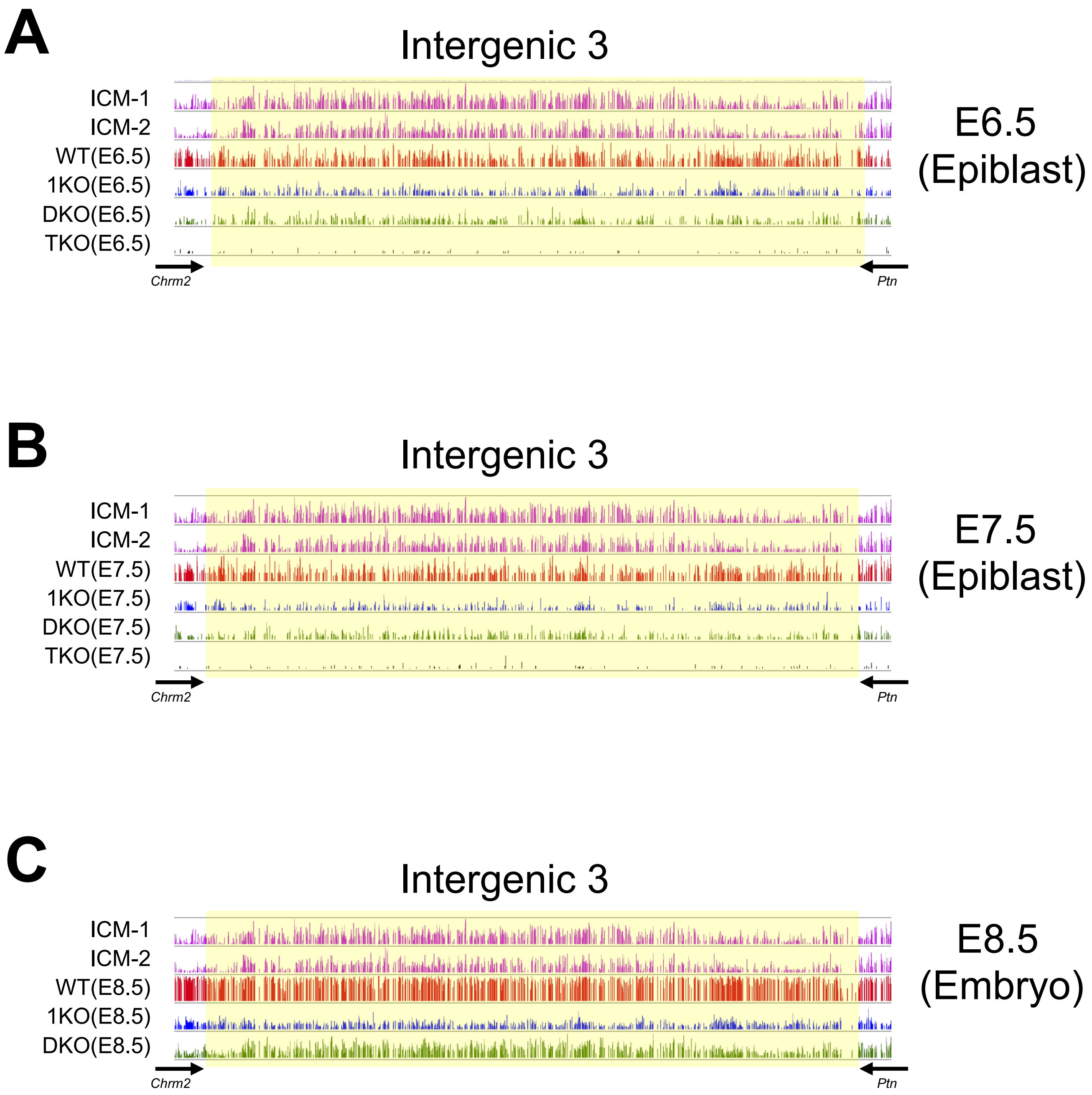
A
Intergenic 3
ICM-1
ICM-2
WT(E6.5)
1KO(E6.5)
DKO(E6.5)
TKO(E6.5)
Chrm2
Ptn
E6.5
(Epiblast)
B
Intergenic 3
ICM-1
ICM-2
WT(E7.5)
1KO(E7.5)
DKO(E7.5)
TKO(E7.5)
Chrm2
Ptn
E7.5
(Epiblast)
C
Intergenic 3
ICM-1
ICM-2
WT(E8.5)
1KO(E8.5)
DKO(E8.5)
Chrm2
Ptn
E8.5
(Embryo)

Fig. S5

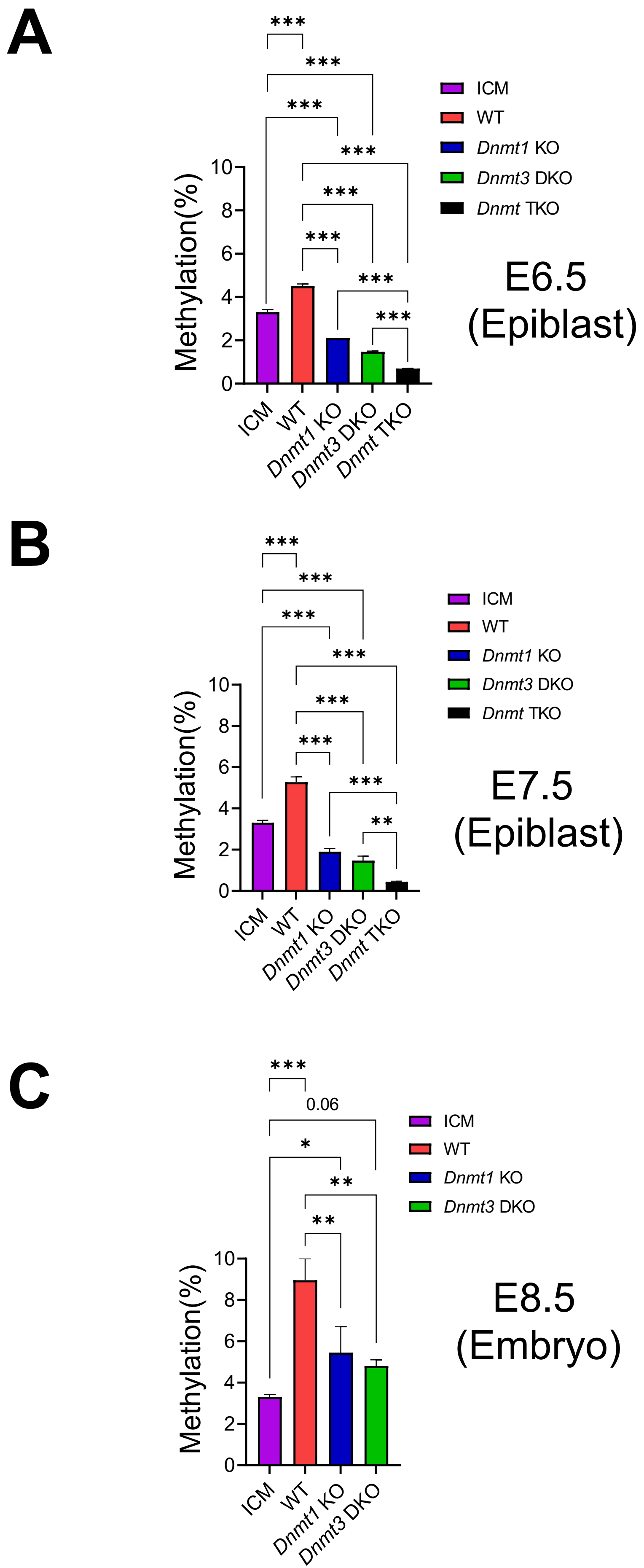
A
B
C
Methylation(%)
ICM
WT
Dnmt1 KO
Dnmt3 DKO
Dnmt TKO
E6.5
(Epiblast)
E7.5
(Epiblast)
E8.5
(Embryo)
***
**
*
0.06
0
2
4
6
8
10

Fig. S6

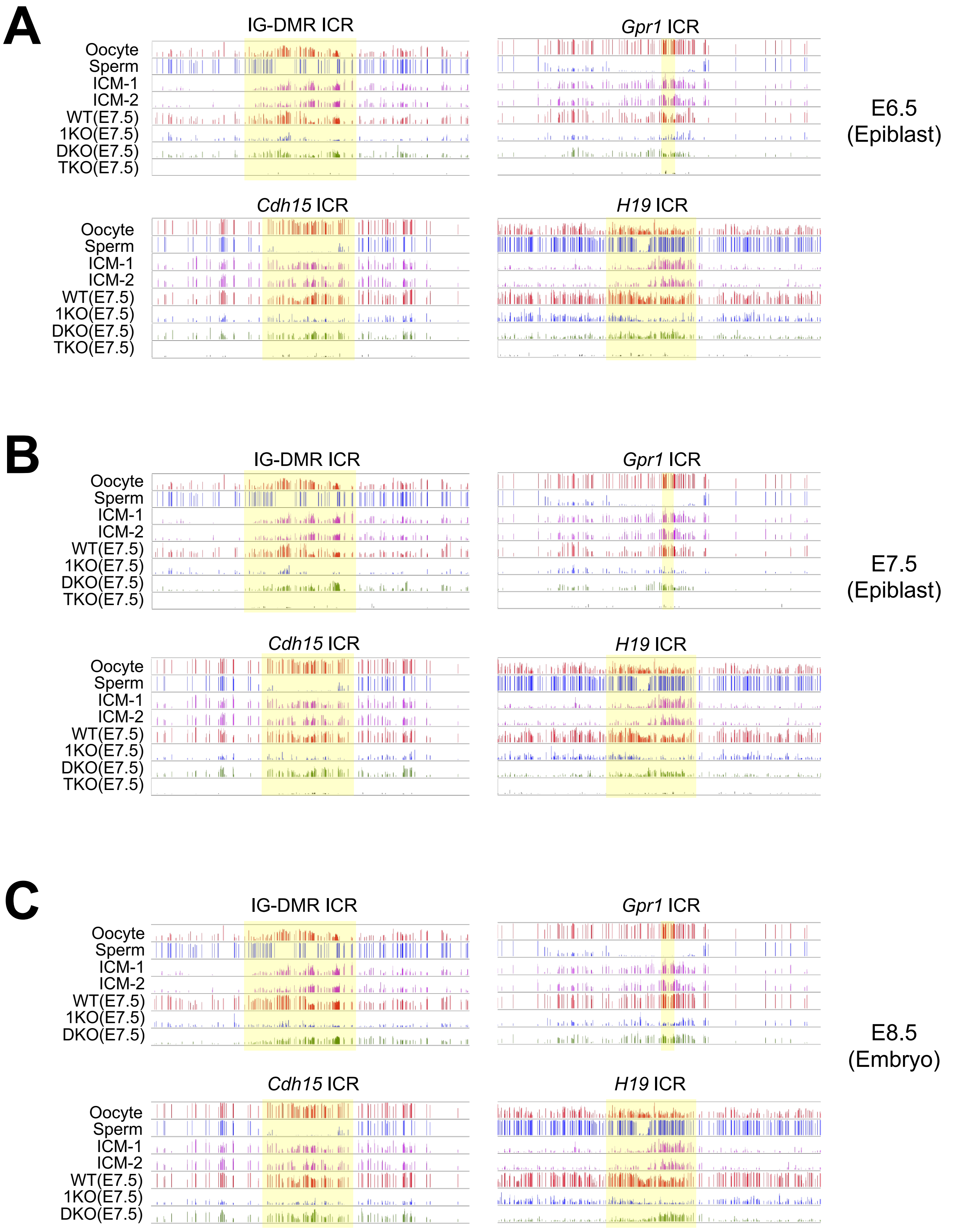
A
IG-DMR ICR
Gpr1 ICR
Cdh15 ICR
H19 ICR
Oocyte
Sperm
ICM-1
ICM-2
WT(E7.5)
1KO(E7.5)
DKO(E7.5)
TKO(E7.5)
E6.5
(Epiblast)
B
E7.5
(Epiblast)
C
E8.5
(Embryo)

Fig. S7

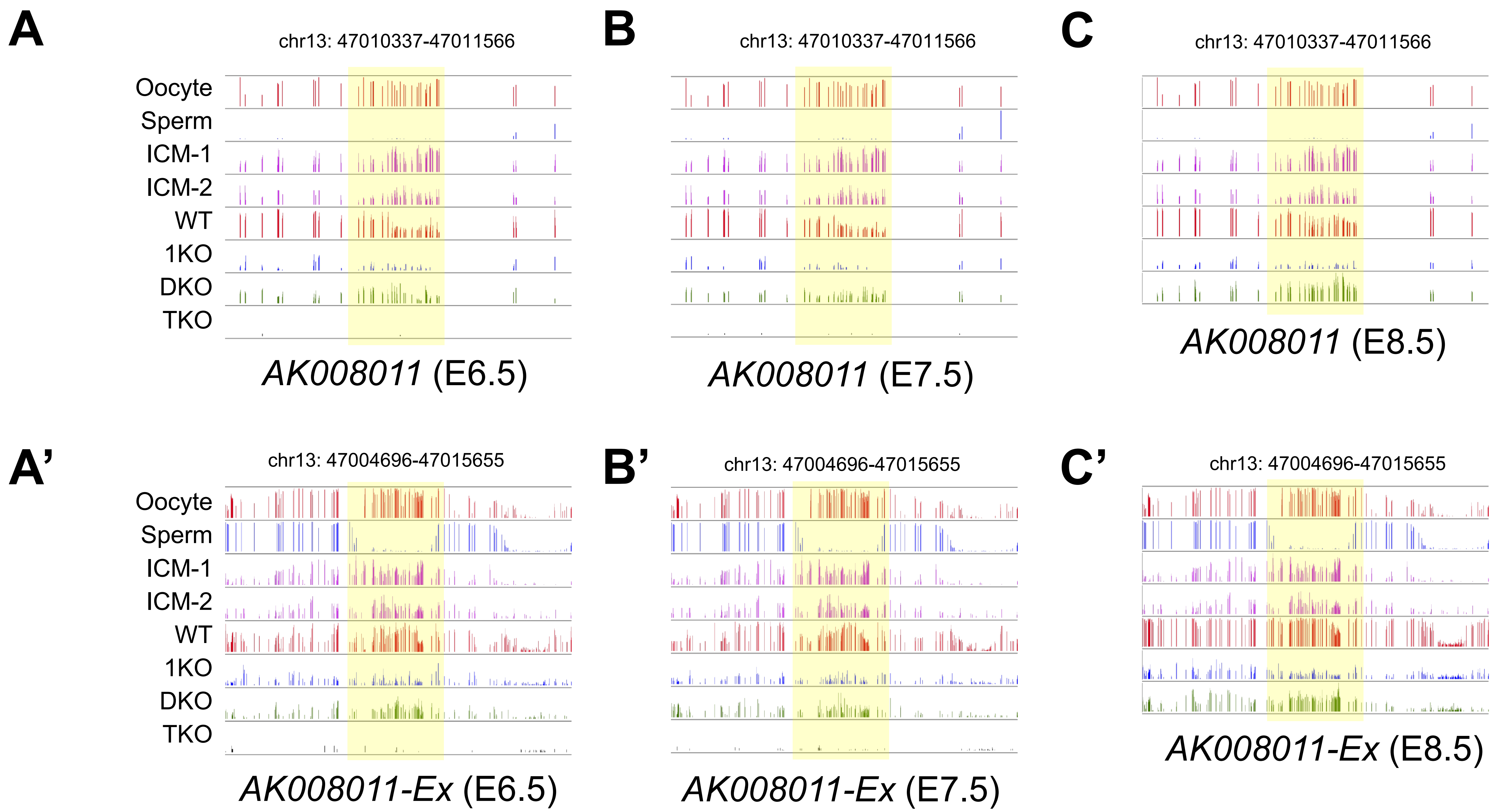
A
chr13: 47010337-47011566
Oocyte
Sperm
ICM-1
ICM-2
WT
1KO
DKO
TKO
AK008011 (E6.5)
B
chr13: 47010337-47011566
AK008011 (E7.5)
C
chr13: 47010337-47011566
AK008011 (E8.5)
A’
chr13: 47004696-47015655
Oocyte
Sperm
ICM-1
ICM-2
WT
1KO
DKO
TKO
AK008011-Ex (E6.5)
B’
chr13: 47004696-47015655
AK008011-Ex (E7.5)
C’
chr13: 47004696-47015655
AK008011-Ex (E8.5)

Fig. S8

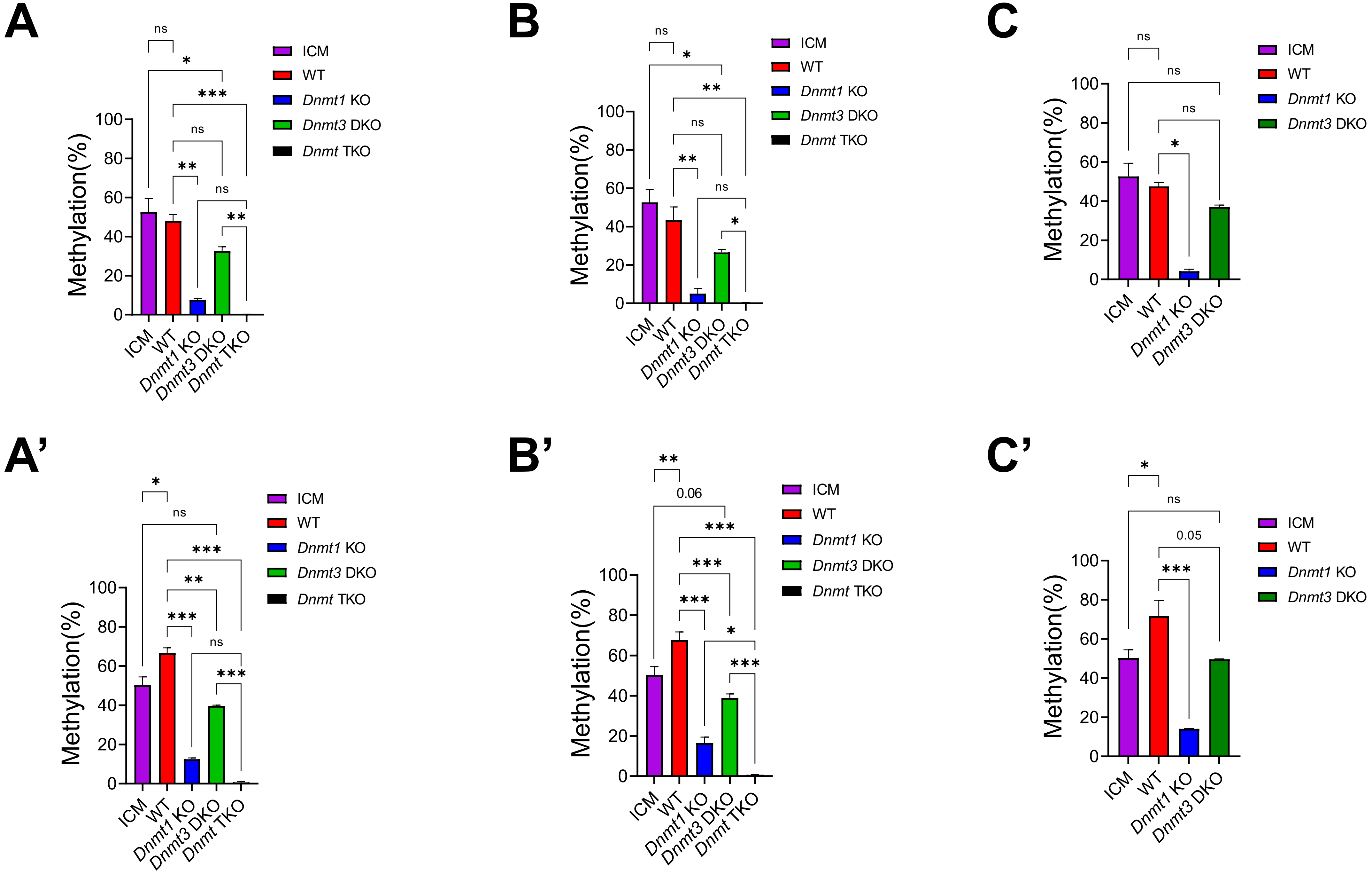
A
B
C
A'
B'
C'
Methylation(%)
ICM
WT
Dnmt1 KO
Dnmt3 DKO
Dnmt TKO
ns
*
**
***
0.06
0.05
0
20
40
60
80
100

Fig. S9

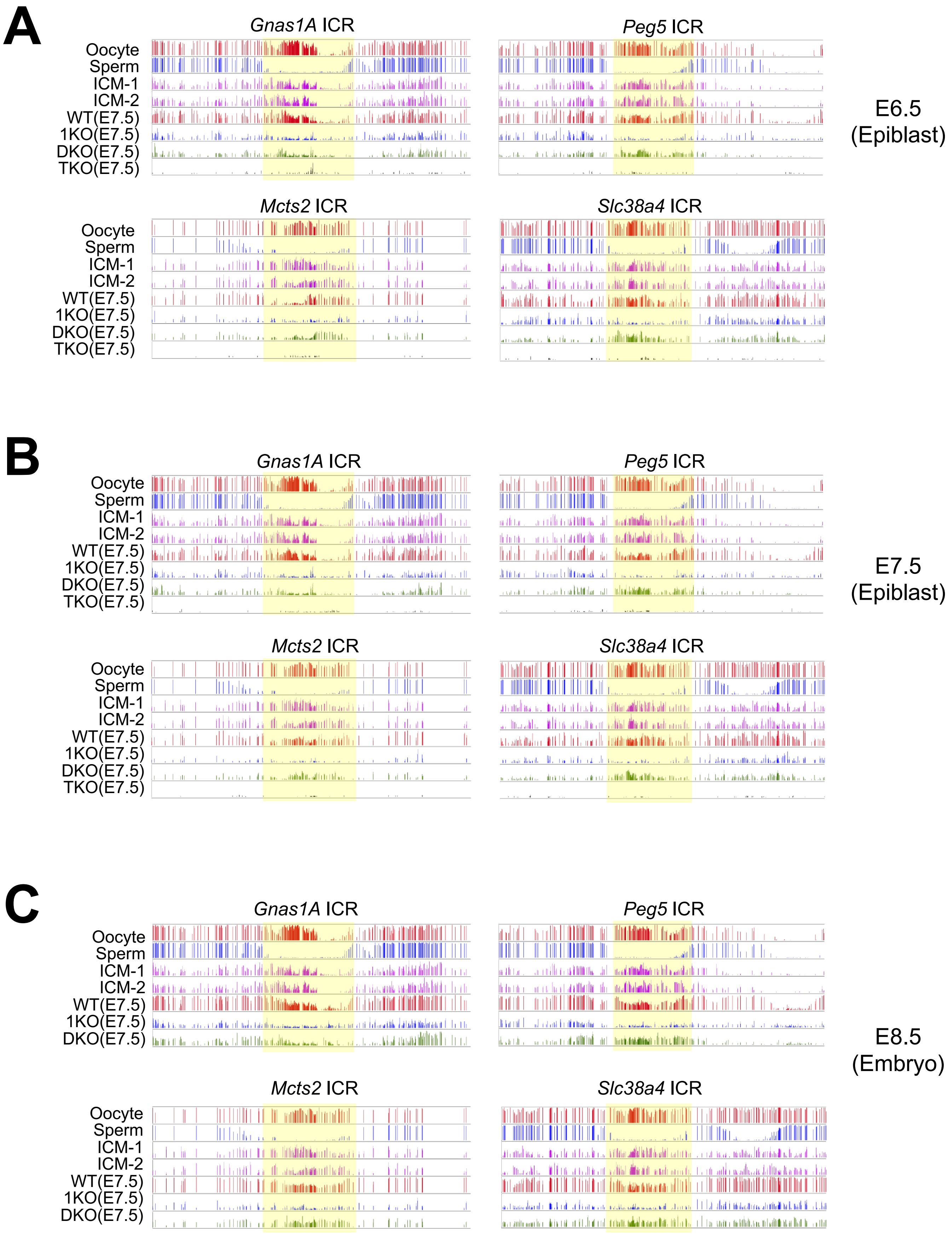
A
Gnas1A ICR
Peg5 ICR
Oocyte
Sperm
ICM-1
ICM-2
WT(E7.5)
1KO(E7.5)
DKO(E7.5)
TKO(E7.5)
E6.5
(Epiblast)
Mcts2 ICR
Slc38a4 ICR
B
Gnas1A ICR
Peg5 ICR
E7.5
(Epiblast)
Mcts2 ICR
Slc38a4 ICR
C
Gnas1A ICR
Peg5 ICR
E8.5
(Embryo)
Mcts2 ICR
Slc38a4 ICR

Fig. S10

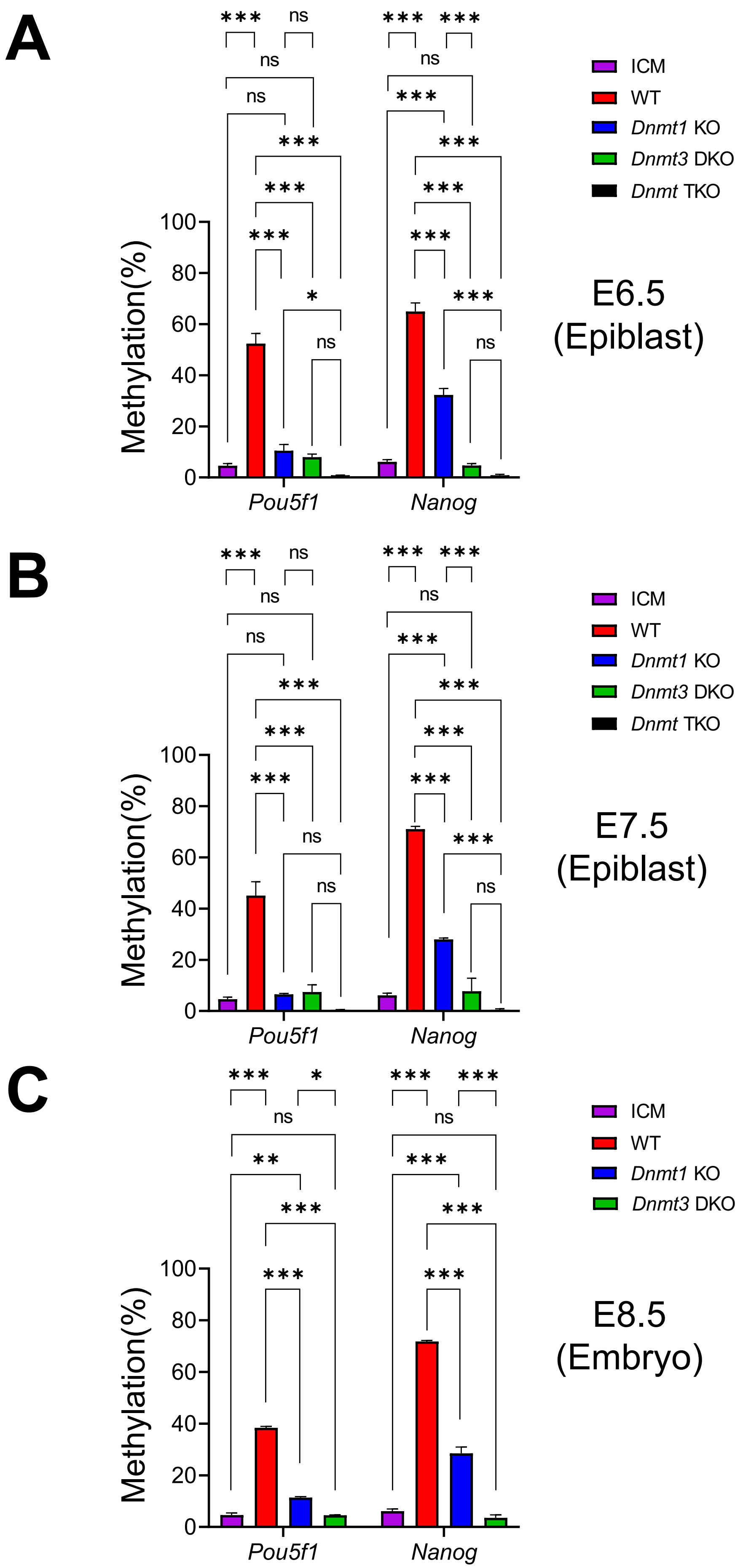
A
B
C
Methylation(%)
Pou5f1
Nanog
ICM
WT
Dnmt1 KO
Dnmt3 DKO
Dnmt TKO
E6.5
(Epiblast)
E7.5
(Epiblast)
E8.5
(Embryo)
ns
***
**
*

Fig. S11

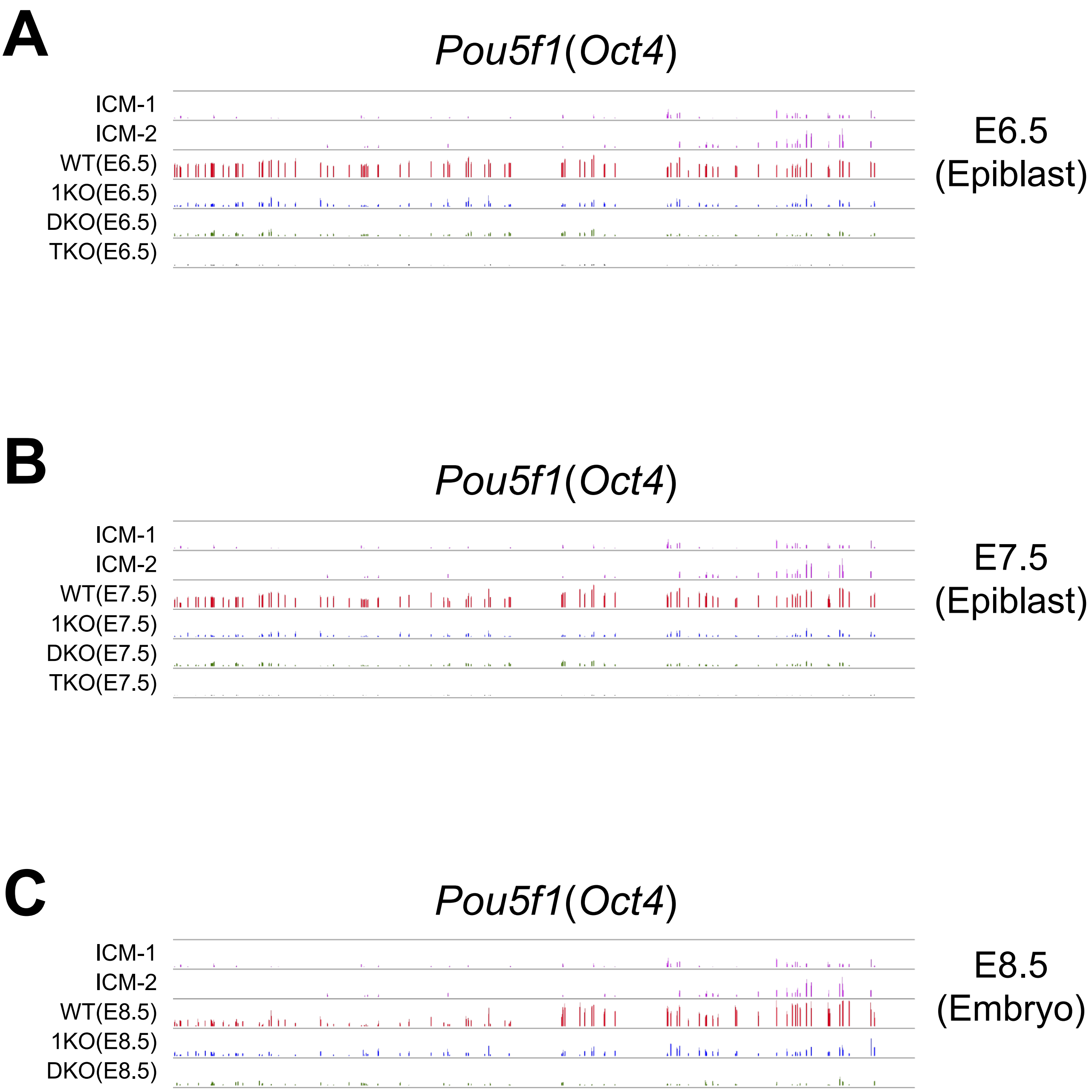
A
Pou5f1(*Oct4*)
ICM-1
ICM-2
WT(E6.5)
1KO(E6.5)
DKO(E6.5)
TKO(E6.5)
E6.5
(Epiblast)
B
Pou5f1(*Oct4*)
ICM-1
ICM-2
WT(E7.5)
1KO(E7.5)
DKO(E7.5)
TKO(E7.5)
E7.5
(Epiblast)
C
Pou5f1(*Oct4*)
ICM-1
ICM-2
WT(E8.5)
1KO(E8.5)
DKO(E8.5)
E8.5
(Embryo)

Fig. S12

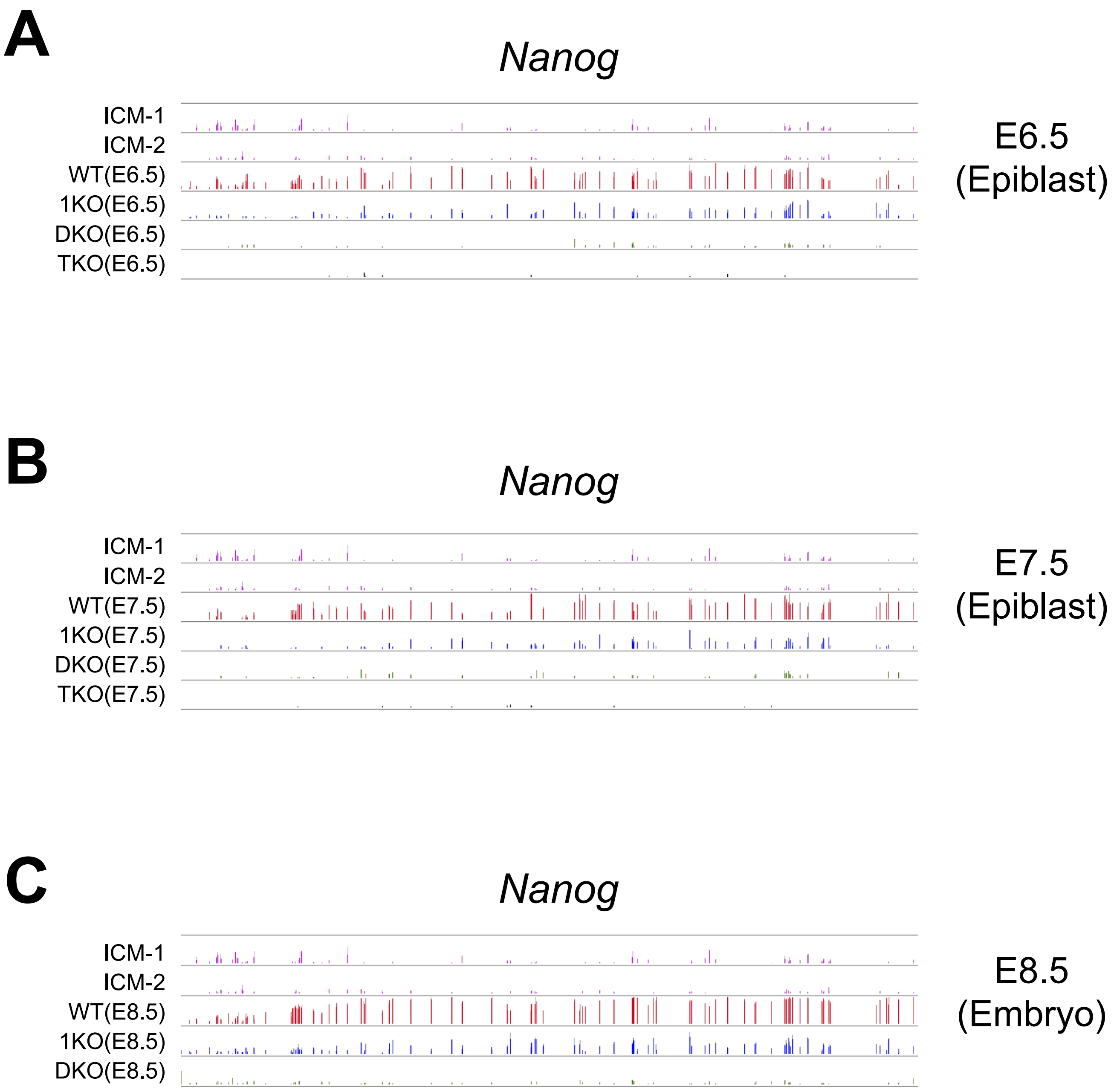
A
Nanog
ICM-1
ICM-2
WT(E6.5)
1KO(E6.5)
DKO(E6.5)
TKO(E6.5)
E6.5
(Epiblast)
B
Nanog
ICM-1
ICM-2
WT(E7.5)
1KO(E7.5)
DKO(E7.5)
TKO(E7.5)
E7.5
(Epiblast)
C
Nanog
ICM-1
ICM-2
WT(E8.5)
1KO(E8.5)
DKO(E8.5)
E8.5
(Embryo)

Fig. S13

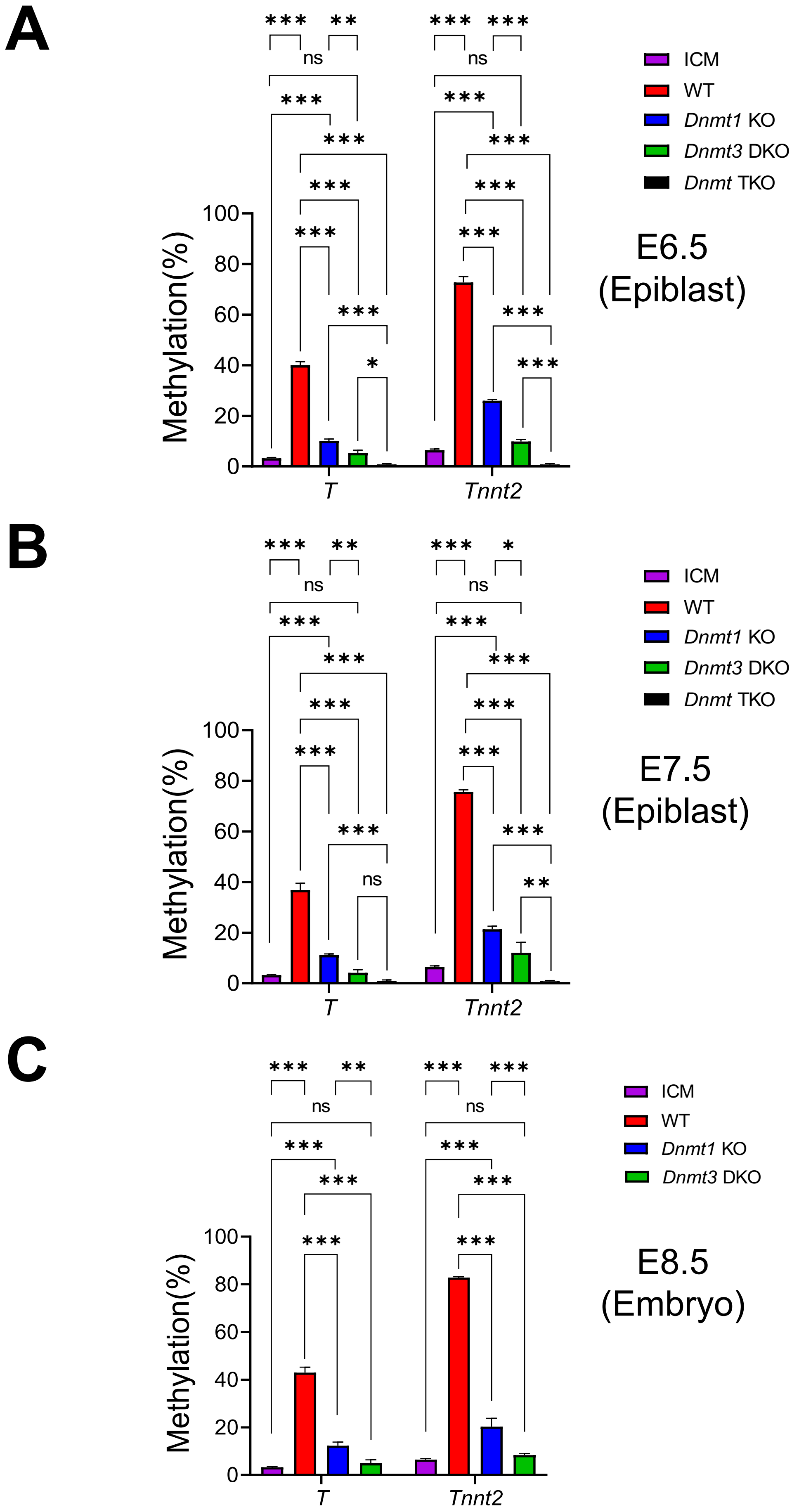
A
B
C
ICM
WT
Dnmt1 KO
Dnmt3 DKO
Dnmt TKO
E6.5
(Epiblast)
E7.5
(Epiblast)
E8.5
(Embryo)
Methylation(%)
T
Tnnt2
***
**
*
ns
0
20
40
60
80
100

Fig. S14

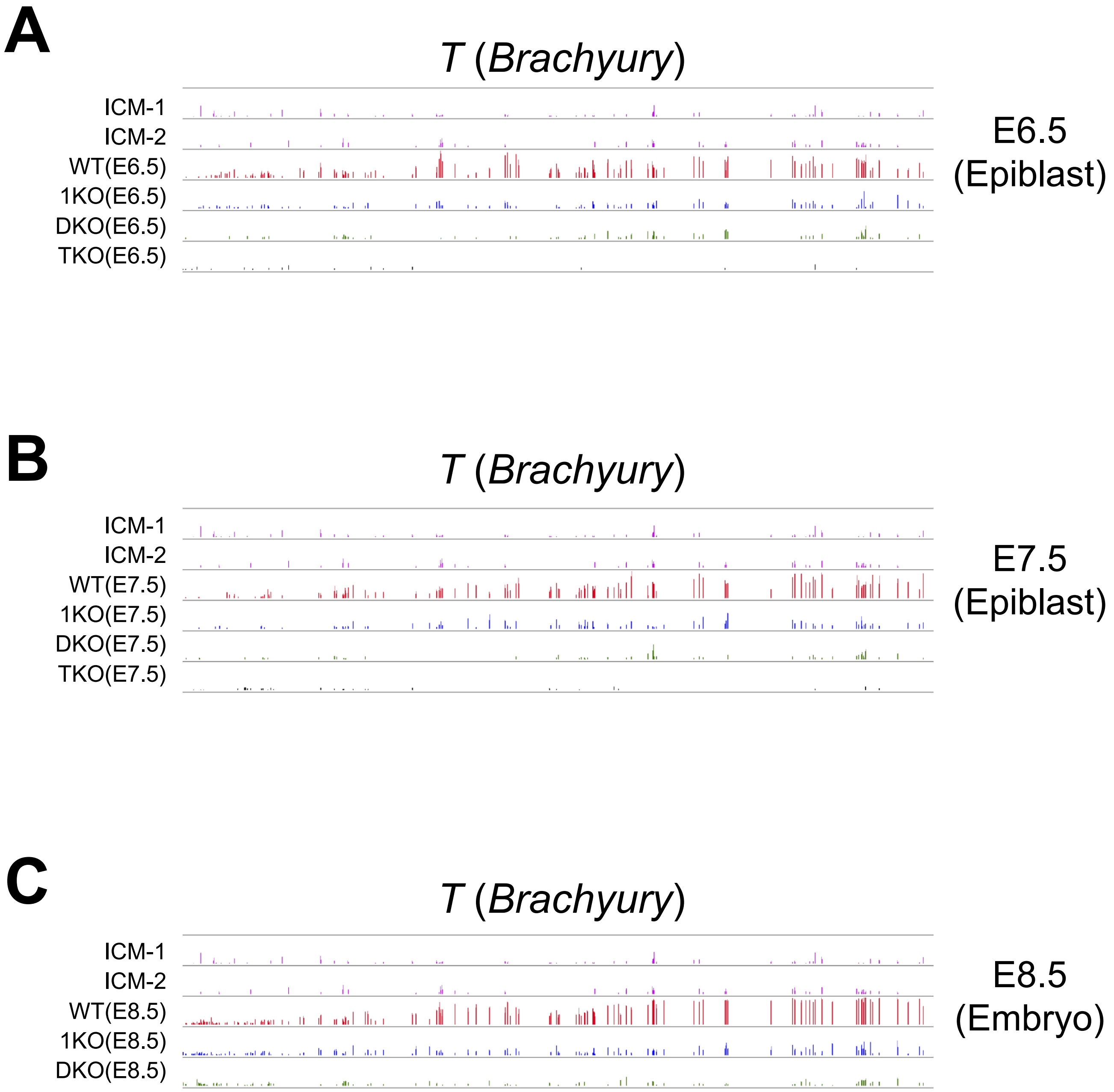
A
T (Brachyury)
ICM-1
ICM-2
WT(E6.5)
1KO(E6.5)
DKO(E6.5)
TKO(E6.5)
E6.5
(Epiblast)
B
T (Brachyury)
ICM-1
ICM-2
WT(E7.5)
1KO(E7.5)
DKO(E7.5)
TKO(E7.5)
E7.5
(Epiblast)
C
T (Brachyury)
ICM-1
ICM-2
WT(E8.5)
1KO(E8.5)
DKO(E8.5)
E8.5
(Embryo)

Fig. S15

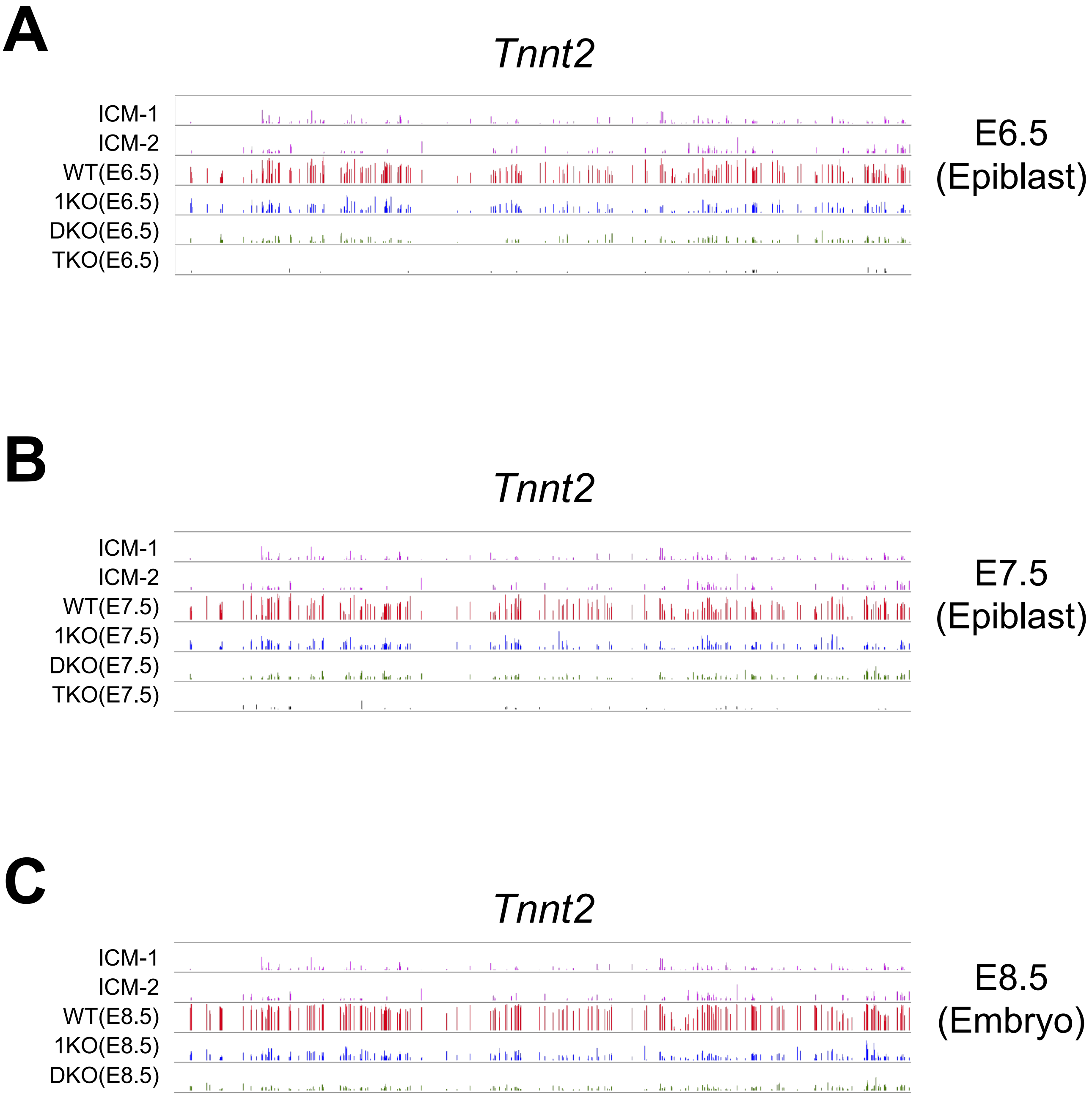
A
Tnnt2
ICM-1
ICM-2
WT(E6.5)
1KO(E6.5)
DKO(E6.5)
TKO(E6.5)
E6.5
(Epiblast)
B
Tnnt2
ICM-1
ICM-2
WT(E7.5)
1KO(E7.5)
DKO(E7.5)
TKO(E7.5)
E7.5
(Epiblast)
C
Tnnt2
ICM-1
ICM-2
WT(E8.5)
1KO(E8.5)
DKO(E8.5)
E8.5
(Embryo)

Fig. S16

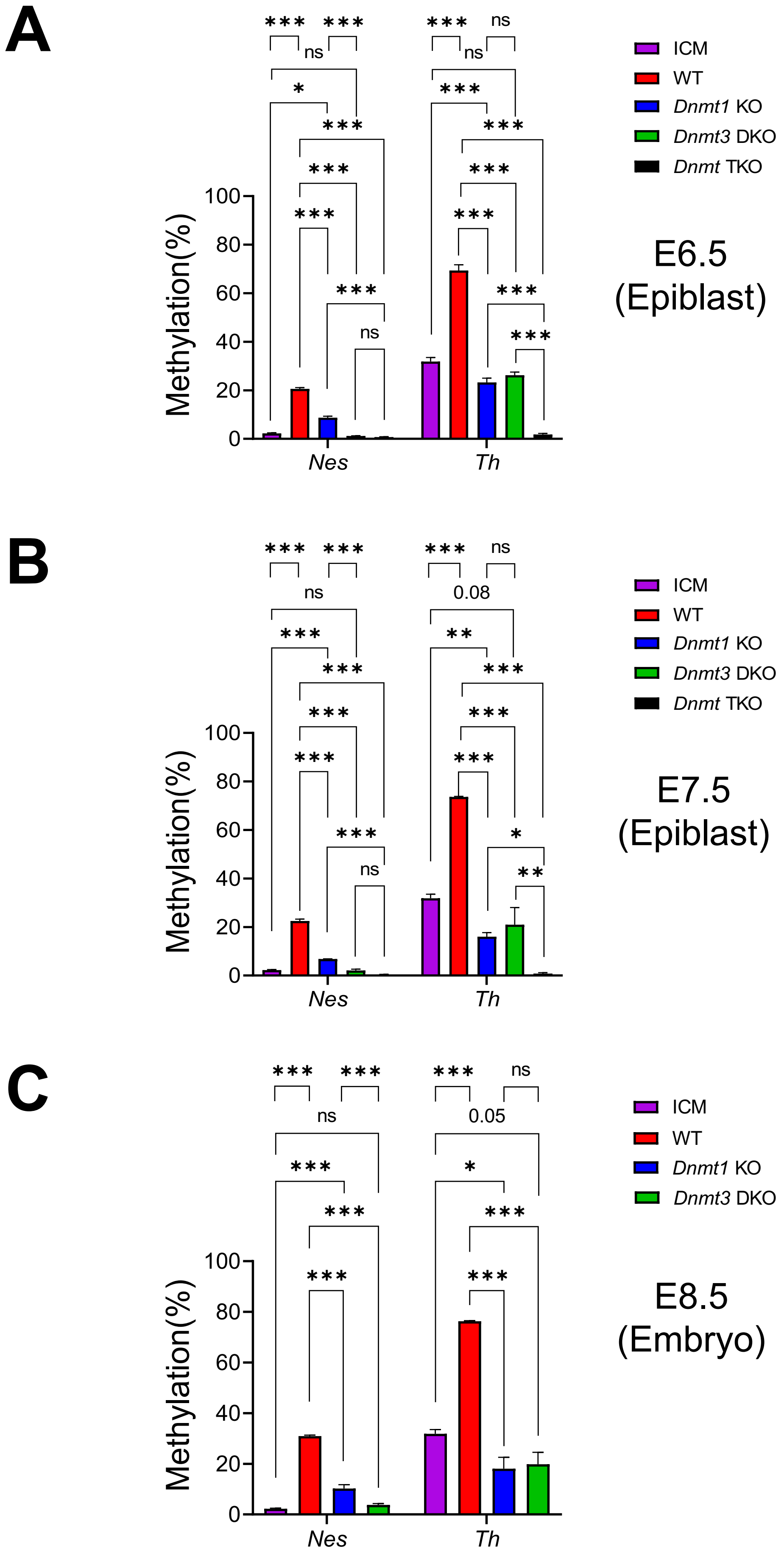
A
B
C
ICM
WT
Dnmt1 KO
Dnmt3 DKO
Dnmt TKO
E6.5
(Epiblast)
E7.5
(Epiblast)
E8.5
(Embryo)
Methylation(%)
Nes
Th
ns
0.08
0.05

Fig. S17

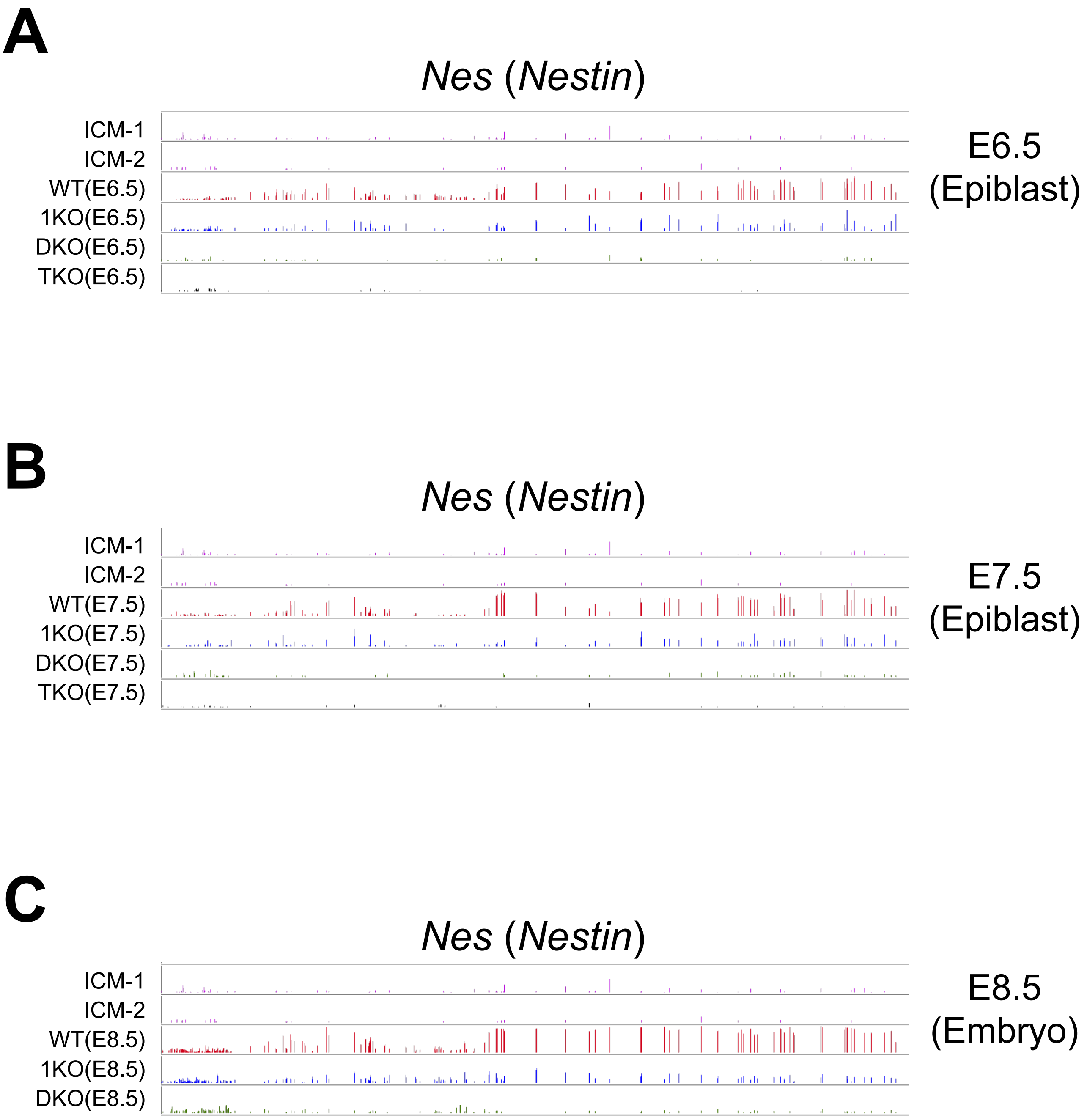
A
Nes (Nestin)
ICM-1
ICM-2
WT(E6.5)
1KO(E6.5)
DKO(E6.5)
TKO(E6.5)
E6.5
(Epiblast)
B
Nes (Nestin)
ICM-1
ICM-2
WT(E7.5)
1KO(E7.5)
DKO(E7.5)
TKO(E7.5)
E7.5
(Epiblast)
C
Nes (Nestin)
ICM-1
ICM-2
WT(E8.5)
1KO(E8.5)
DKO(E8.5)
E8.5
(Embryo)

Fig. S18

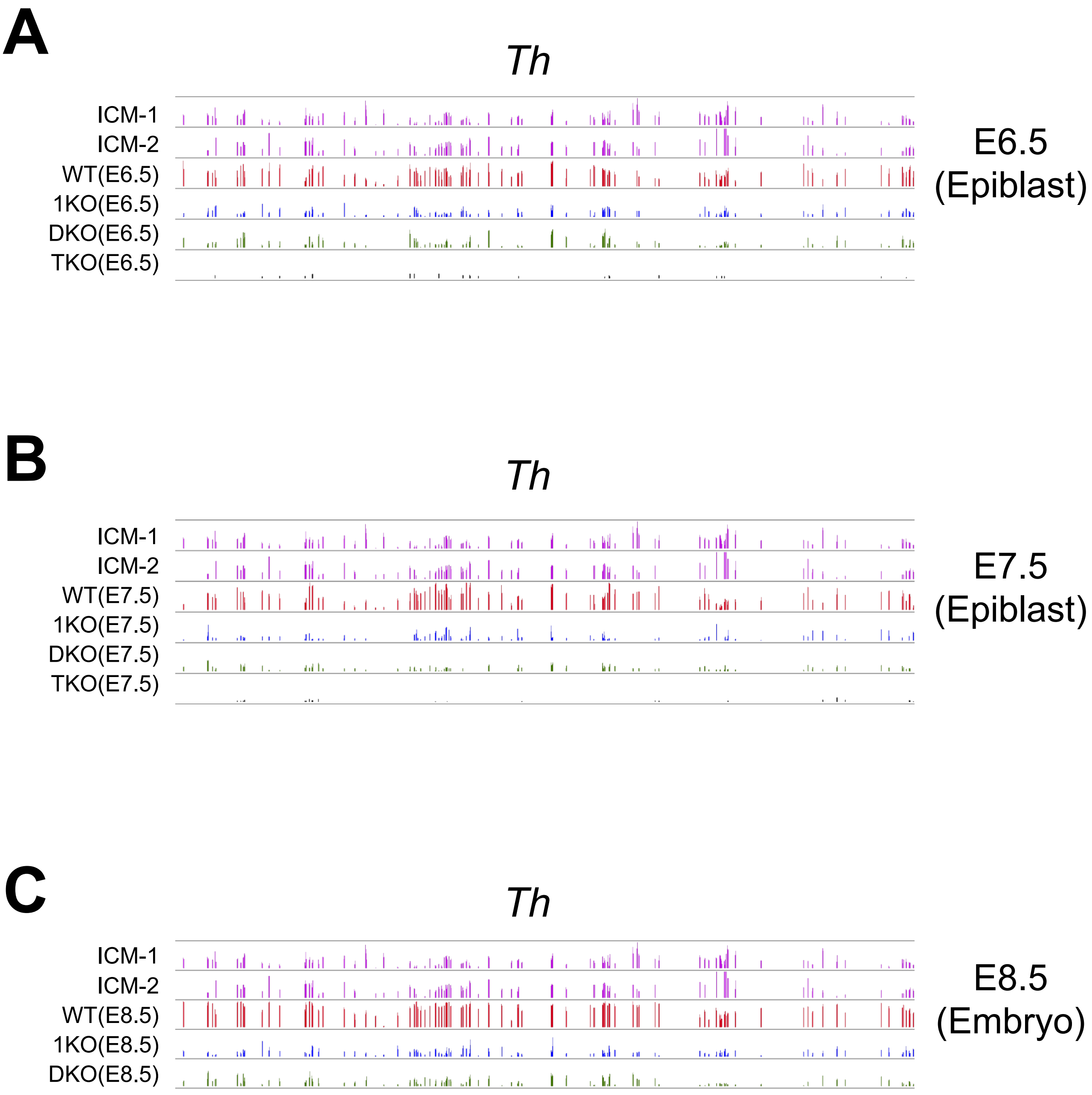
A
Th
ICM-1
ICM-2
WT(E6.5)
1KO(E6.5)
DKO(E6.5)
TKO(E6.5)
E6.5
(Epiblast)
B
Th
ICM-1
ICM-2
WT(E7.5)
1KO(E7.5)
DKO(E7.5)
TKO(E7.5)
E7.5
(Epiblast)
C
Th
ICM-1
ICM-2
WT(E8.5)
1KO(E8.5)
DKO(E8.5)
E8.5
(Embryo)

Fig. S19

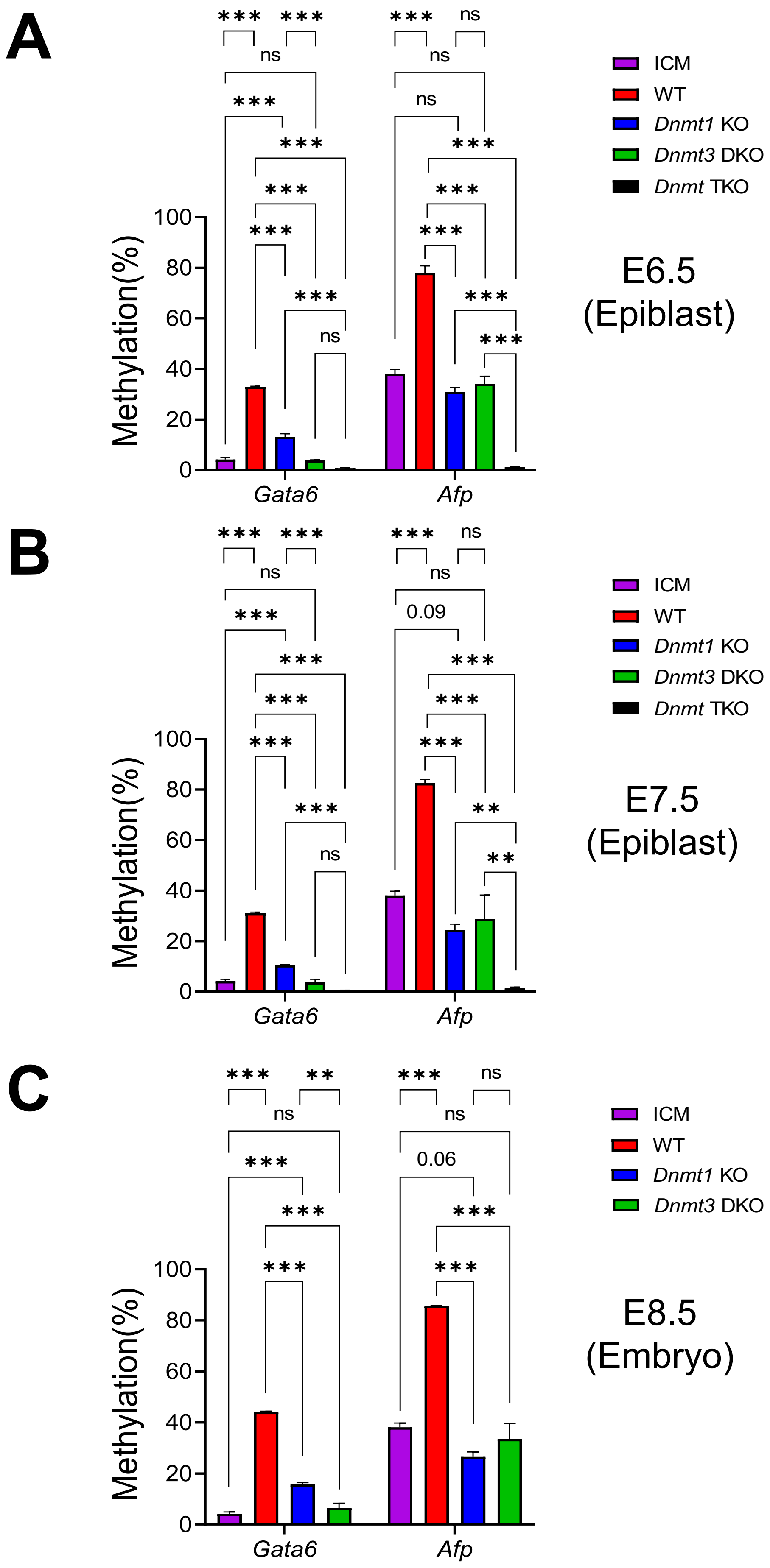
A
B
C
ICM
WT
Dnmt1 KO
Dnmt3 DKO
Dnmt TKO
E6.5
(Epiblast)
E7.5
(Epiblast)
E8.5
(Embryo)
Methylation(%)
100
80
60
40
20
0
Gata6
Afp
***
**
ns
0.09
0.06

Fig. S20

A

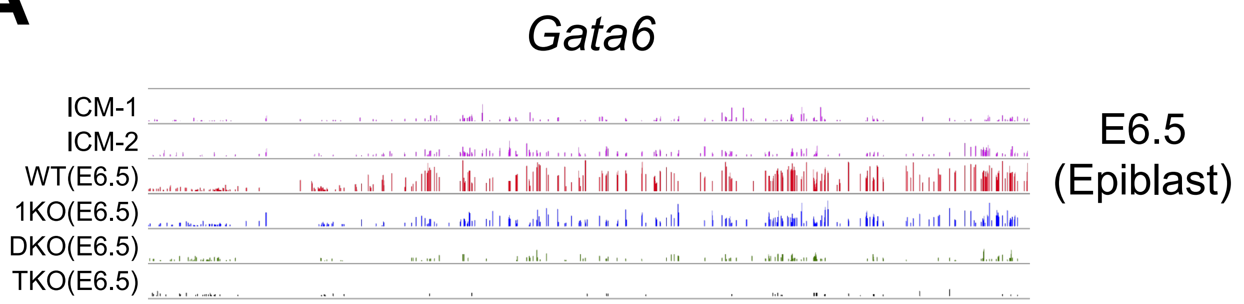
Gata6
ICM-1
ICM-2
WT(E6.5)
1KO(E6.5)
DKO(E6.5)
TKO(E6.5)
E6.5
(Epiblast)

B

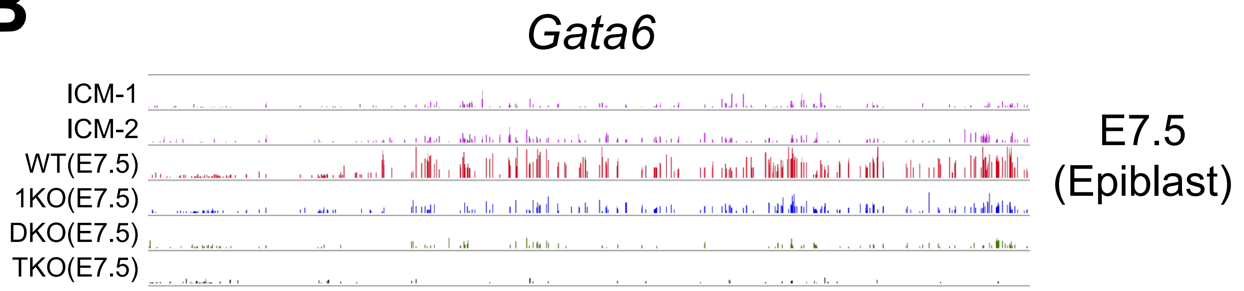
Gata6
ICM-1
ICM-2
WT(E7.5)
1KO(E7.5)
DKO(E7.5)
TKO(E7.5)
E7.5
(Epiblast)

C

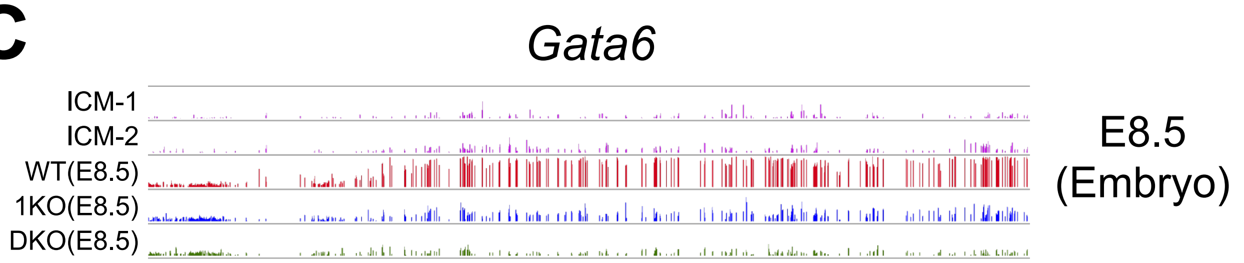
Gata6
ICM-1
ICM-2
WT(E8.5)
1KO(E8.5)
DKO(E8.5)
E8.5
(Embryo)

Fig. S21

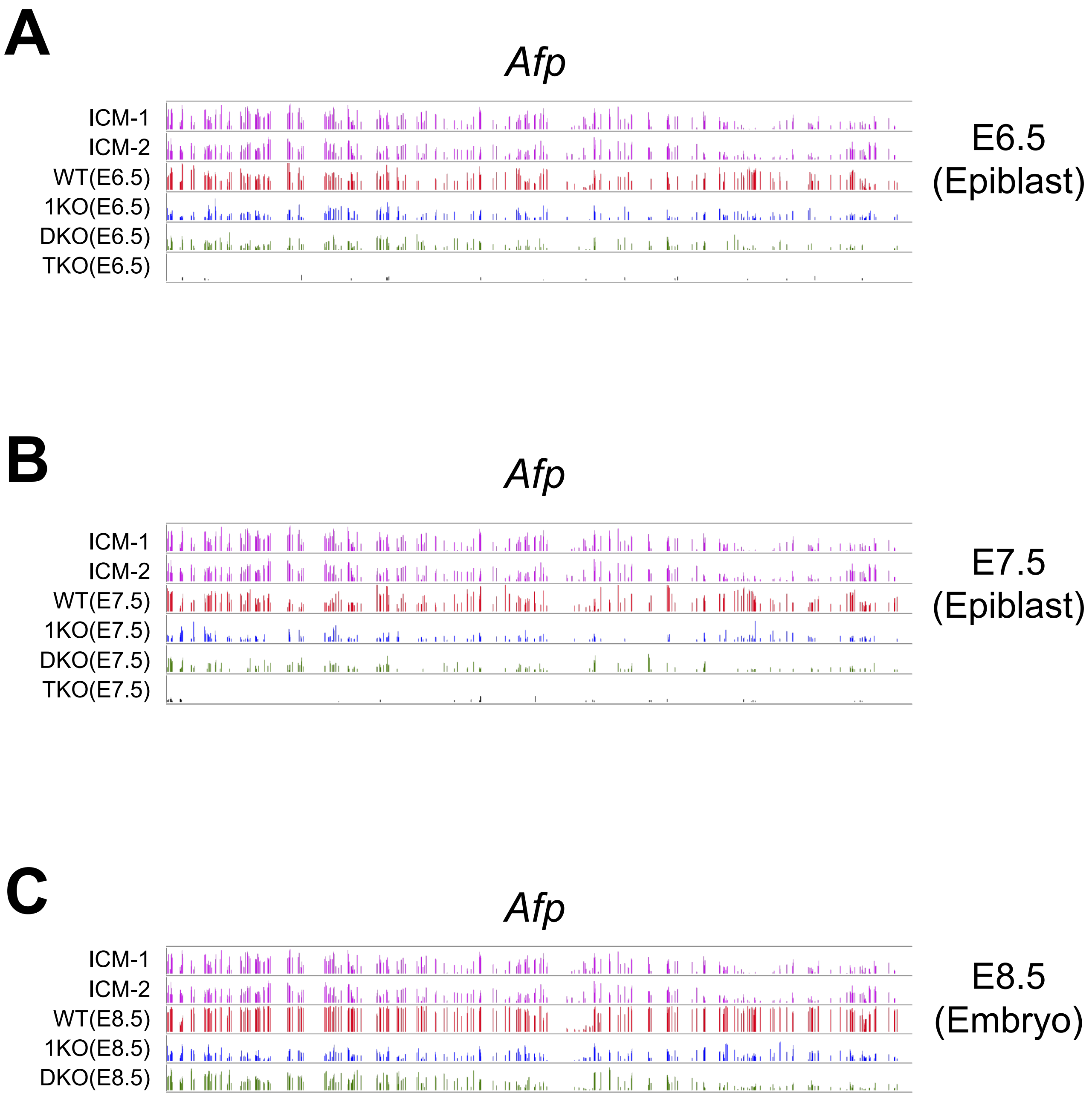
A
Afp
ICM-1
ICM-2
WT(E6.5)
1KO(E6.5)
DKO(E6.5)
TKO(E6.5)
E6.5
(Epiblast)
B
Afp
ICM-1
ICM-2
WT(E7.5)
1KO(E7.5)
DKO(E7.5)
TKO(E7.5)
E7.5
(Epiblast)
C
Afp
ICM-1
ICM-2
WT(E8.5)
1KO(E8.5)
DKO(E8.5)
E8.5
(Embryo)

Fig. S22
