# Supplementary material for: Regulation of de novo and maintenance DNA methylation by DNA methyltransferases in postimplantation embryos
Source: J Biol Chem. 2024 Nov 13;301(1):107990. doi: 10.1016/j.jbc.2024.107990 (PMC11742614; doi:10.1016/j.jbc.2024.107990)
Supplement: Manu-jbc-sup-24-rev1 [file mmc2.pdf]

Supporting Information

Regulation of de novo and maintenance DNA methylation by DNA methyltransferases in post-implantation embryos

Zhen Xu[1], Jiajia Shi[1,#], Qian Chen[1,#], Shuting Yang[1,#], Zilin Wang[1], Biao Xiao[1], Zhijian Lai[1], Yumeng, Jing[1], Yilin Li[1] and Xiajun Li[1, $,*]

[1]School of Life Science and Technology, ShanghaiTech University, Shanghai 201210, China

[#]Equal contribution

[$]Lead contact

* **Correspondence**: lixj1@shanghaitech.edu.cn

Supplemental Figure Legends

Figure S1. Two DNMT3 proteins, together with DNMT1, maintained DNA methylation at repeats and retroviral elements in the post-implantation embryos. DNA methylation was analyzed for the repeats and retroviral elements in the ICM of the wild-type (WT) E3.5 blastocysts, the epiblasts of WT and *Dnmt* mutant E6.5-E7.5 embryos, as well as WT and *Dnmt* mutant E8.5 embryos that were shown on the horizontal axis. Vertical axis, % of DNA methylation. A) DNA methylation was quantified for repetitive sequences in the ICM of WT blastocysts, the epiblasts of WT, *Dnmt1* mutant (*Dnmt1* KO), *Dnmt3* double mutant (*Dnmt3* DKO) and *Dnmt* triple mutant (*Dnmt* TKO) E6.5 embryos. B) DNA methylation was quantified for repetitive sequences in the ICM of WT blastocysts, the epiblasts of WT, *Dnmt1*

mutant (*Dnmt1* KO), *Dnmt3* double mutant (*Dnmt3* DKO) and *Dnmt* triple mutant (*Dnmt* TKO) E7.5 embryos. C) DNA methylation was quantified for repetitive sequences in the WT, *Dnmt1* mutant (*Dnmt1* KO), and *Dnmt3* double mutant (*Dnmt3* DKO) E8.5 embryos in comparison to the ICM of the wild-type blastocysts. Statistical analysis was carried out by using one-way ANOVA with Dunnett multiple comparison test. The values on the figures are shown as follows: *, p<0.05; **, p< 0.01; ***, p<0.001. ns, not statistically significant with p-value more than 0.1.

Figure S2. Two DNMT3 proteins, together with DNMT1, maintained DNA methylation in the intergenic regions after implantation. DNA methylation was analyzed for three intergenic regions (Intergenic 1, Intergenic 2 and Intergenic 3) in the ICM of the wild-type (WT) E3.5 blastocysts, the epiblasts of WT and *Dnmt* mutant E6.5-E7.5 embryos, as well as WT and *Dnmt* mutant E8.5 embryos. Vertical axis, % of DNA methylation. A) DNA methylation was analyzed for the WT ICM, the epiblasts of WT, *Dnmt1* mutant (*Dnmt1* KO), *Dnmt3* double mutant (*Dnmt3* DKO) and *Dnmt* triple mutant (*Dnmt* TKO) E6.5 embryos. B) DNA methylation was analyzed for the WT ICM, the epiblasts of WT, *Dnmt1* mutant (*Dnmt1* KO), *Dnmt3* double mutant (*Dnmt3* DKO) and *Dnmt* triple mutant (*Dnmt* TKO) E7.5 embryos. C) DNA methylation was quantified for the intergenic regions in the WT, *Dnmt1* mutant (*Dnmt1* KO), and *Dnmt3* double mutant (*Dnmt3* DKO) E8.5 embryos in comparison to the WT ICM. Statistical analysis was carried out by using one-way ANOVA with Dunnett multiple comparison test. The values on the figures are shown as follows: *, p<0.05; **, p< 0.01; ***, p<0.001. ns, not statistically significant with p-value more than 0.1.

Figure S3. Two DNMT3 proteins, together with DNMT1, maintained DNA methylation in the Intergenic 1 region in the post-implantation embryos according to the IGV plot. DNA methylation was analyzed for the individual CpG sites of the Intergenic 1 region in the ICM of the wild-type (WT) E3.5 blastocysts, the epiblasts of WT and *Dnmt* mutant E6.5-E7.5 embryos, as well as WT and *Dnmt* mutant E8.5 embryos. DNA methylation was significantly

reduced in the Intergenic 1 region in the *Dnmt3* DKO as well as D*nmt1* KO mutant post-implantation embryos based on the IGV plots of individual CpG sites. A) DNA methylation was analyzed for the Intergenic 1 region in the WT ICM, the epiblasts of WT, *Dnmt1* mutant (*Dnmt1* KO), *Dnmt3* double mutant (*Dnmt3* DKO) and *Dnmt* triple mutant (*Dnmt* TKO) E6.5 embryos. B) DNA methylation was analyzed for the Intergenic 1 region in the WT ICM, the epiblasts of WT, *Dnmt1* mutant (*Dnmt1* KO), *Dnmt3* double mutant (*Dnmt3* DKO) and *Dnmt* triple mutant (*Dnmt* TKO) E7.5 embryos. C) DNA methylation was analyzed for the Intergenic 1 region in the WT, *Dnmt1* mutant (*Dnmt1* KO), and *Dnmt3* double mutant (*Dnmt3* DKO) E8.5 embryos in comparison to the WT ICM.

Figure S4. Two DNMT3 proteins, together with DNMT1, maintained DNA methylation in the Intergenic 2 region in the post-implantation embryos according to the IGV plot. DNA methylation was analyzed for the individual CpG sites of the Intergenic 2 region in the ICM of the wild-type (WT) E3.5 blastocysts, the epiblasts of WT and *Dnmt* mutant E6.5-E7.5 embryos, as well as WT and *Dnmt* mutant E8.5 embryos. DNA methylation was significantly reduced in the Intergenic 2 region in the *Dnmt3* DKO as well as D*nmt1* KO mutant post-implantation embryos based on the IGV plots of individual CpG sites. A) DNA methylation was analyzed for the Intergenic 2 region in the WT ICM, the epiblasts of WT, *Dnmt1* mutant (*Dnmt1* KO), *Dnmt3* double mutant (*Dnmt3* DKO) and *Dnmt* triple mutant (*Dnmt* TKO) E6.5 embryos. B) DNA methylation was analyzed for the Intergenic 2 region in the WT ICM, the epiblasts of WT, *Dnmt1* mutant (*Dnmt1* KO), *Dnmt3* double mutant (*Dnmt3* DKO) and *Dnmt* triple mutant (*Dnmt* TKO) E7.5 embryos. C) DNA methylation was analyzed for the Intergenic 2 region in the WT, *Dnmt1* mutant (*Dnmt1* KO), and *Dnmt3* double mutant (*Dnmt3* DKO) E8.5 embryos in comparison to the WT ICM.

Figure S5. Two DNMT3 proteins, together with DNMT1, maintained DNA methylation in the Intergenic 3 region in the post-implantation embryos according to the IGV plot. DNA methylation was analyzed for the individual CpG sites of the Intergenic 3 region in the

ICM of the wild-type (WT) E3.5 blastocysts, the epiblasts of WT and *Dnmt* mutant E6.5-E7.5 embryos, as well as WT and *Dnmt* mutant E8.5 embryos. DNA methylation was significantly reduced in the Intergenic 3 region in the *Dnmt3* DKO as well as D*nmt1* KO mutant post-implantation embryos based on the IGV plots of individual CpG sites. A) DNA methylation was analyzed for the Intergenic 3 region in the WT ICM, the epiblasts of WT, *Dnmt1* mutant (*Dnmt1* KO), *Dnmt3* double mutant (*Dnmt3* DKO) and *Dnmt* triple mutant (*Dnmt* TKO) E6.5 embryos. B) DNA methylation was analyzed for the Intergenic 3 region in the WT ICM, the epiblasts of WT, *Dnmt1* mutant (*Dnmt1* KO), *Dnmt3* double mutant (*Dnmt3* DKO) and *Dnmt* triple mutant (*Dnmt* TKO) E7.5 embryos. C) DNA methylation was analyzed for the Intergenic 3 region in the WT, *Dnmt1* mutant (*Dnmt1* KO), and *Dnmt3* double mutant (*Dnmt3* DKO) E8.5 embryos in comparison to the WT ICM.

Figure S6. Two DNMT3 proteins, together with DNMT1, maintained DNA methylation in the CpG island regions after implantation. DNA methylation was analyzed for the CpG island (CGI) regions in the ICM of the wild-type (WT) E3.5 blastocysts, the epiblasts of WT and *Dnmt* mutant E6.5-E7.5 embryos, as well as WT and *Dnmt* mutant E8.5 embryos indicated on the horizontal axis. Vertical axis, % of DNA methylation in the CGI regions. A) DNA methylation was analyzed for the WT ICM, the epiblasts of WT, *Dnmt1* mutant (*Dnmt1* KO), *Dnmt3* double mutant (*Dnmt3* DKO) and *Dnmt* triple mutant (*Dnmt* TKO) E6.5 embryos. B) DNA methylation was analyzed for the WT ICM, the epiblasts of WT, *Dnmt1* mutant (*Dnmt1* KO), *Dnmt3* double mutant (*Dnmt3* DKO) and *Dnmt* triple mutant (*Dnmt* TKO) E7.5 embryos. C) DNA methylation was quantified for the CGI regions in the WT, *Dnmt1* mutant (*Dnmt1* KO), and *Dnmt3* double mutant (*Dnmt3* DKO) E8.5 embryos in comparison to the WT ICM. Statistical analysis was carried out by using one-way ANOVA with Dunnett multiple comparison test. The values on the figures are shown as follows: *, $p<0.05$; **, $p< 0.01$; ***, $p<0.001$. ns, not statistically significant with p-value more than 0.1.

Figure S7. DNA methylation was partially lost at a subset of ICRs in the post-implantation mutant embryos lacking DNMT3 proteins according to the IGV plot. DNA methylation was analyzed for the individual CpG sites of the IG-DMR of the *Dlk1-Dio3*, *Gpr1*, *Cdh15* and *H19* ICRs in the ICM of the wild-type (WT) E3.5 blastocysts, the epiblasts of WT and *Dnmt* mutant E6.5-E7.5 embryos, as well as WT and *Dnmt* mutant E8.5 embryos. DNA methylation was significantly reduced in these ICRs in the *Dnmt3* DKO as well as D*nmt1* KO mutant post-implantation embryos based on the IGV plots of individual CpG sites. A) DNA methylation was analyzed for these four ICRs in the WT ICM, the epiblasts of WT, *Dnmt1* mutant (*Dnmt1* KO), *Dnmt3* double mutant (*Dnmt3* DKO) and *Dnmt* triple mutant (*Dnmt* TKO) E6.5 embryos. B) DNA methylation was analyzed for these ICRs in the WT ICM, the epiblasts of WT, *Dnmt1* mutant (*Dnmt1* KO), *Dnmt3* double mutant (*Dnmt3* DKO) and *Dnmt* triple mutant (*Dnmt* TKO) E7.5 embryos. C) DNA methylation was analyzed for these ICRs in the WT, *Dnmt1* mutant (*Dnmt1* KO), and *Dnmt3* double mutant (*Dnmt3* DKO) E8.5 embryos in comparison to the WT ICM.

Figure S8. Two DNMT3 proteins, together with DNMT1, maintained pre-existing germline-derived DNA methylation at the *AK008011* imprinted region in the post-implantation embryos according to the IGV plot. DNA methylation was analyzed for the individual CpG sites of the *AK008011* imprinted region in the ICM of the wild-type (WT) E3.5 blastocysts, the epiblasts of WT and *Dnmt* mutant E6.5-E7.5 embryos, as well as WT and *Dnmt* mutant E8.5 embryos. DNA methylation was examined based on the previously defined ICR region (boxed areas) of the *AK008011* imprinted region (A-C). Since germline-derived differential DNA methylation appeared to be located in a more broad region at the *AK008011* imprinted region (boxed areas), we also analyzed DNA methylation of individual CpG sites in this broad *AK008011-Ex* ICR (A'-C'), which was also significantly reduced in the *Dnmt3* DKO as well as D*nmt1* KO mutant post-implantation embryos. A and A') DNA methylation at the *AK008011* imprinted region in the WT ICM, the epiblasts of WT, *Dnmt1* mutant (*Dnmt1* KO), *Dnmt3* double mutant (*Dnmt3* DKO) and *Dnmt* triple mutant (*Dnmt*

TKO) E6.5 embryos. B and B') DNA methylation at the *AK008011* imprinted region in the WT ICM, the epiblasts of WT, *Dnmt1* mutant (*Dnmt1* KO), *Dnmt3* double mutant (*Dnmt3* DKO) and *Dnmt* triple mutant (*Dnmt* TKO) E7.5 embryos. C and C') DNA methylation at the *AK008011* imprinted region in the WT, *Dnmt1* mutant (*Dnmt1* KO), and *Dnmt3* double mutant (*Dnmt3* DKO) E8.5 embryos in comparison to the WT ICM.

Figure S9. Two DNMT3 proteins, together with DNMT1, maintained pre-existing germline-derived DNA methylation at the *AK008011* imprinted region in the post-implantation embryos. DNA methylation in the *AK008011* imprinted region was analyzed for the genomic DNA samples derived from the ICM of the wild-type (WT) E3.5 blastocysts, the epiblasts of WT and *Dnmt* mutant E6.5-E7.5 embryos, as well as WT and *Dnmt* mutant E8.5 embryos. DNA methylation analysis was based on the previously defined *AK008011* ICR region (boxed areas)(A-C), and the more broad *AK008011-Ex* region (boxed areas) (A'-C'). A and A') DNA methylation at the *AK008011* imprinted region in the WT ICM, the epiblasts of WT, *Dnmt1* mutant (*Dnmt1* KO), *Dnmt3* double mutant (*Dnmt3* DKO) and *Dnmt* triple mutant (*Dnmt* TKO) E6.5 embryos. B and B') DNA methylation at the *AK008011* imprinted region in the WT ICM, the epiblasts of WT, *Dnmt1* mutant (*Dnmt1* KO), *Dnmt3* double mutant (*Dnmt3* DKO) and *Dnmt* triple mutant (*Dnmt* TKO) E7.5 embryos. C and C') DNA methylation at the *AK008011* imprinted region in the WT, *Dnmt1* mutant (*Dnmt1* KO), and *Dnmt3* double mutant (*Dnmt3* DKO) E8.5 embryos in comparison to the WT ICM. Statistical analysis was carried out by using one-way ANOVA with Dunnett multiple comparison test. The values on the figures are shown as follows: *, p<0.05; **, p< 0.01; ***, p<0.001. ns, not statistically significant with p-value more than 0.1.

Figure S10. DNMT3 proteins maintained pre-existing germline-derived DNA methylation at a subset of ICRs in the post-implantation embryos according to the IGV plot. DNA methylation was analyzed for the individual CpG sites of the *Gnas1A*, *Peg5*, *Mcts2* and *Slc38a4* ICRs in the ICM of the wild-type (WT) E3.5 blastocysts, the epiblasts of

WT and *Dnmt* mutant E6.5-E7.5 embryos, as well as WT and *Dnmt* mutant E8.5 embryos. DNA methylation was significantly reduced in these ICRs in the *Dnmt3* DKO as well as D*nmt1* KO mutant post-implantation embryos based on the IGV plots of individual CpG sites. A) DNA methylation was analyzed for these four ICRs in the WT ICM, the epiblasts of WT, *Dnmt1* mutant (*Dnmt1* KO), *Dnmt3* double mutant (*Dnmt3* DKO) and *Dnmt* triple mutant (*Dnmt* TKO) E6.5 embryos. B) DNA methylation was analyzed for these ICRs in the WT ICM, the epiblasts of WT, *Dnmt1* mutant (*Dnmt1* KO), *Dnmt3* double mutant (*Dnmt3* DKO) and *Dnmt* triple mutant (*Dnmt* TKO) E7.5 embryos. C) DNA methylation was analyzed for these ICRs in the WT, *Dnmt1* mutant (*Dnmt1* KO), and *Dnmt3* double mutant (*Dnmt3* DKO) E8.5 embryos in comparison to the WT ICM.

Figure S11. Two DNMT3 proteins, together with DNMT1, maintained DNA methylation at two pluripotent genes after implantation. DNA methylation at two pluripotent genes (*Pou5f1* (*Oct4*) and *Nanog*) was analyzed for the genomic DNA samples derived from the ICM of the wild-type (WT) E3.5 blastocysts, the epiblasts of WT and *Dnmt* mutant E6.5-E7.5 embryos, as well as WT and *Dnmt* mutant E8.5 embryos. Vertical axis, % of DNA methylation. A) DNA methylation was analyzed for the WT ICM, the epiblasts of WT, *Dnmt1* mutant (*Dnmt1* KO), *Dnmt3* double mutant (*Dnmt3* DKO) and *Dnmt* triple mutant (*Dnmt* TKO) E6.5 embryos. B) DNA methylation was analyzed for the WT ICM, the epiblasts of WT, *Dnmt1* mutant (*Dnmt1* KO), *Dnmt3* double mutant (*Dnmt3* DKO) and *Dnmt* triple mutant (*Dnmt* TKO) E7.5 embryos. C) DNA methylation was quantified for *Pou5f1* and *Nanog* in the WT, *Dnmt1* mutant (*Dnmt1* KO), and *Dnmt3* double mutant (*Dnmt3* DKO) E8.5 embryos in comparison to the WT ICM. Statistical analysis was carried out by using one-way ANOVA with Dunnett multiple comparison test. The values on the figures are shown as follows: *, $p<0.05$; **, $p< 0.01$; ***, $p<0.001$. ns, not statistically significant with p-value more than 0.1.

Figure S12. Three DNMT proteins maintained DNA methylation at *Pou5f1* in the post-implantation embryos according to the IGV plot. DNA methylation was analyzed for the

individual CpG sites at the *Pou5f1* (*Oct4*) gene in the ICM of the wild-type (WT) E3.5 blastocysts, the epiblasts of WT and *Dnmt* mutant E6.5-E7.5 embryos, as well as WT and *Dnmt* mutant E8.5 embryos. DNA methylation was significantly reduced at *Pou5f1* in the *Dnmt3* DKO as well as D*nmt1* KO mutant post-implantation embryos based on the IGV plots of individual CpG sites. A) DNA methylation was analyzed for the *Pou5f1* gene in the WT ICM, the epiblasts of WT, *Dnmt1* mutant (*Dnmt1* KO), *Dnmt3* double mutant (*Dnmt3* DKO) and *Dnmt* triple mutant (*Dnmt* TKO) E6.5 embryos. B) DNA methylation was analyzed for the *Pou5f1* gene in the WT ICM, the epiblasts of WT, *Dnmt1* mutant (*Dnmt1* KO), *Dnmt3* double mutant (*Dnmt3* DKO) and *Dnmt* triple mutant (*Dnmt* TKO) E7.5 embryos. C) DNA methylation was analyzed for the *Pou5f1* gene in the WT, *Dnmt1* mutant (*Dnmt1* KO), and *Dnmt3* double mutant (*Dnmt3* DKO) E8.5 embryos in comparison to the WT ICM.

Figure S13. Three DNMT proteins maintained DNA methylation at *Nanog* in the post-implantation embryos according to the IGV plot. DNA methylation was analyzed for the individual CpG sites at the *Nanog* gene in the ICM of the wild-type (WT) E3.5 blastocysts, the epiblasts of WT and *Dnmt* mutant E6.5-E7.5 embryos, as well as WT and *Dnmt* mutant E8.5 embryos. DNA methylation was significantly reduced at *Nanog* in the *Dnmt3* DKO as well as D*nmt1* KO mutant post-implantation embryos based on the IGV plots of individual CpG sites. A) DNA methylation was analyzed for the *Nanog* gene in the WT ICM, the epiblasts of WT, *Dnmt1* mutant (*Dnmt1* KO), *Dnmt3* double mutant (*Dnmt3* DKO) and *Dnmt* triple mutant (*Dnmt* TKO) E6.5 embryos. B) DNA methylation was analyzed for the *Nanog* gene in the WT ICM, the epiblasts of WT, *Dnmt1* mutant (*Dnmt1* KO), *Dnmt3* double mutant (*Dnmt3* DKO) and *Dnmt* triple mutant (*Dnmt* TKO) E7.5 embryos. C) DNA methylation was analyzed for the *Nanog* gene in the WT, *Dnmt1* mutant (*Dnmt1* KO), and *Dnmt3* double mutant (*Dnmt3* DKO) E8.5 embryos in comparison to the WT ICM.

Figure S14. Three DNMT proteins maintained DNA methylation at two genes in the mesoderm development in the post-implantation embryos. DNA methylation at two

genes in the mesoderm development (*Brachyury* (*T*) and *Tnnt2*) was analyzed for the genomic DNA samples derived from the ICM of the wild-type (WT) E3.5 blastocysts, the epiblasts of WT and *Dnmt* mutant E6.5-E7.5 embryos, as well as WT and *Dnmt* mutant E8.5 embryos. Vertical axis, % of DNA methylation. A) DNA methylation was analyzed for the WT ICM, the epiblasts of WT, *Dnmt1* mutant (*Dnmt1* KO), *Dnmt3* double mutant (*Dnmt3* DKO) and *Dnmt* triple mutant (*Dnmt* TKO) E6.5 embryos. B) DNA methylation was analyzed for the WT ICM, the epiblasts of WT, *Dnmt1* mutant (*Dnmt1* KO), *Dnmt3* double mutant (*Dnmt3* DKO) and *Dnmt* triple mutant (*Dnmt* TKO) E7.5 embryos. C) DNA methylation was quantified for *Brachyury* (*T*) and *Tnnt2* in the WT, *Dnmt1* mutant (*Dnmt1* KO), and *Dnmt3* double mutant (*Dnmt3* DKO) E8.5 embryos in comparison to the WT ICM. Statistical analysis was carried out by using one-way ANOVA with Dunnett multiple comparison test. The values on the figures are shown as follows: *, $p<0.05$; **, $p< 0.01$; ***, $p<0.001$. ns, not statistically significant with p-value more than 0.1.

Figure S15. Three DNMT proteins maintained DNA methylation at the *Brachyury* gene in the post-implantation embryos according to the IGV plot. DNA methylation was analyzed for the individual CpG sites at the *Brachyury* (*T*) gene in the mesoderm development in the ICM of the wild-type (WT) E3.5 blastocysts, the epiblasts of WT and *Dnmt* mutant E6.5-E7.5 embryos, as well as WT and *Dnmt* mutant E8.5 embryos. DNA methylation was significantly reduced at *Brachyury* (*T*) in the *Dnmt3* DKO as well as D*nmt1* KO mutant post-implantation embryos based on the IGV plots of individual CpG sites. A) DNA methylation was analyzed for the *Brachyury* (*T*) gene in the WT ICM, the epiblasts of WT, *Dnmt1* mutant (*Dnmt1* KO), *Dnmt3* double mutant (*Dnmt3* DKO) and *Dnmt* triple mutant (*Dnmt* TKO) E6.5 embryos. B) DNA methylation was analyzed for the *Brachyury* (*T*) gene in the WT ICM, the epiblasts of WT, *Dnmt1* mutant (*Dnmt1* KO), *Dnmt3* double mutant (*Dnmt3* DKO) and *Dnmt* triple mutant (*Dnmt* TKO) E7.5 embryos. C) DNA methylation was analyzed for the *Brachyury* (*T*) gene in the WT, *Dnmt1* mutant (*Dnmt1* KO), and *Dnmt3* double mutant (*Dnmt3* DKO) E8.5 embryos in comparison to the WT ICM.

Figure S16. Three DNMT proteins maintained DNA methylation at the *Tnnt2* gene in the post-implantation embryos according to the IGV plot. DNA methylation was analyzed for the individual CpG sites at the *Tnnt2* gene in the mesoderm development in the ICM of the wild-type (WT) E3.5 blastocysts, the epiblasts of WT and *Dnmt* mutant E6.5-E7.5 embryos, as well as WT and *Dnmt* mutant E8.5 embryos. DNA methylation was significantly reduced at *Tnnt2* in the *Dnmt3* DKO as well as D*nmt1* KO mutant post-implantation embryos based on the IGV plots of individual CpG sites. A) DNA methylation was analyzed for the *Tnnt2* gene in the WT ICM, the epiblasts of WT, *Dnmt1* mutant (*Dnmt1* KO), *Dnmt3* double mutant (*Dnmt3* DKO) and *Dnmt* triple mutant (*Dnmt* TKO) E6.5 embryos. B) DNA methylation was analyzed for the *Tnnt2* gene in the WT ICM, the epiblasts of WT, *Dnmt1* mutant (*Dnmt1* KO), *Dnmt3* double mutant (*Dnmt3* DKO) and *Dnmt* triple mutant (*Dnmt* TKO) E7.5 embryos. C) DNA methylation was analyzed for the *Tnnt2* gene in the WT, *Dnmt1* mutant (*Dnmt1* KO), and *Dnmt3* double mutant (*Dnmt3* DKO) E8.5 embryos in comparison to the WT ICM.

Figure S17. Three DNMT proteins maintained DNA methylation at two genes in the ectoderm development in the post-implantation embryos. DNA methylation at two genes in the ectoderm development (*Nestin* (*Nes*) and *Th*) was analyzed for the genomic DNA samples derived from the ICM of the wild-type (WT) E3.5 blastocysts, the epiblasts of WT and *Dnmt* mutant E6.5-E7.5 embryos, as well as WT and *Dnmt* mutant E8.5 embryos. Vertical axis, % of DNA methylation. A) DNA methylation was analyzed for the WT ICM, the epiblasts of WT, *Dnmt1* mutant (*Dnmt1* KO), *Dnmt3* double mutant (*Dnmt3* DKO) and *Dnmt* triple mutant (*Dnmt* TKO) E6.5 embryos. B) DNA methylation was analyzed for the WT ICM, the epiblasts of WT, *Dnmt1* mutant (*Dnmt1* KO), *Dnmt3* double mutant (*Dnmt3* DKO) and *Dnmt* triple mutant (*Dnmt* TKO) E7.5 embryos. C) DNA methylation was quantified for *Nes* and *Th* in the WT, *Dnmt1* mutant (*Dnmt1* KO), and *Dnmt3* double mutant (*Dnmt3* DKO) E8.5 embryos in comparison to the WT ICM. Statistical analysis was carried out by using one-way

ANOVA with Dunnett multiple comparison test. The values on the figures are shown as follows: *, $p<0.05$; **, $p< 0.01$; ***, $p<0.001$. ns, not statistically significant with p-value more than 0.1.

Figure S18. Three DNMT proteins maintained DNA methylation at the *Nestin* gene in the post-implantation embryos according to the IGV plot. DNA methylation was analyzed for the individual CpG sites at the *Nestin* (*Nes*) gene in the ectoderm development in the ICM of the wild-type (WT) E3.5 blastocysts, the epiblasts of WT and *Dnmt* mutant E6.5-E7.5 embryos, as well as WT and *Dnmt* mutant E8.5 embryos. DNA methylation was significantly reduced at *Nes* in the *Dnmt3* DKO as well as D*nmt1* KO mutant post-implantation embryos based on the IGV plots of individual CpG sites. A) DNA methylation was analyzed for the *Nes* gene in the WT ICM, the epiblasts of WT, *Dnmt1* mutant (*Dnmt1* KO), *Dnmt3* double mutant (*Dnmt3* DKO) and *Dnmt* triple mutant (*Dnmt* TKO) E6.5 embryos. B) DNA methylation was analyzed for the *Nes* gene in the WT ICM, the epiblasts of WT, *Dnmt1* mutant (*Dnmt1* KO), *Dnmt3* double mutant (*Dnmt3* DKO) and *Dnmt* triple mutant (*Dnmt* TKO) E7.5 embryos. C) DNA methylation was analyzed for the *Nes* gene in the WT, *Dnmt1* mutant (*Dnmt1* KO), and *Dnmt3* double mutant (*Dnmt3* DKO) E8.5 embryos in comparison to the WT ICM.

Figure S19. Three DNMT proteins maintained DNA methylation at the *Th* gene in the post-implantation embryos according to the IGV plot. DNA methylation was analyzed for the individual CpG sites at the *Th* gene in the ectoderm development in the ICM of the wild-type (WT) E3.5 blastocysts, the epiblasts of WT and *Dnmt* mutant E6.5-E7.5 embryos, as well as WT and *Dnmt* mutant E8.5 embryos. DNA methylation was significantly reduced at *Th* in the *Dnmt3* DKO as well as D*nmt1* KO mutant post-implantation embryos based on the IGV plots of individual CpG sites. A) DNA methylation was analyzed for the *Th* gene in the WT ICM, the epiblasts of WT, *Dnmt1* mutant (*Dnmt1* KO), *Dnmt3* double mutant (*Dnmt3* DKO) and *Dnmt* triple mutant (*Dnmt* TKO) E6.5 embryos. B) DNA methylation was analyzed

for the *Th* gene in the WT ICM, the epiblasts of WT, *Dnmt1* mutant (*Dnmt1* KO), *Dnmt3* double mutant (*Dnmt3* DKO) and *Dnmt* triple mutant (*Dnmt* TKO) E7.5 embryos. C) DNA methylation was analyzed for the *Th* gene in the WT, *Dnmt1* mutant (*Dnmt1* KO), and *Dnmt3* double mutant (*Dnmt3* DKO) E8.5 embryos in comparison to the WT ICM.

Figure S20. Three DNMT proteins maintained DNA methylation at two genes in the endoderm development in the post-implantation embryos. DNA methylation at two genes in the endoderm development (*Gata6* and *Afp*) was analyzed for the genomic DNA samples derived from the ICM of the wild-type (WT) E3.5 blastocysts, the epiblasts of WT and *Dnmt* mutant E6.5-E7.5 embryos, as well as WT and *Dnmt* mutant E8.5 embryos. Vertical axis, % of DNA methylation. A) DNA methylation was analyzed for the WT ICM, the epiblasts of WT, *Dnmt1* mutant (*Dnmt1* KO), *Dnmt3* double mutant (*Dnmt3* DKO) and *Dnmt* triple mutant (*Dnmt* TKO) E6.5 embryos. B) DNA methylation was analyzed for the WT ICM, the epiblasts of WT, *Dnmt1* mutant (*Dnmt1* KO), *Dnmt3* double mutant (*Dnmt3* DKO) and *Dnmt* triple mutant (*Dnmt* TKO) E7.5 embryos. C) DNA methylation was quantified for *Gata6* and *Afp* in the WT, *Dnmt1* mutant (*Dnmt1* KO), and *Dnmt3* double mutant (*Dnmt3* DKO) E8.5 embryos in comparison to the WT ICM. Statistical analysis was carried out by using one-way ANOVA with Dunnett multiple comparison test. The values on the figures are shown as follows: *, $p<0.05$; **, $p< 0.01$; ***, $p<0.001$. ns, not statistically significant with p-value more than 0.1.

Figure S21. Three DNMT proteins maintained DNA methylation at the *Gata6* gene in the post-implantation embryos according to the IGV plot. DNA methylation was analyzed for the individual CpG sites at the *Gata6* gene in the endoderm development in the ICM of the wild-type (WT) E3.5 blastocysts, the epiblasts of WT and *Dnmt* mutant E6.5-E7.5 embryos, as well as WT and *Dnmt* mutant E8.5 embryos. DNA methylation was significantly reduced at *Gata6* in the *Dnmt3* DKO as well as D*nmt1* KO mutant post-implantation embryos based on the IGV plots of individual CpG sites. A) DNA methylation was analyzed

for the *Gata6* gene in the WT ICM, the epiblasts of WT, *Dnmt1* mutant (*Dnmt1* KO), *Dnmt3* double mutant (*Dnmt3* DKO) and *Dnmt* triple mutant (*Dnmt* TKO) E6.5 embryos. B) DNA methylation was analyzed for the *Gata6* gene in the WT ICM, the epiblasts of WT, *Dnmt1* mutant (*Dnmt1* KO), *Dnmt3* double mutant (*Dnmt3* DKO) and *Dnmt* triple mutant (*Dnmt* TKO) E7.5 embryos. C) DNA methylation was analyzed for the *Gata6* gene in the WT, *Dnmt1* mutant (*Dnmt1* KO), and *Dnmt3* double mutant (*Dnmt3* DKO) E8.5 embryos in comparison to the WT ICM.

Figure S22. Three DNMT proteins maintained DNA methylation at the *Afp* gene in the post-implantation embryos according to the IGV plot. DNA methylation was analyzed for the individual CpG sites at the *Afp* gene in the endoderm development in the ICM of the wild-type (WT) E3.5 blastocysts, the epiblasts of WT and *Dnmt* mutant E6.5-E7.5 embryos, as well as WT and *Dnmt* mutant E8.5 embryos. DNA methylation was significantly reduced at *Afp* in the *Dnmt3* DKO as well as D*nmt1* KO mutant post-implantation embryos based on the IGV plots of individual CpG sites. A) DNA methylation was analyzed for the *Afp* gene in the WT ICM, the epiblasts of WT, *Dnmt1* mutant (*Dnmt1* KO), *Dnmt3* double mutant (*Dnmt3* DKO) and *Dnmt* triple mutant (*Dnmt* TKO) E6.5 embryos. B) DNA methylation was analyzed for the *Afp* gene in the WT ICM, the epiblasts of WT, *Dnmt1* mutant (*Dnmt1* KO), *Dnmt3* double mutant (*Dnmt3* DKO) and *Dnmt* triple mutant (*Dnmt* TKO) E7.5 embryos. C) DNA methylation was analyzed for the *Afp* gene in the WT, *Dnmt1* mutant (*Dnmt1* KO), and *Dnmt3* double mutant (*Dnmt3* DKO) E8.5 embryos in comparison to the WT ICM.
